# Identification of stomatal-regulating molecules from de novo arylamine collection through aromatic C–H amination

**DOI:** 10.1038/s41598-022-04947-z

**Published:** 2022-01-18

**Authors:** Yosuke Toda, Gregory J. P. Perry, Shimpei Inoue, Eri Ito, Takahiro Kawakami, Mina R. Narouz, Koji Takahashi, Yusuke Aihara, Bumpei Maeda, Toshinori Kinoshita, Kenichiro Itami, Kei Murakami

**Affiliations:** 1 JST PRESTO, 7 Gobancho, Chiyoda, Tokyo, 102-0076 Japan; 2 Institute of Transformative Bio-Molecules (WPI-ITbM), Nagoya University, Chikusa, Nagoya, 464-8602 Japan; 3 Graduate School of Science, Nagoya University, Chikusa, Nagoya, 464-8602 Japan; 4 Department of Chemistry, School of Science, Kwansei Gakuin University, Sanda, Hyogo 669-1337 Japan

**Keywords:** Chemical biology, Chemical libraries

## Abstract

Stomata—small pores generally found on the leaves of plants—control gas exchange between plant and the atmosphere. Elucidating the mechanism that underlies such control through the regulation of stomatal opening/closing is important to understand how plants regulate photosynthesis and tolerate against drought. However, up-to-date, molecular components and their function involved in stomatal regulation are not fully understood. We challenged such problem through a chemical genetic approach by isolating and characterizing synthetic molecules that influence stomatal movement. Here, we describe that a small chemical collection, prepared during the development of C–H amination reactions, lead to the discovery of a Stomata Influencing Molecule (SIM); namely, a sulfonimidated oxazole that inhibits stomatal opening. The starting molecule SIM1 was initially isolated from screening of compounds that inhibit light induced opening of dayflower stomata. A range of SIM molecules were rapidly accessed using our state-of-the-art C–H amination technologies. This enabled an efficient structure–activity relationship (SAR) study, culminating in the discovery of a sulfonamidated oxazole derivative (SIM*) having higher activity and enhanced specificity against stomatal regulation. Biological assay results have shed some light on the mode of action of SIM molecules within the cell, which may ultimately lead to drought tolerance-conferring agrochemicals through the control of stomatal movement.

## Introduction

Stomata consist of a pair of guard cells in the plant epidermis and control gas exchange between plant and the atmosphere. Stomatal aperture facilitates CO_2_ uptake from the atmosphere for photosynthesis but also water loss from plant tissue, and thus, stomata have an important role in balancing between photosynthetic efficiency and drought tolerance. Plants control the degree of stomatal opening (stomatal regulation) in response to various stimuli (such as light, drought, CO_2_, or toxins) in order to adapt to the surrounding environment^1–5^. To date, genetic factors that help regulate stomatal aperture have been successfully identified through various approaches, including forward and reverse genetics, as well as bio-chemical and physiological research. These provided insight into the molecular mechanisms underlying stomatal opening, exemplified by the discovery of key genetic components of blue-light mediated signal transduction pathway^6–12^. Recently, the utilization of chemicals that can temporarily influence the activity of genes, such as those involved in both stomatal regulation and developmental process, has been gaining interest as a strategy to overcome obstacles associated with genetic functional redundancy^13–15^. Thereby, this approach has allowed the identification of novel factors which may have been overlooked by the aforesaid approaches^16–18^. Previous studies using chemical libraries have revealed small molecules as biological tools that can aid in deciphering the molecular mechanisms that control stomata associated phenotypes^19–23^. A more detailed understanding of these mechanisms would not only have a profound effect in the field, but can ultimately lead to crop yield increase or drought tolerance-conferring agrochemicals.

**Figure 1:**
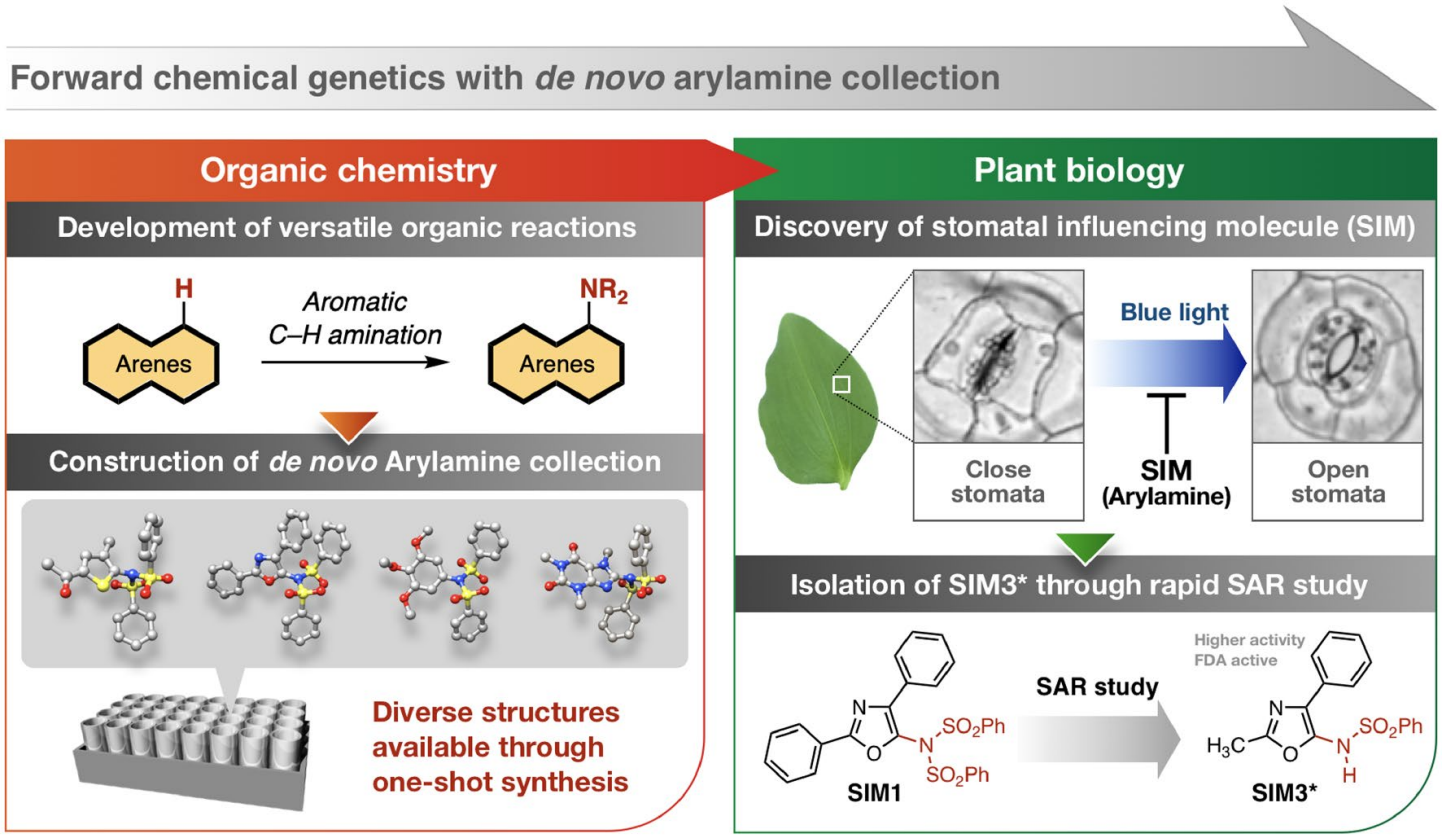
Overview of this study.

Among a variety of chemicals, arylamines have been recognized as privileged molecules for aromatics with biological activity^24–28^, such as pharmaceuticals and agrochemicals. Generally, the amine moiety increases the number of potential hydrogen bond that may form with the target as well as change the characteristics of arene structures that might otherwise be planar and non-polar. Thus, the amine moiety may provide arene molecules with an increased affinity for protein interaction^26,27^. The development of efficient methods for arylamine synthesis has long been explored. In recent years, the field of direct C–H amination has grown significantly as it represents an ideal and streamlined method for constructing useful arylamines from simple and readily available arenes (Fig. 1, left)^29–34^. The aromatic C–H amination reaction can modify the properties of the corresponding aromatic cores. Since 2015, we have reported versatile catalytic aromatic C–H amination reactions that allow diversification of a variety of aromatics^35–37^. We envisioned that small chemical collections derived from reaction-driven research, i.e. C–H amination, could aid existing chemical genetic approaches for studying stomatal movement (Fig. 1, right) and provide an alternative approach to identifying bioactive molecules which complements existing strategies, for example diversity-oriented synthesis (DOS) and biology-oriented synthesis (BIOS)^38–41^. In this report, we show that small chemical collections prepared from the development of new C–H functionalization methodologies reveal small molecules that display bioactivity. We discover a range of aminated oxazole (Stomata Influencing Molecules; SIM) compounds that are able to suppress the light-induced opening of stomata. Advantageously, reaction-driven chemical libraries allow the rapid derivatization of a lead molecule for SAR studies, leading to a more bioactive and selective stomata regulating molecule, SIM3*.

## Results and discussion

### Discovery of stomatal influencing molecule: SIM1

**Figure 2:**
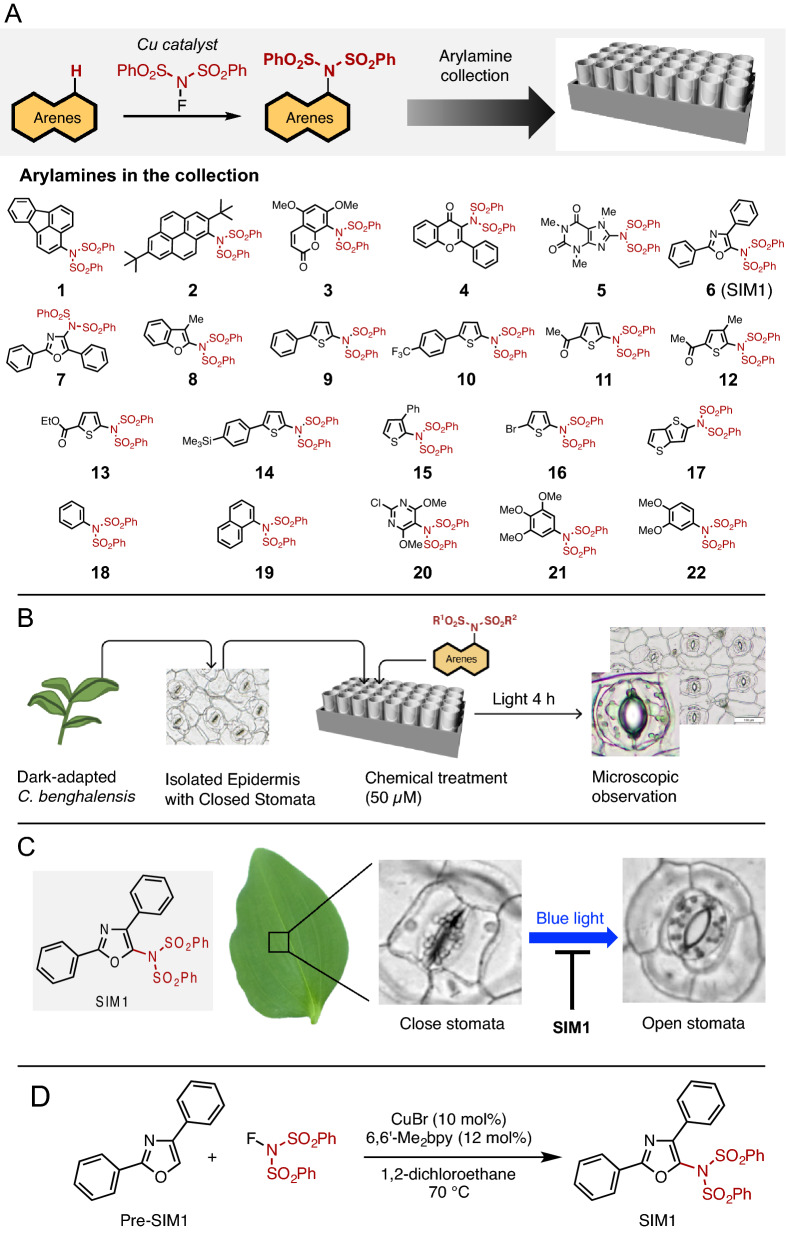
(**A**) Construction of arylamine collection. (**B**) Schematic illustration of the chemical screen of chemicals that inhibit stomatal opening. (**C**) Isolation of SIM1 as a stomatal regulating molecule. (**D**) Synthesis of SIM1.

In this research, we constructed a C–H amination-based arylamine collection to evaluate its potential in identifying bioactive compounds through a phenotype-based screening. First, a variety of arylamines were efficiently prepared by a copper-catalyzed coupling of arenes with the aminating agent, *N*-fluorobenzenesulfonimide (NFSI). This one-step synthesis^35^ provided our initial arylamine collection of 22 products (Fig. 2A). Using this collection, we screened for compounds that inhibit the light-induced opening of stomata in dayflower (*Commelina benghalensis*), following the previously successful study of isolating promising compounds where the initial screening was done by qualitative evaluation (Fig. 2B), (see “Methods” section for details)^23^. As a result, we accomplished the successful identification of the lead candidate SIM1 (Stomata Influencing Molecule 1), a sulfonimidated 2,4-diphenyloxazole **6** (Fig. 2C). Notably, the isomer, sulfonimidated 2,5-diphenyloxazole **7**, showed no activity towards inhibition, indicating the structural specificity of SIM1 for its bioactivity. We wish to emphasize that preparation of SIM1 is easy: one-step C–H amination from inexpensive and commercially available reagents/catalyst (Fig. 2D). These findings have shown that fundamental chemistry research into reaction methodologies can yield a library of molecules with unique bioactivity.

### Studies on molecular action of SIM1

**Figure 3:**
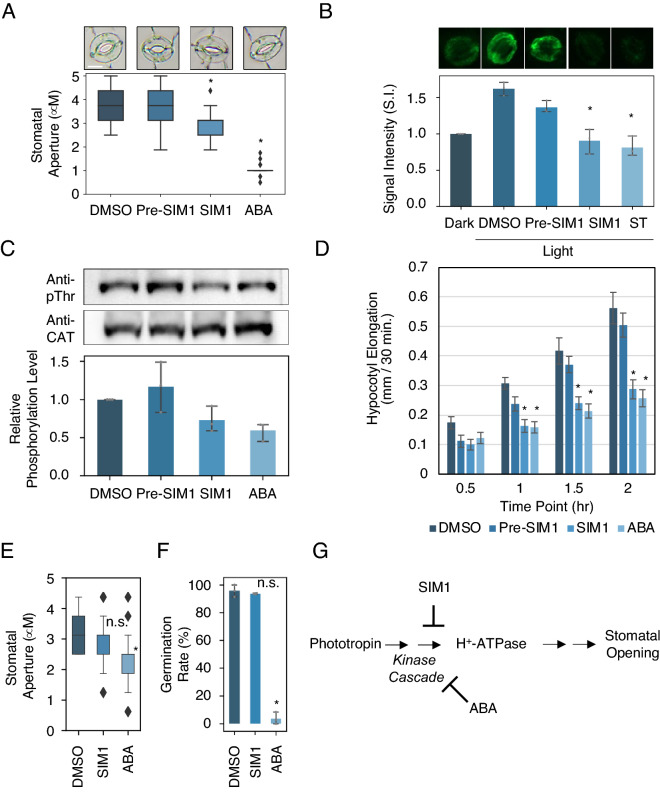
SIM1 inhibits promotion of H^+^-ATPase phosphorylation in *Arabidopsis thaliana*. (**A**) Inhibition of light induced stomatal opening by the treatment of SIM1 in *A. thaliana*. Stomatal apertures were measured after 3 h of light irradiation in the presence of the indicated compounds. Representative appearances of stomata are shown in the top row. DMSO, 0.5% Dimethyl sulfoxide, mock control; Pre-SIM1, 50 µM Non sulfonimidated form of SIM1; ABA, 10 µM Abscisic acid, positive control. Scalebar = 10 µm. Asterisk indicates statistical significance compared to DMSO treatment (student t-test p < 0.05). Diamonds indicate outliers. Means ± SD (*n* > 45). If not specified, compounds were treated at the concentration of 50 µM. (**B**) Detection of phosphorylated H^+^-ATPase in guard cells of *A. thaliana* as determined by immuno-histochemical staining using the antibody raised against phosphorylated penultimate Thr (anti-pThr). Representative stained stomata are displayed in the upper row. Prior to irradiation, samples were treated with the indicated compounds for 30 min. *Dark*, 20 min of darkness; *Light*, 20 min of red light (50 μmol m^−2^ s^−1^) followed by 2.5 min of blue light (10 μmol m^−2^ s^−1^) irradiation superimposed on background red light. Light treatment increases the amount of phosphorylated H^+^-ATPase at 1.5-fold (Dark to Light DMSO), while SIM1 and ST (50 µM Staurosporine, the broad-spectrum kinase inhibitor as positive control) treatment suppresses such increase. Means ± SD (*n* = 3; 29 < stomata per replicate). (**C**) Detection of phosphorylated H^+^-ATPase in *Arabidopsis* hypocotyl of 4 days-old seedling as determined by immunoblot using anti-pThr and anti-CAT which the latter recognizes the catalytic domain of PM H^+^-ATPase. Seedlings were treated with the indicated compounds for 2 h before sampling. For better visualization, blot images were cropped. See Supplementary Fig. S1 for full view. Means ± SD (*n* = 3). (**D**) Quantification of hypocotyl elongation in *A. thaliana* under respective compound treatment. Means ± SE (*n* = 4; 10 seedlings per replicate). (**E**) Promotion of stomatal closure against opened stomata in *A. thaliana*. First, the isolated epidermal peels were irradiated with light for 4 h in buffer, and treated with respective compounds, and further irradiated for 3 h prior to stomatal aperture quantification. Diamonds in the boxplots represent outliers. n.s., no statistically significant difference compared to DMSO treatment (student t-test p > 0.05). Means ± SD (*n* = 45). (**F**) Seed germination tests using *Arabidopsis* seeds. Seeds were immersed in water containing respective chemicals and were incubated in dark for 5 days, and ratios of germinated seeds were calculated. Means ± SD (*n* = 3, 24 to 50 seeds per replicate). (**G**) Simplified graphical abstract of the mode of action of SIM1 along with that of ABA in stomatal opening signaling. phototropin, a blue-light receptor kinase that triggers stomatal opening^7^.

To investigate the molecular action of SIM1, we performed further analysis in *Arabidopsis*, a model plant. SIM1 was able to inhibit the light induced stomatal opening of *Arabidopsis* (Fig. 3A). Meanwhile, Pre-SIM1 (the non-imidated diphenyloxazole shown in Fig. 2D) had no such effect, indicating that sulfonimidation via C–H functionalization confers novel activity to simple aromatic structures. In guard cells, which constitute the stomatal complex, activation of the plasma membrane (PM) H^+^-ATPase via phosphorylation of a penultimate threonine residue (pT) is known to play a central role in stomatal opening^1,5^. To determine whether SIM1 influences such activation, we performed immunological analysis to detect the phosphorylation of pT of PM H^+^-ATPase in guard cells. As shown in Fig. 3B, phosphorylation was observed in response to light treatment, however this effect was diminished in the presence of SIM1. Similar effects were also observed in *Arabidopsis* hypocotyl, where it has been reported that PM H^+^-ATPase promotes cell elongation^42^. For example, SIM1 suppressed both the degree of PM H^+^-ATPase phosphorylation and elongation in the hypocotyl (Fig. 3C,D). These results suggest that SIM1 inhibits factors that are involved in regulating PM H^+^-ATPase phosphorylation of the penultimate threonine amino acid residue in the plasma membrane, i.e. suppressing the activation of PM H^+^-ATPase and, therefore, the opening of stomata. These characteristics of SIM1 resemble that of the naturally occurring plant hormone abscisic acid (ABA), which also inhibits phosphorylation and the opening of stomata^43^ (Fig. 3A,C). However, whereas ABA is able to actively promote closure of stomata, SIM1 proved inactive (i.e. SIM1 can inhibit stomatal opening of closed stomata, but it cannot promote stomatal closing of open stomata, Fig. 3E). Furthermore, SIM1 did not have the function to inhibit seed germination, in contrast to the previously reported function of ABA^44^ (Fig. 3F). This suggests that SIM1 does not inhibit light-induced stomatal opening through a typical ABA signaling pathway, but may rather selectively inhibit that of blue-light induced stomatal opening signaling (Fig. 3G). Future biochemical and genetic studies will further clarify the mechanism of action including the potential molecular target of SIM1.

**Figure 4:**
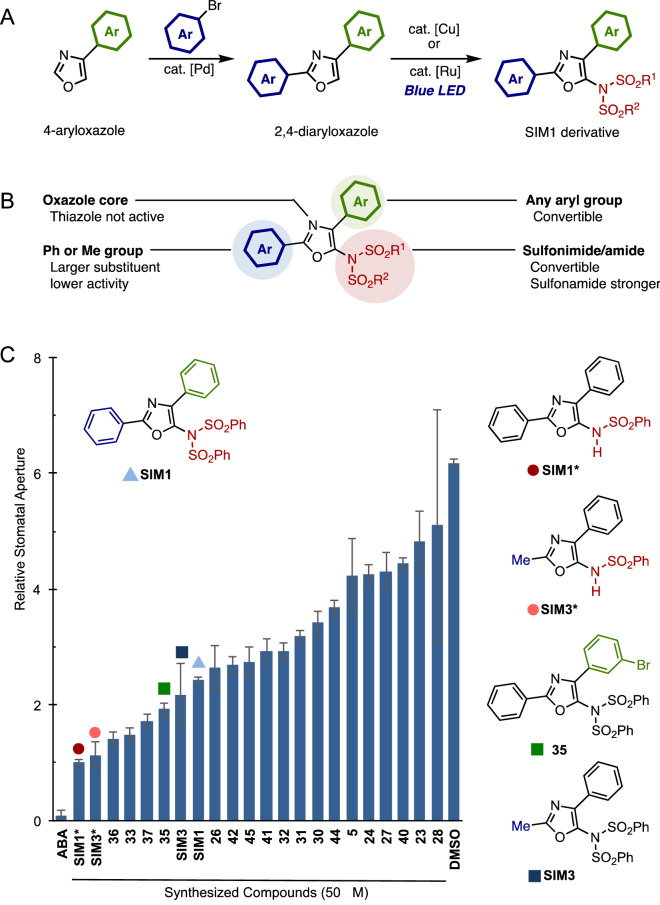
Synthesis and evaluation of SIM1 derivatives for structural activity relationship (SAR) study. (**A**) Synthesis of SIM derivatives. (**B**) Summary of SAR study. (**C**) Inhibition of light induced stomatal opening by SIM1 derivatives evaluated in *C. benghalensis*. Stomatal apertures were measured after 3 h of light irradiation in the presence of the indicated compounds (50 µM). Values are relative to values of SIM1* treated stomatal apertures. *ABA* 10 µM Abscisic acid, positive control. Mean ± SD (n > 50).

**Figure 5:**
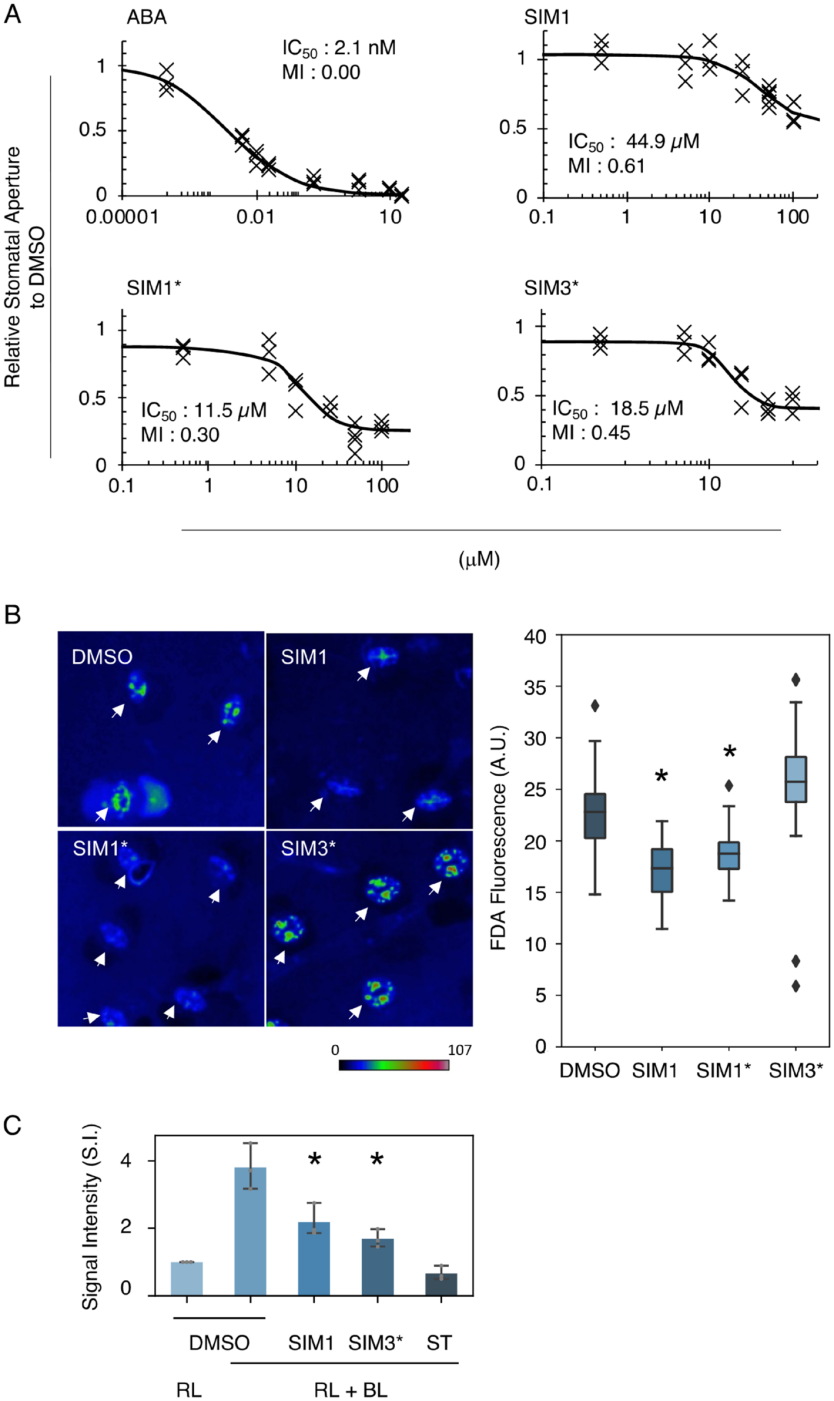
Evaluation of SIM1 derivatives for structural activity relationship (SAR) study. (**A**) Dose dependent inhibition of stomatal opening of *C. benghalensis* by ABA, SIM1, SIM1*, and SIM3*, respectively. Stomatal apertures were measured after 3 h of light irradiation in the presence of the indicated compounds in varying concentration. Half inhibition concentration (IC_50_) and maximum inhibition (MI) values of each compounds are displayed. Values are relative to DMSO treatment. (n = 60 stomata per replicate). (**B**) Evaluation of cellular esterase activity in guard cells of *C. benghalensis* visualized by fluorescein diacetate (FDA) staining after 50 µM compound treatment for 20 min. Images are displayed in pseudo-color. Arrows indicate the position of stomata. The raw outputs of each image are shown in Supplemental Fig. S2. Representative appearance of the epidermis is shown on the left, while its quantification values are shown as a boxplot on the right (*n* = 60). Asterisk indicates statistical significance compared to DMSO treatment (student t-test p < 0.05). Diamonds in the boxplots represent outliers. (**C**) Detection of phosphorylated PM H^+^-ATPase in guard cells of *A. thaliana* as determined by immuno-histochemical staining using the anti-pThr. Means ± SE (*n* = 3, 40 < stomata for each replicate). ST, Staurosporine. Asterisk indicates statistical significance DMSO and SIM1 and SIM3* treatment, respectively (student t-test p < 0.05). Prior to irradiation, samples were treated with the indicated compounds (50 µM) for 30 min. *RL* 20 min of red light (50 μmol m^−2^ s^−1^) irradiation, *RL* + *BL* 20 min of red light (50 μmol m^−2^ s^−1^) followed by 2.5 min of blue light (10 μmol m^−2^ s^−1^) irradiation superimposed on background RL.

In order to conduct a structure–activity relationship (SAR) study, we prepared a collection of SIM1 analogues to elucidate the effect of molecular sizes, conformations, and the number of coordination sites. The advantages of our direct C–H amination reaction were made apparent when preparing derivatives for the SAR study as, we were able to efficiently prepare a range of derivatives in-house and at will. The full range of the synthesized SIM1 analogues were synthesized by 2-step C–H functionalization, which is shown in the supplementary information (Fig. 4A). At first, 2,4-diaryloxazole derivatives were synthesized by treating 4-aryloxazoles with aryl bromides in the presence of a palladium catalyst. Then, exposing these oxazoles to our C–H amination reaction provided a range of SIM1 derivatives. Figure 4B summarizes the result of the SAR study on SIM1 derivatives. Modification of the SIM1 substructure had a diverse effect against its bioactivity (Fig. 4C). Key to SIM1’s bioactivity is the oxazole core, for example, a similar thiazole derivative was not active. The 2-position of the oxazole ring could be substituted with a phenyl group or smaller groups (e.g. –H (**30**) or –Me (SIM3)), and higher bioactivity was observed with a methyl group (SIM3). For the 4-position, all aryl substituents tested showed similar bioactivity. For example, bromo-substituted **35**, which can undergo a variety of useful chemical transformations, displayed bioactivity (Fig. 4C). Current studies in our group are focusing on the installment of linkers/tags through reaction at the bromo-position for use in target protein identification. During our study, we found that desulfonylated SIM1 (SIM1*) resulted in the strongest activity towards inhibition of stomatal opening (Figs. 4C, 5A). However, we observed that both SIM1 and SIM1* lowered the staining activity by fluorescein diacetate (FDA) in guard cells (Fig. 5B, Supplementary Fig. S1), which acts as a substrate of cellular esterase and is therefore utilized for guard cell viability evaluation^23,45–47^. Further SAR studies successfully identified SIM3* (Fig. 4C) as a stomatal influencing molecule. This compound displayed strong inhibition activity towards stomatal opening but retained the ability for FDA staining and the inhibition of blue-light induced PM H^+^-ATPase phosphorylation (Fig. 5A–C). These results suggest that SIM3* shows greater selectivity on the stomatal opening cascade compared with SIM1 and SIM1*. Compared to SIM1, SIM3* is characterized by the absence of a mono-sulfonyl group and the introduction of a methyl moiety (Fig. 1). Therefore, the different selectivity may be due to changes in the affinity or accessibility of potential target proteins by physical changes in the binding site. Future studies using SIM3* derivatives as a biological tool are expected to further elucidate the mechanisms surrounding stomatal regulation. Notably, we have found that the inhibitory effect of SIM3* is diminished by the addition of fungal toxin, fusicoccin, a direct activator of PM H^+^-ATPase^48^ (Fig. S3). It is thus implying that SIM3* affects the signaling component upstream of the PM H^+^-ATPase phosphorylation (Fig. 3G).

## Summary

Our research into C–H amination technologies has allowed us to construct a chemical library, from which we have identified a set of molecules (SIM/SIM*) that inhibit stomatal opening. The results highlight the power of reaction-driven forward chemical genetics and we believe that a host of small molecules with potentially unique bioactivities are possible through reaction development^49,50,51^. Our future work will consist of continued and more detailed studies to better understand how SIM molecules affect stomatal aperture. Further demonstration of the power of small molecules for assessing biological mechanisms is ongoing.

## Methods

### Experimental procedures for studies on organic chemistry

#### Procedure for the synthesis of SIM1 derivatives through Cu-catalyzed C–H imidation^35^


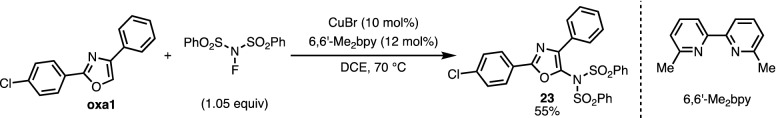


The synthesis of **23** is representative: 2-(4-Chlorophenyl)-4-phenyloxazole **oxa1** (51 mg, 0.20 mmol), NFSI (66 mg, 0.21 mmol, 1.05 equiv), CuBr (2.9 mg, 0.020 mmol, 10 mol%) and 6,6'-Me_2_bpy (4.4 mg, 0.024 mmol, 12 mol%) were added to a Schlenk tube in the open air. The tube was filled with nitrogen by employing a usual Schlenk technique (evacuate-refill cycle). 1,2-Dichloroethane (1.0 mL) was added to the tube and the mixture was heated at 70 °C for 12 h in the closed system. The mixture was then cooled to 25 °C. The crude solution was filtered through a pad of silica gel (ca. 6 g) and Na_2_SO_4_ (ca. 20 g) in a column and concentrated *in vacuo*. Purification by chromatography on silica-gel (*n*-hexane/EtOAc = 5:1 to 4:1) followed by recrystallization from CH_2_Cl_2_/MeOH provided **23** (61 mg, 0.11 mmol, 55%).
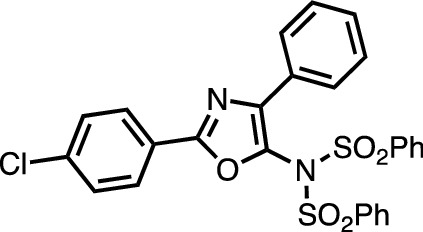


##### *N*-[2-(4-chlorophenyl)-4-phenyloxazol-5-yl]-*N*-(phenylsulfonyl)benzenesulfonamide (23)

^1^H NMR (CDCl_3_) δ 7.18 (t, *J* = 7.2 Hz, 2H), 7.23 (t, *J* = 7.2 Hz, 1H), 7.44–7.47 (m, 6H), 7.62 (t, *J* = 7.8 Hz, 2H), 7.69 (d, *J* = 7.2 Hz, 2H), 7.92 (d, *J* = 7.8 Hz, 2H), 7.96 (d, *J* = 7.8 Hz, 4H); ^13^C NMR (CDCl_3_) δ 125.47, 126.98, 128.15, 128.62, 129.11, 129.16, 129.23, 129.43, 133.45, 134.74, 137.71, 139.25, 141.02, 159.81 (one sp^2^ peak was not observed because of overlapping.); HR-MS (ESI–MS, positive): *m/z* = 573.0317. calcd for C_27_H_19_ClN_2_O_5_S_2_Na: 573.0316 [*M* + Na]^+^.

#### Procedure for the synthesis of SIM1 derivatives through photoredox-catalyzed C–H imidation^36^


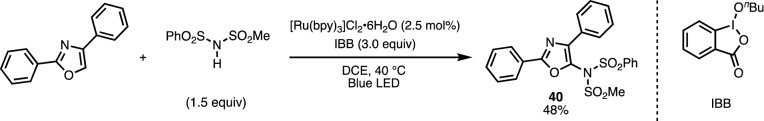


The synthesis of **40** is representative: 2,4-Diphenyloxazole (44 mg, 0.20 mmol), *N*-(methylsulfonyl)benzenesulfonamide (71 mg, 0.30 mmol, 1.5 equiv), IBB (192 mg, 0.60 mmol, 3.0 equiv), and [Ru(bpy)_3_]Cl_2_·6H_2_O (3.7 mg, 0.005 mmol, 2.5 mol%) were added to a Schlenk tube in the open air. The tube was filled with nitrogen by employing a usual Schlenk technique (evacuate-refill cycle). 1,2-Dichloroethane (2.0 mL) was added to the tube and the mixture was stirred with shedding blue light (three 2.88 W blue LED strips were located 3 cm away from the reaction vials) for 24 h in the closed system. The reaction temperature was 25 °C at the beginning and gradually increased to 40 °C by irradiation of blue light. The crude solution was diluted with EtOAc (10 mL), then 1 M NaOH (10 mL) was added. The organic phase was extracted using EtOAc. The combined organic layers were washed with brine and dried over anhydrous Na_2_SO_4_. The solvent was concentrated *in vacuo*. Purification by chromatography on silica-gel (*n*-hexane/EtOAc = 5:1 to 4:1) followed by recrystallization from CH_2_Cl_2_/MeOH provided **40** (43 mg, 0.095 mmol, 48%).
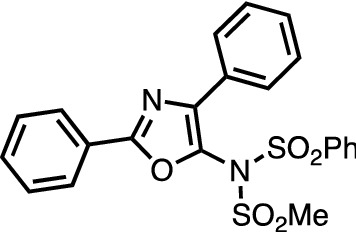


##### *N*-(2,4-diphenyloxazol-5-yl)-*N*-(methylsulfonyl)benzenesulfonamide (40)

^1^H NMR (CDCl_3_) δ 3.51 (s, 3H), 7.25–7.29 (m, 3H), 7.37 (app t, *J* = 7.8 Hz, 2H), 7.45–7.51 (m, 3H), 7.56 (t, *J* = 7.8 Hz, 1H), 7.76 (dd, *J* = 7.8 Hz, 2.4 Hz, 2H), 7.86 (d, *J* = 7.8 Hz, 2H), 8.06 (d, *J* = 7.8 Hz, 2H); ^13^C NMR (CDCl_3_) δ 44.78, 126.92, 126.98, 127.13, 128.80, 129.05, 129.11, 129.13, 129.35, 129.43, 131.54, 132.91, 134.83, 136.66, 140.67, 160.82; HR-MS (ESI–MS, positive): *m/z* = 477.0547 calcd for C_22_H_18_N_2_O_5_S_2_Na: 477.0549 [*M* + Na]^+^.

### Experimental procedures for studies on biology

#### Plant materials and growth conditions

*Commelina benghalensis* were grown in a greenhouse as described previously^38^. *Arabidopsis thaliana* plants were grown at 22 °C under a photoperiod of 16 h white light (50 μmol m^−2^ s^−1^)/8 h dark. The authors confirm that the present study complies with the IUCN Policy Statement on Research Involving Species at Risk of Extinction and the Convention on the Trade in Endangered Species of Wild Fauna and Flora.

#### Chemical library screening

We screened a total of 22 compounds from the homemade sulfonimide compound collection. Compounds were dissolved in dimethyl sulfoxide (DMSO) at a concentration of 10 mM. Each compound was added at a 1:200 dilution to basal buffer (5 mM MES/bistrispropane [pH 6.5], 50 mM KCl, and 0.1 mM CaCl_2_) for a final concentration of 50 µM. *C. benghalensis* plants were first incubated in the dark overnight to ensure complete closure of stomata prior to assay. Using such plants, under dim light, 1–2 cm^2^ epidermal peels were excised from fully expanded leaves using scissors and forceps. The epidermal peels were immersed in the buffer containing chemical compounds and were incubated under fluorescent white light (50 μmol m^−2^ s^−1^) or in the dark at 25 °C for 4 h, and samples in which the stomata were uniformly closed were identified using a stereoscopic microscope (Stereo Discovery; Zeiss, Oberkochen, Germany).

#### Measurement of stomatal apertures

Samples of *C. benghalensis* were prepared as described above, while epidermal fraction of fully expanded rosette leaves from 5 to 7-week-old plants of *Arabidopsis thaliana* was prepared as described previously^23^. For the light irradiation, 50 µmol m^−2^ s^−1^ red light and 10 µmol m^−2^ s^−1^ blue light were used instead of white light. After treatment of compounds in varying concentration, images were acquired using an optical microscope (BX43; Olympus, Tokyo, Japan) with a charge-coupled device (CCD) camera (DP27; Olympus) with a × 10 objective lens (UPlanFL N; Olympus). Stomatal apertures of respective stomata were measured using FLUOVIEW software (Olympus).

#### Quantification of the degree of H^+^-ATPase phosphorylation

Phosphorylation of PM H^+^-ATPase in hypocotyls or guard cells from the epidermis of *Arabidopsis* was determined by immunoblot or by immunofluorescence, respectively, as described previously^42,43^.

#### Fluorescein diacetate staining assay

Fluorescein Diacetate Staining Assay to determine to guard cell viability of *C. benghalensis* was determined as described previously^23^.

## Supplementary Information

Supplementary Information.
