# Supplementary material for: Identification of stomatal-regulating molecules from de novo arylamine collection through aromatic C–H amination
Source: Sci Rep. 2022 Jan 18;12:949. doi: 10.1038/s41598-022-04947-z (PMC8766585; doi:10.1038/s41598-022-04947-z)
Supplement: Supplementary file 1 — Supplementary Information. [file 41598_2022_4947_MOESM1_ESM.pdf]

Supplementary Information

Identification of stomatal-regulating molecules from *de novo* arylamine collection through aromatic C–H amination

Yosuke Toda,[1,2]* Gregory J. P. Perry[2†], Shimpei Inoue[3†], Eri Ito[3†], Takahiro Kawakami[3], Mina Narouz[3], Koji Takahashi,[3] Yusuke Aihara,[3] Bumpei Maeda,[4] Toshinori Kinoshita,[2,3] Kenichiro Itami,[2,3] Kei Murakami[1–4]*

[1] JST PRESTO, 7 Gobancho, Chiyoda, Tokyo, 102-0076 Japan

[2] Institute of Transformative Bio-Molecules (WPI-ITbM), Nagoya University, Chikusa, Nagoya 464-8602, Japan

[3] Graduate School of Science, Nagoya University, Chikusa, Nagoya, 464-8602 Japan

[4] Department of Chemistry, School of Science, Kwansei Gakuin University, Sanda, Hyogo 669-1337, Japan

[†]Equal contribution.

Table of Contents

General	S2
The aromatic sulfonimide library	S3
Experimental procedures for studies on biology	S5
Experimental procedures for studies on organic chemistry	S9
Characterization data	S14
^{1}H and ^{13}C NMR Spectra	S25

General

Unless otherwise noted, all reactants or reagents, including dry solvents, were obtained from commercial suppliers and used as received. Formamide, anhydrous 1,4-dioxane, anhydrous 1,2-dichloroethane, anhydrous acetonitrile and 1,2-ethylenediamine was purchased from KANTO chemical. Lithium *tert*-butoxide and $[Ru(bpy)_3]Cl_2 \cdot 6H_2O$ were purchased from Aldrich. Silver triflate, anhydrous ethyl acetate, CuBr, and *N*-fluorobenzenesulfonimide (NFSI) were purchased from Wako. 6,6'-Dimethyl-2,2'-bipyridyl was purchased from Wako or Aldrich. 2,4-Dimethyloxazole and diphenylsulfonimide was purchased from TCI. DDQ was purchased from KANTO chemical or TCI. All reactions were performed with dry solvents. All work-up and purification procedures were carried out with reagent-grade solvents. Analytical thin-layer chromatography (TLC) was performed using E. Merck silica gel 60 F254 precoated plates (0.25 mm). The developed chromatogram was analyzed by UV lamp (254 nm) or phosphomolybdic acid/sulfuric acid solution. Flash column chromatography was performed with E. Merck silica gel 60 (230–400 mesh). Silica-gel column chromatography was performed on an Isolera Spektra instrument equipped with a Biotage SNAP Ultra 10 g cartridge for 0.2 mmol scale reactions. The high-resolution mass spectra were recorded on JEOL JMS-T100GCV. Nuclear magnetic resonance (NMR) spectra were recorded on a JEOL JNM-ECA-600/400 (^{1}H 400 or 600 MHz, ^{13}C 150 MHz) spectrometer. Chemical shifts are expressed in parts per million (ppm) relative to tetramethylsilane (^{1}H NMR, δ 0.00 ppm in $CDCl_3$) or the residual deuterated solvent signal of $CDCl_3$ (^{1}H NMR, δ 7.26 ppm; ^{13}C NMR, δ 77.2 ppm) or CD_2Cl_2 (^{1}H NMR, δ 5.32 ppm; ^{13}C NMR, δ 53.8 ppm). Data are reported as follows: chemical shift, multiplicity (s = singlet, d = doublet, dd = doublet of doublets, t = triplet, dt = doublet of triplets, td = triplet of doublets, q = quartet, m = multiplet, brs = broad singlet, app t = apparent triplet), coupling constant (Hz), and integration.

The aromatic sulfonimide library

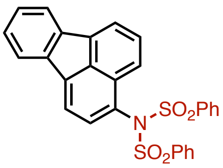

1

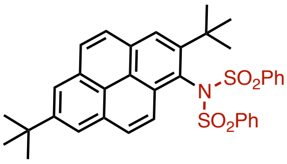

2

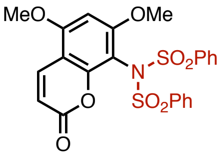

3

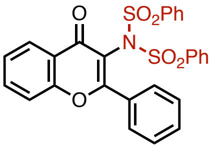

4

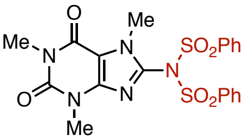

5

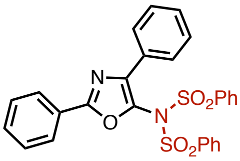

6 (SIM1)

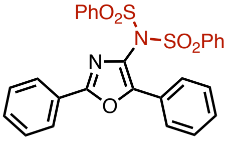

7

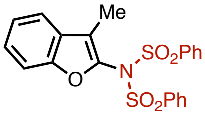

8

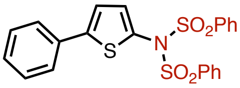

9

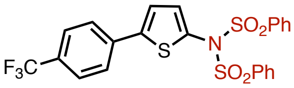

10

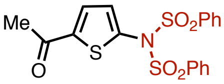

11

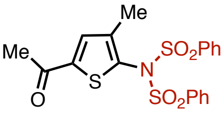

12

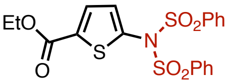

13

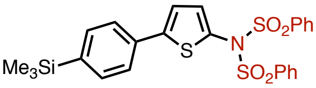

14

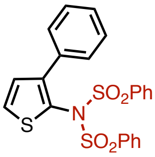

15

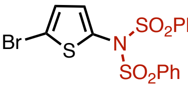

16

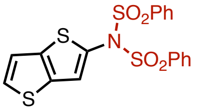

17

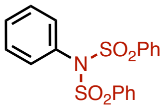

18

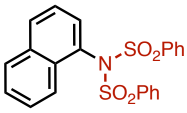

19

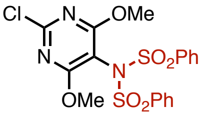

20

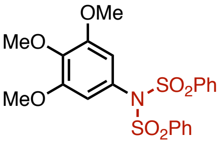

21

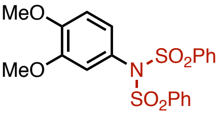

22

Table S 1. First-set of aromatic sulfonimide collection.

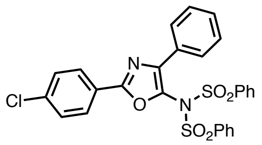

23

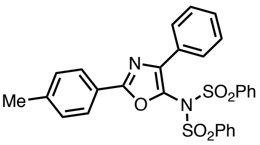

24

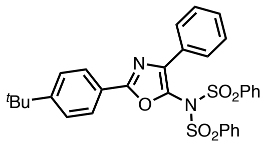

25

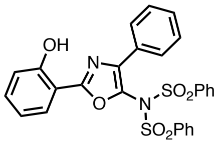

26

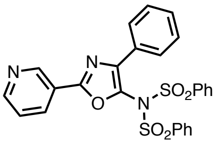

27

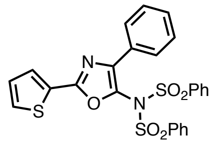

28

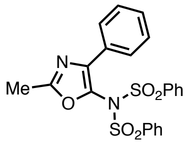

29

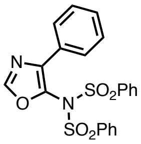

30

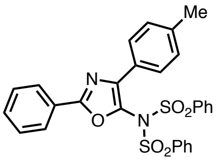

31

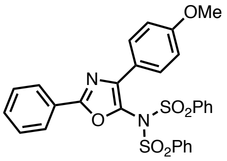

32

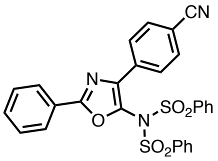

33

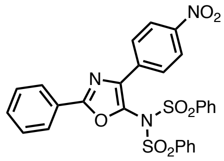

34

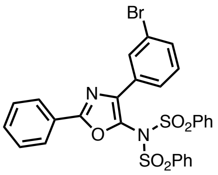

35

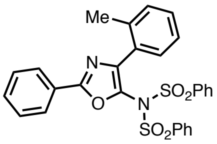

36

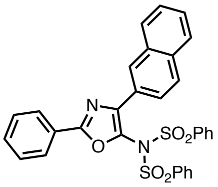

37

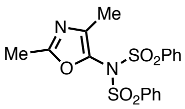

38

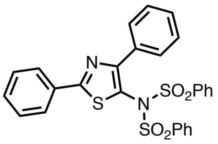

39

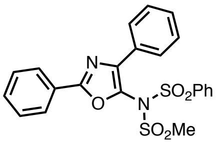

40

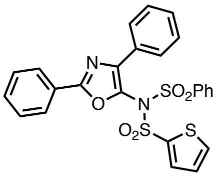

41

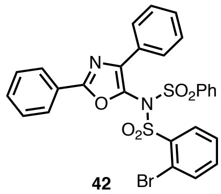

42

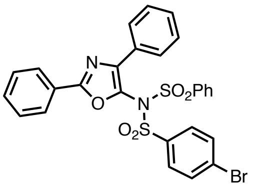

43

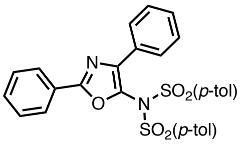

44

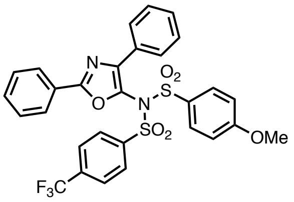

45

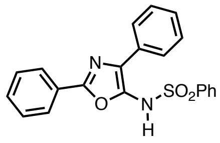

46
(SIM1*)

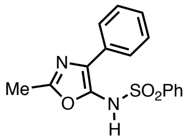

47
(SIM3*)

Table S 2. Second-set of SIM1 derivatives library

Experimental procedures for studies on biology

Plant growth conditions

Commelina benghalensis were grown in a greenhouse as described previously[S1]. *Arabidopsis thaliana* plants were grown at 22 °C under a photoperiod of 16 h white light (50 μmol m^{-2} s^{-1}) / 8 h dark.

Chemical library screening

We screened a total of 22 compounds from the homemade sulfonimide compound collection. Compounds were dissolved in dimethyl sulfoxide (DMSO) at a concentration of 10 mM. Each compound was added at a 1:200 dilution to basal buffer (5 mM MES/bistrispropane [pH 6.5], 50 mM KCl, and 0.1 mM $CaCl_2$) for a final concentration of 50 μM. Plants were first incubated in the dark overnight to ensure complete closure of stomata prior to assay. Using such plants, under dim light, 1–2 cm^2 epidermal peels were excised from fully expanded leaves of 4- to 6-week-old plants using scissors and forceps, respectively. The epidermal peels were immersed in the buffer containing chemical compounds and were incubated under fluorescent white light (50 μmol m^{-2} s^{-1}) or in the dark at 25°C for 4 h, and samples in which the stomata were uniformly closed were identified using a stereoscopic microscope (Stereo Discovery; Zeiss, Oberkochen, Germany).

Measurement of stomatal apertures

Samples of *C. benghalensis* were prepared as described above, while epidermal fraction of using fully expanded rosette leaves from 5 to 7-week-old plants of *Arabidopsis thaliana* was prepared as described previously[S1]. For the light irradiation, 50 μmol m^{-2} s^{-1} red light and 10 μmol m^{-2} s^{-1} blue light were used instead of white light. After treatment of compounds in varying concentration, images were acquired using an optical microscope (BX43; Olympus, Tokyo, Japan) with a charge-coupled device (CCD) camera (DP27; Olympus) with a ×10 objective lens (UPlanFL N; Olympus). Stomatal apertures of respective stomata were measured using FLUOVIEW software (Olympus).

Quantification of the degree of H^+-ATPase phosphorylation

Blue light-induced phosphorylation of PM H+-ATPase in hypocotyls and guard cells and from the epidermis of *Arabidopsis* was determined by immunoblot or by immunofluorescence, as described previously[S2,3].

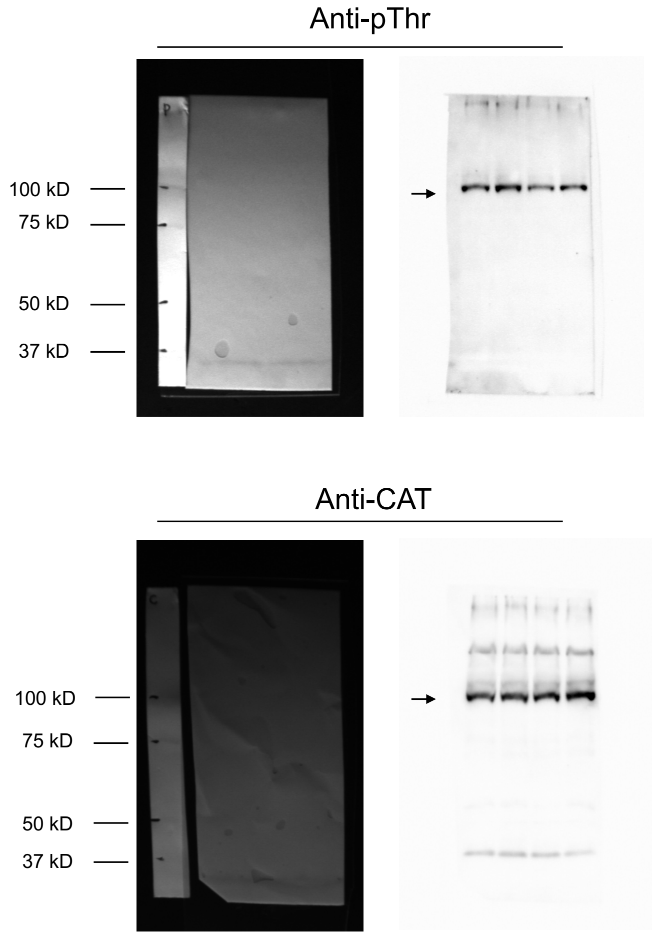

Figure S 1. Uncropped raw blot images of Anti-pThr and Anti-CAT used in Fig. 3C, respectively. The position of the molecular markers is drawn in the left. The arrows indicate position of H^+-ATPase which the surrounding area was respectively cropped and used in Figure 3C, top.

Evaluation of cellular esterase activity in guard cells of *C. benghalensis* visualized by fluorescent diacetate (FDA) staining

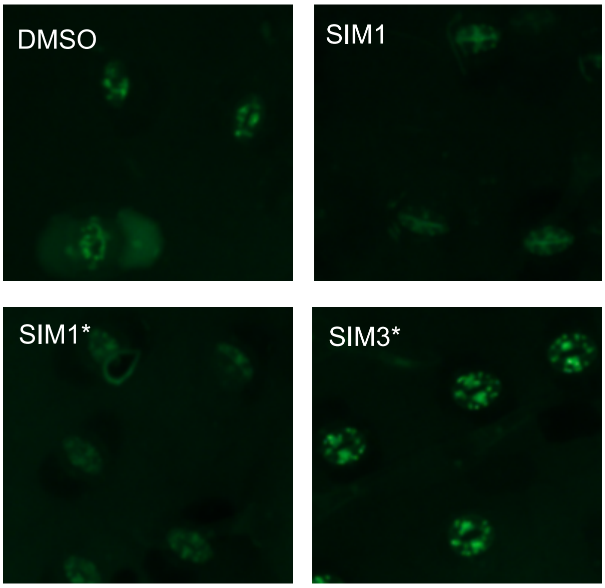

Figure S 2. The raw output images which correspond to Fig. 5B left are displayed.

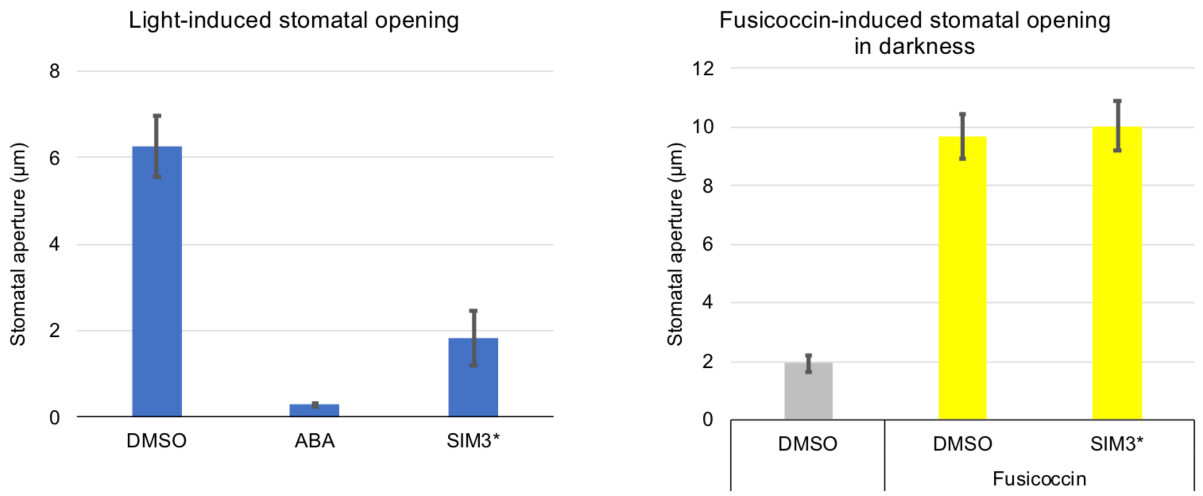

Figure S 3. The effect of SIM3* on stomatal opening induced by fungal toxin, fusicoccin. First, the epidermal peels of *C. benghalensis* were immersed in the buffer containing 50 µM compounds and incubated for 30 min. Samples were further incubated under light irradiation (left panel) or in the absence/presence of 10 µM fusicoccin in darkness (right panel) for 3 h prior to stomatal aperture quantification. Means ± SD (*n*=3; 56-94 stomata were measured per replicate).

Experimental procedures for studies on organic chemistry

The SIM1 derivatives were prepared following reported experimental procedures.

Procedure for the synthesis of 2,4-diaryloxazole.[S4]

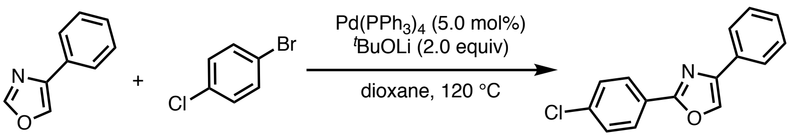

(2.0 equiv)

oxa1
84%

The synthesis of **oxa1** is representative: To a Schlenk tube a magnetic stirring bar, 1-Bromo-4-chlorobenzene (382 mg, 2.0 mmol, 2.0 equiv), *t*-BuOLi (160 mg, 2.0 mmol, 2.0 equiv) and $Pd(PPh_3)_4$ (58 mg, 0.050 mmol, 5.0 mol%) were added. The tube was filled with nitrogen by employing a usual Schlenk technique (evacuate-refill cycle). 1,4-Dioxane (3.0 mL) and 4-phenyloxazole (145 mg, 1.0 mmol) were added to the tube and the mixture was heated at 120 °C for 3 h. The mixture was then cooled to 25 °C. The crude solution was diluted with water (30 mL) and EtOAc (15 mL) and then extracted with EtOAc (3 × 25 mL). The combined organic layer was washed with brine and dried over anhydrous Na_2SO_4. The solvent was concentrated *in vacuo*. Purification by chromatography on silica-gel (*n*-hexane/EtOAc = 50:1 to 25:1) provided **oxa1** (215 mg, 0.84 mmol, 84%).

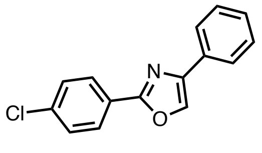

oxa1
84%

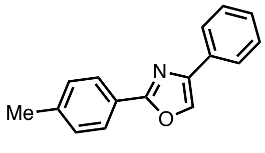

oxa2
91%[a]

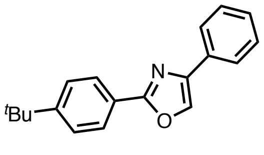

oxa3
43%

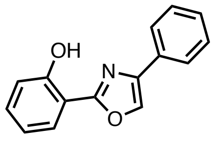

oxa4
14%

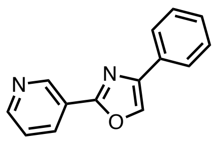

oxa5
66%

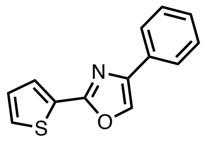

oxa6
74%

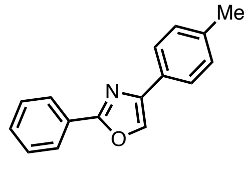

oxa7
54%

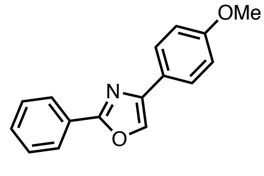

oxa8
67%

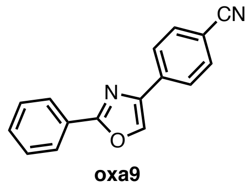

oxa9
96%

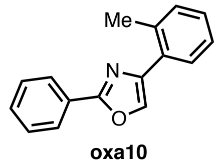

oxa10
81%

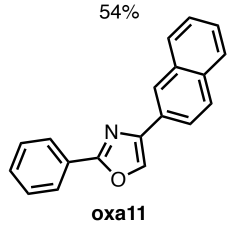

oxa11
88%

(a) ArBr (2.0 equiv), $Pd(dba)_3 \cdot CHCl_3$ (2.5 mol%), P(*p*-tol)$_3$ (10 mol%), ^{t}BuOLi (2.0 equiv), dioxane, 120 °C

Procedure for the synthesis of 2,4-diaryloxazoles with Ag salt.[S5]

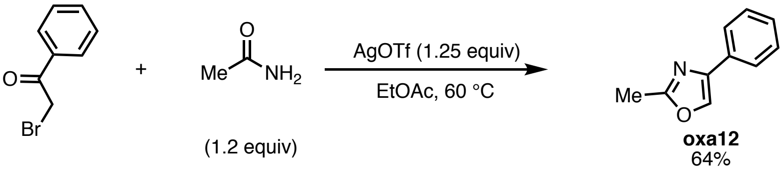

The synthesis of **oxa12** is representative: 2-Bromo-1-phenylethan-1-one (597 mg, 3.0 mmol), acetamide (215 mg, 3.6 mmol, 1.2 equiv) and silver triflate (964 mg, 3.8 mmol, 1.3 equiv) were added to a Schlenk tube in the open air. The tube was filled with nitrogen by employing a usual Schlenk technique (evacuate-refill cycle). Ethyl acetate (4.0 mL) was added to the tube and the mixture was heated at 50 °C for 6 h in the closed system. The mixture was then cooled to 25 °C and diluted with ethyl acetate (3.0 mL). A solution of saturated NaCl (4.0 mL) was added and the mixture stirred for 15 h. The insoluble residue was removed by filtration and washed with ethyl acetate (20 mL). The resulting biphasic solution was transferred to a separatory funnel and the layers separated. The organic layer was washed with water (10 mL), 5% $NaHCO_3$ (10 mL), 1 M HCl (10 mL), and water (10 mL). The combined organic layer was washed with brine and dried over anhydrous Na_2SO_4. The solvent was concentrated *in vacuo*. Purification by chromatography on silica-gel (*n*-hexane/EtOAc = 50:1) provided **oxa12** (307 mg, 1.93 mmol, 64%). The synthesis of **thia1** was according to the literature.[S6]

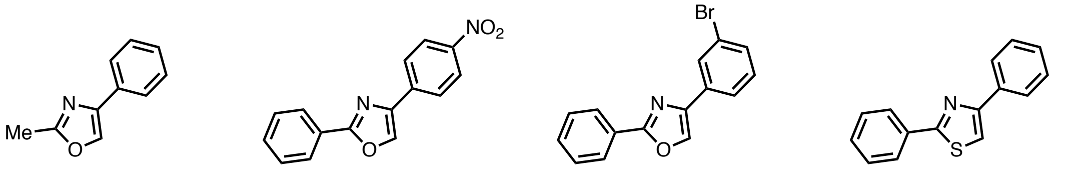

oxa12 64% | **oxa13** 45% | **oxa14** 48% | **thia1** 99%[a]

(a) AgOTf and EtOAc were not added. Glycerin was used as solvent, rt, 1h.

Procedure for the synthesis of SIM1 derivatives through Cu-catalyzed C–H imidation.[S7]

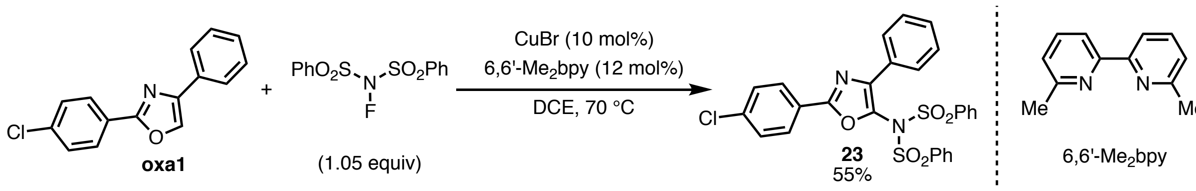

The synthesis of **23** is representative: 2-(4-Chlorophenyl)-4-phenyloxazole **oxa1** (51 mg, 0.20 mmol), NFSI (66 mg, 0.21 mmol, 1.05 equiv), CuBr (2.9 mg, 0.020 mmol, 10 mol%) and 6,6'-Me_2bpy (4.4 mg, 0.024 mmol, 12 mol%) were added to a Schlenk tube in the open air. The tube was filled with nitrogen by employing a usual Schlenk technique (evacuate-refill cycle). 1,2-Dichloroethane (1.0 mL) was added to the tube and the mixture was heated at 70 °C for 12 h in the closed system. The mixture was then cooled to 25 °C. The crude solution was filtered through a pad of silica gel (ca. 6 g) and Na_2SO_4 (ca. 20 g) in a column and concentrated *in vacuo*. Purification by chromatography on silica-gel (*n*-hexane/EtOAc = 5:1 to 4:1) followed by recrystallization from CH_2Cl_2/MeOH provided **23** (61 mg, 0.11 mmol, 55%).

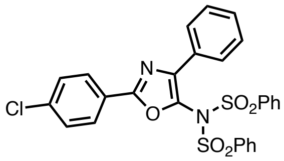

23
55%

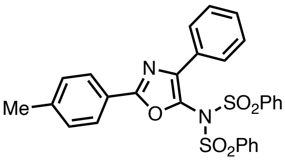

24
67%

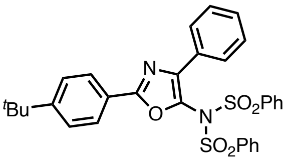

25
64%

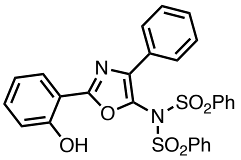

26
39%

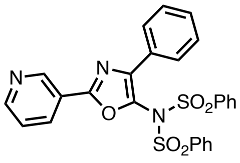

27
50%

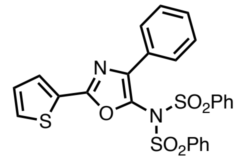

28
56%[S10]

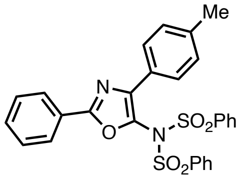

31
61%

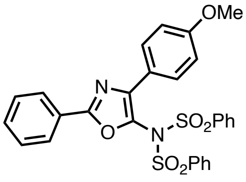

32
26%

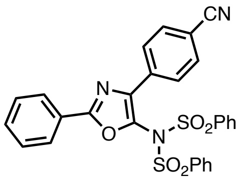

33
85%

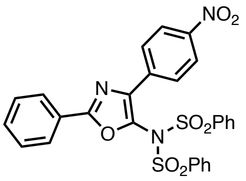

34
82%

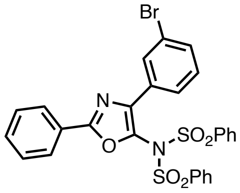

35
76%

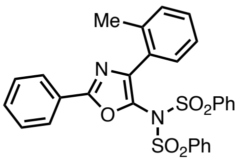

36
79%

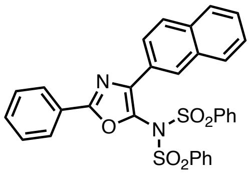

37
66%

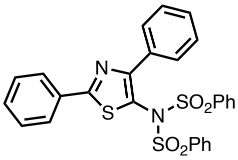

39
22%

Procedure for the synthesis of 38.[S8]

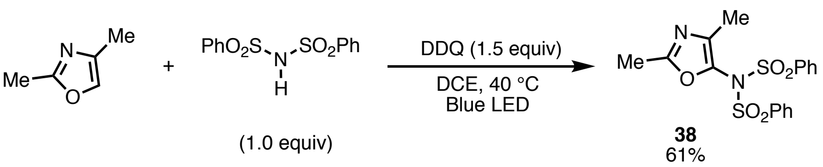

(1.0 equiv)

38
61%

Synthesis of **38** is representative. 2,4-Dimethyloxazole (19 mg, 0.20 mmol), *N*-(phenylsulfonyl)benzenesulfonamide (59 mg, 0.20 mmol, 1.0 equiv) and DDQ (68 mg, 0.30

mmol, 1.5 equiv) were added to a test tube in the open air. 1,2-Dichloroethane (2.0 mL) was added to the tube and the mixture was stirred with shedding blue light for 12 h at room temperature under air. The mixture was shielded with aluminum foil. The crude solution was filtered through a pad of silica gel and Na_2SO_4 in a column and concentrated *in vacuo*. Purification by chromatography on silica-gel (*n*-hexane 100% to *n*-hexane/EtOAc = 3:2) provided **38** (48 mg, 0.12 mmol, 61%).

Procedure for the synthesis of SIM1 derivatives through photoredox-catalyzed C–H imidation.[S9]

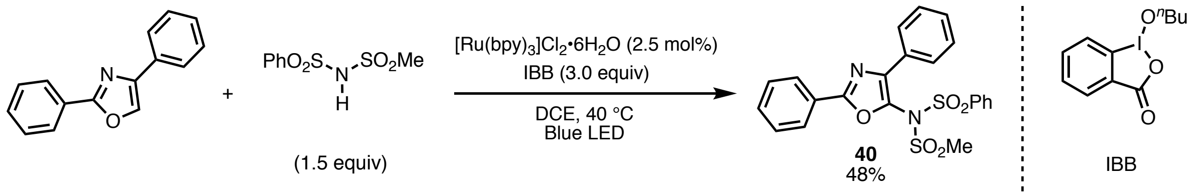

The synthesis of **40** is representative: 2,4-Diphenyloxazole (44 mg, 0.20 mmol), *N*-(methylsulfonyl)benzenesulfonamide (71 mg, 0.30 mmol, 1.5 equiv), IBB (192 mg, 0.60 mmol, 3.0 equiv), and $[Ru(bpy)_3]Cl_2 \cdot 6H_2O$ (3.7 mg, 0.005 mmol, 2.5 mol%) were added to a Schlenk tube in the open air. The tube was filled with nitrogen by employing a usual Schlenk technique (evacuate-refill cycle). 1,2-Dichloroethane (2.0 mL) was added to the tube and the mixture was stirred with shedding blue light (three 2.88 W blue LED strips were located 3 cm away from the reaction vials) for 24 h in the closed system. The reaction temperature was 25 °C at the beginning and gradually increased to 40 °C by irradiation of blue light. The crude solution was diluted with EtOAc (10 mL), then 1 M NaOH (10 mL) was added. The organic phase was extracted using EtOAc. The combined organic layers were washed with brine and dried over anhydrous Na_2SO_4. The solvent was concentrated *in vacuo*. Purification by chromatography on silica-gel (*n*-hexane/EtOAc = 5:1 to 4:1) followed by recrystallization from CH_2Cl_2/MeOH provided **40** (43 mg, 0.095 mmol, 48%).

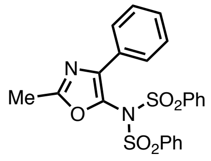

29
64%

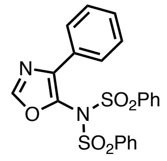

30
44%[a]

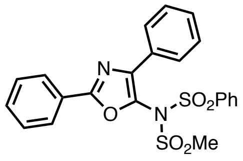

40
48%

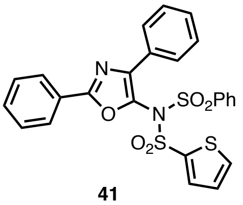

41
35%

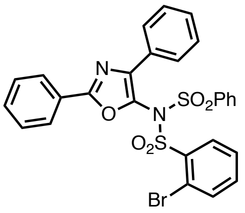

42
37%

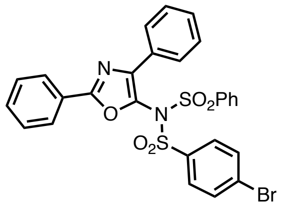

43
29%

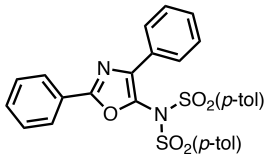

44
56%

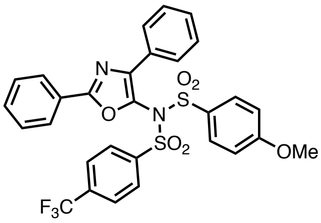

45
46%

(a) sulfonimide reagent (1.0 equiv),$[Ru(bpy)_3]Cl_2•6H_2O$ (2.5 mol%), IBB (2.0 equiv), 12 h

Procedure for the synthesis of 46 and 47.[S9]

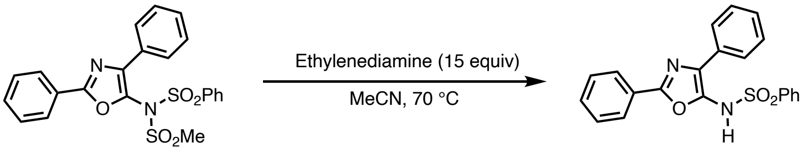

40 **46** 84%

The synthesis of **46** is representative: **40** (90 mg, 0.20 mmol) was added to a Schlenk tube in the open air. Acetonitrile (2.0 mL) and 1,2-ethylenediamine (200 μL, 3.0 mmol, 15 equiv) were added to the tube and the mixture was heated at 70 °C for 20 min. The mixture was then cooled to 25 °C. The crude solution was diluted with CH_2Cl_2 (10 mL) and then saturated NH_4Cl aq. (10 mL). The aqueous layer was washed with CH_2Cl_2 (×3), then the combined organic layers were washed with brine and dried over anhydrous Na_2SO_4. The solvent was concentrated *in vacuo* and provided crude solids. To the solids, aq. NaOH (1 M) was added. The aqueous layer was washed with CH_2Cl_2. The aqueous layer was then acidified with aq. HCl (1 M) and then extracted with EtOAc three times. The organic layer was dried over anhydrous Na_2SO_4. The solvent was concentrated *in vacuo* and provided **46** (63 mg, 0.17 mmol, 84%).

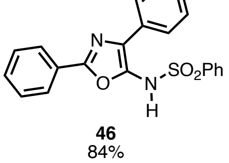

46
84%

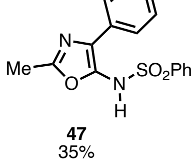

47
35%

Characterization Data

Compounds **1–20**[S7], **28**[S10], **30**[S9], **38**[S8], **oxa2**[S11], **oxa5**[S12], **oxa6**[S10], **oxa7**[S11], **oxa8**[S11], **oxa10**[S11], **oxa12**[S13], **oxa13**[S14], and **thia1**[S15] are known compounds and showed the identical spectra according to the literature. Compounds **1-20**[S7], **28**[S10], **30**[S9], and **38**[S8] were synthesized in our previous paper.

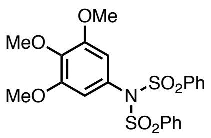

***N*-(phenylsulfonyl)-*N*-(3,4,5-trimethoxyphenyl)benzenesulfonamide (21)**: ^{1}H NMR ($CDCl_3$) δ 3.66 (s, 6H), 3.87 (s, 3H), 6.16 (s, 2H), 7.57 (app t, *J* = 7.8 Hz, 4H), 7.69 (t, *J* = 7.8 Hz, 2H), 8.00 (d, *J* = 7.8 Hz, 4H); ^{13}C NMR ($CDCl_3$) δ 56.30, 61.15, 109.15, 128.91, 129.16, 129.40, 134.21, 139.60, 139.90, 153.32; HRMS (ESI-MS, positive): *m/z* = 486.0645. calcd for $C_{21}H_{21}NO_7S_2Na$: 486.0652 $[M + Na]^+$. (This compound was synthesized following the general procedure for the Cu-catalyzed C–H imidation.[S7])

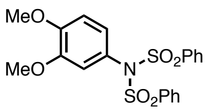

***N*-(3,4-dimethoxyphenyl)-*N*-(phenylsulfonyl)benzenesulfonamide (22):** ^{1}H NMR ($CDCl_3$) δ 3.66 (s, 3H), 3.90 (s, 3H), 6.38 (d, *J* = 1.8 Hz, 1H), 6.63 (dd, *J* = 9.0 Hz, 1.8 Hz, 1H), 6.80 (d, *J* = 9.0 Hz, 1H), 7.56 (app t, *J* = 7.8 Hz, 4H), 7.68 (t, *J* = 7.8 Hz, 2H), 7.97 (d, *J* = 7.8 Hz, 4H); ^{13}C NMR ($CDCl_3$) δ 56.09, 56.16, 110.90, 114.53, 124.55, 126.58, 128.83, 129.15, 134.10, 139.69, 149.15, 150.80; HRMS (ESI-MS, positive): *m/z* =456.0542. calcd for $C_{20}H_{19}NO_6S_2Na$: 456.0546 $[M + Na]^+$. (This compound was synthesized following the general procedure for the Cu-catalyzed C–H imidation.[7])

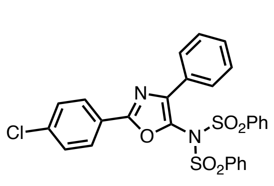

***N*-[2-(4-chlorophenyl)-4-phenyloxazol-5-yl]-*N*-(phenylsulfonyl)benzenesulfonamide (23):** ^{1}H NMR ($CDCl_3$) δ 7.18 (t, *J* = 7.2 Hz, 2H), 7.23 (t, *J* = 7.2 Hz, 1H), 7.44-7.47 (m, 6H), 7.62

(t, J = 7.8 Hz, 2H), 7.69 (d, J = 7.2 Hz, 2H), 7.92 (d, J = 7.8 Hz, 2H), 7.96 (d, J = 7.8 Hz, 4H); ^{13}C NMR ($CDCl_3$) δ 125.47, 126.98, 128.15, 128.62, 129.11, 129.16, 129.23, 129.43, 133.45, 134.74, 137.71, 139.25, 141.02, 159.81 (one sp^2 peak was not observed because of overlapping.); HR-MS (ESI-MS, positive): *m/z* = 573.0317. calcd for $C_{27}H_{19}ClN_2O_5S_2Na$: 573.0316 [M + Na]$^+$.

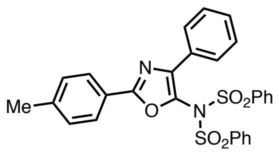

***N*-[4-phenyl-2-(*p*-tolyl)oxazol-5-yl]-*N*-(phenylsulfonyl)benzenesulfonamide (24):** ^{1}H NMR ($CDCl_3$) δ 2.43 (s, 3H), 7.17 (t, J = 7.2 Hz, 2H), 7.22 (t, J = 7.2 Hz, 1H), 7.28 (d, J = 7.8 Hz, 2H), 7.45 (app t, J = 7.8 Hz, 4H), 7.61 (t, J = 7.8 Hz, 2H), 7.70 (d, J = 7.2 Hz, 2H), 7.87 (d, J = 7.8 Hz, 2H), 7.97 (d, J = 7.8 Hz, 4H); ^{13}C NMR ($CDCl_3$) δ 21.83, 124.31, 126.86, 127.00, 128.56, 129.05, 129.12, 129.49, 129.76, 132.89, 134.63, 139.34, 140.84, 141.93, 160.99 (one sp^2 peak was not observed because of overlapping.); HR-MS (ESI-MS, positive): *m/z* = 531.1048. calcd for $C_{28}H_{23}N_2O_5S_2$: 531.1043[M + H]$^+$.

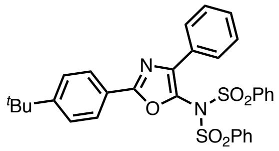

***N*-{2-[4-(*tert*-butyl)phenyl]-4-phenyloxazol-5-yl}-*N*-(phenylsulfonyl)benzenesulfonamide (25):** ^{1}H NMR ($CDCl_3$) δ 1.37 (s, 9H), 7.17 (t, J = 7.2 Hz, 2H), 7.22 (t, J = 7.2 Hz, 1H), 7.45 (app t, J = 7.8 Hz, 4H), 7.50 (d, J = 8.4 Hz, 2H), 7.61 (t, J = 7.8 Hz, 2H), 7.70 (d, J = 7.2 Hz, 2H), 7.92 (d, J = 8.4 Hz, 2H), 7.96 (d, J = 7.8 Hz, 4H); ^{13}C NMR ($CDCl_3$) δ 31.36, 35.23, 124.25, 126.01, 126.72, 127.01, 128.55, 129.04, 129.11, 129.50, 132.92, 134.62, 139.33, 140.86, 155.03, 160.91 (one sp^2 peak was not observed because of overlapping.); HR-MS (ESI-MS, positive): *m/z* = 595.1332. calcd for $C_{31}H_{28}N_2O_5S_2Na$: 595.1332 [M + Na]$^+$.

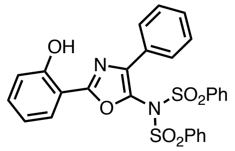

***N*-[2-(2-hydroxyphenyl)-4-phenyloxazol-5-yl]-*N*-(phenylsulfonyl)benzenesulfonamide (26):** ^{1}H NMR ($CDCl_3$) δ 6.95 (t, J = 7.8 Hz, 1H), 7.11 (d, J = 7.8 Hz, 1H), 7.19 (t, J = 7.2 Hz, 2H), 7.26 (m, 1H), 7.42 (t, J = 7.8 Hz, 1H), 7.46 (t, J = 7.8 Hz, 4H), 7.57 (dd, J = 7.8, 1.8 Hz,

1H), 7.61–7.65 (m, 4H), 7.97 (d, *J* = 7.8 Hz, 4H), 10.99 (s, 1H); ^{13}C NMR ($CDCl_3$) δ 110.39, 117.83, 119.87, 126.49, 126.90, 128.20, 128.73, 129.12, 129.21, 129.56, 132.67, 133.69, 134.82, 138.91, 139.15, 158.38, 160.87; HR-MS (ESI-MS, positive): *m/z* = 555.0656. calcd for $C_{27}H_{20}N_2O_6S_2Na$: 555.0657 [*M* + Na]$^+$.

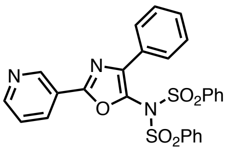

***N*-[4-phenyl-2-(pyridin-3-yl)oxazol-5-yl]-*N*-(phenylsulfonyl)benzenesulfonamide (27):** ^{1}H NMR ($CDCl_3$) δ 7.19 (t, *J* = 7.2 Hz, 2H), 7.24 (t, *J* = 7.2 Hz, 1H), 7.43–7.48 (m, 5H), 7.63 (t, *J* = 7.8 Hz, 2H), 7.71 (d, *J* = 7.2 Hz, 2H), 7.97 (d, *J* = 7.8 Hz, 4H), 8.30 (dt, *J* = 7.2 Hz, 1.8 Hz, 1H), 8.75 (dd, *J* = 5.4 Hz, 1.8 Hz, 1H), 9.16 (s, 1H); ^{13}C NMR ($CDCl_3$) δ 123.29, 123.85, 127.01, 128.67, 128.99, 129.11, 129.22, 129.37, 134.01, 134.09, 134.83, 139.18, 141.06, 152.06, 158.50 (one sp^2 peak was not observed because of overlapping.); HR-MS (ESI-MS, positive): *m/z* = 540.0660. calcd for $C_{26}H_{19}N_3O_5S_2Na$: 540.0658 [*M* + Na]$^+$.

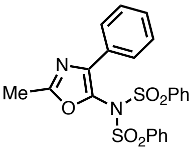

***N*-(2-methyl-4-phenyloxazol-5-yl)-*N*-(phenylsulfonyl)benzenesulfonamide (29):** ^{1}H NMR ($CDCl_3$) δ 2.52 (s, 3H), 7.12 (t, *J* = 7.8 Hz, 2H), 7.17 (t, *J* = 7.8 Hz, 1H), 7.42 (app t, *J* = 7.8 Hz, 4H), 7.55 (d, *J* = 7.8 Hz, 2H), 7.58 (t, *J* = 7.8 Hz, 2H), 7.94 (d, *J* = 7.8 Hz, 4H); ^{13}C NMR ($CDCl_3$) δ 14.85, 126.72, 128.52, 128.92, 128.97, 129.11, 129.26, 132.97, 134.62, 139.25, 139.74, 161.56; HR-MS (ESI-MS, positive): *m/z* = 477.0548. calcd for $C_{22}H_{18}N_2O_5S_2Na$: 477.0549 [*M* + Na]$^+$.

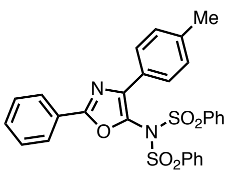

***N*-[2-phenyl-4-(*p*-tolyl)oxazol-5-yl]-*N*-(phenylsulfonyl)benzenesulfonamide (31):** ^{1}H NMR ($CDCl_3$) δ 2.31 (s, 3H), 6.97 (d, *J* = 8.4 Hz, 2H), 7.43–7.50 (m, 7H), 7.58–7.62 (m, 4H), 7.96–7.98 (m, 6H); ^{13}C NMR ($CDCl_3$) δ 21.48, 126.52, 126.86, 126.90, 127.06, 128.90, 129.01,

129.11, 129.27, 131.37, 132.79, 134.47, 139.01, 139.36, 141.05, 160.64; HR-MS (ESI-MS, positive): *m/z* = 553.0861. calcd for $C_{28}H_{22}N_2O_5S_2Na$: 553.0862 [*M* + Na]$^+$.

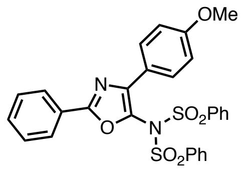

N-(4-(4-methoxyphenyl)-2-phenyloxazol-5-yl)-N-(phenylsulfonyl)benzenesulfonamide (32): ^{1}H NMR ($CDCl_3$) δ 3.80 (s, 3H), 6.70 (d, *J* = 9.0 Hz, 2H), 7.45–7.49 (m, 7H), 7.62 (t, *J* = 7.8 Hz, 2H), 7.65 (d, *J* = 9.0 Hz, 2H), 7.97–7.98 (m, 6H); ^{13}C NMR ($CDCl_3$) δ 55.48, 114.05, 122.00, 126.86, 127.08, 128.44, 129.01, 129.13, 131.36, 132.25, 134.55, 139.43, 140.83, 160.28, 160.59 (one sp^2 peak was not observed because of overlapping.); HR-MS (ESI-MS, positive): *m/z* = 569.0812 calcd for $C_{28}H_{22}N_2O_6S_2Na$: 569.0811 [*M* + Na]$^+$.

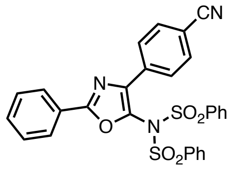

N-[4-(4-cyanophenyl)-2-phenyloxazol-5-yl]-N-(phenylsulfonyl)benzenesulfonamide (33): ^{1}H NMR ($CDCl_3$) δ 7.44 (d, *J* = 8.4 Hz, 2H), 7.47–7.54 (m, 7H), 7.66 (t, *J* = 7.8 Hz, 2H), 7.80 (d, *J* = 8.4 Hz, 2H), 7.97–7.99 (m, 6H); ^{13}C NMR ($CDCl_3$) δ 112.36, 118.70, 126.50, 126.94, 127.31, 129.07, 129.16, 129.32, 131.86, 132.22, 133.80, 134.64, 134.94, 138.93, 139.04, 161.20; HR-MS (ESI-MS, positive): *m/z* = 564.0658. calcd for $C_{28}H_{19}N_3O_5S_2Na$: 564.0658 [*M* + Na]$^+$.

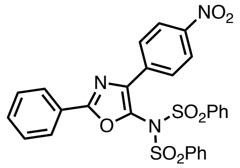

N-[4-(4-nitrophenyl)-2-phenyloxazol-5-yl]-N-(phenylsulfonyl)benzenesulfonamide (34): ^{1}H NMR ($CDCl_3$) δ 7.48–7.53 (m, 6H), 7.56 (t, *J* = 7.2 Hz, 1H), 7.65 (t, *J* = 7.8 Hz, 2H), 7.85 (d, *J* = 7.8 Hz, 2H), 7.98–8.02 (m, 8H); ^{13}C NMR ($CDCl_3$) δ 123.71, 126.46, 127.00, 127.55, 129.10, 129.21, 129.38, 131.96, 134.96, 134.99, 135.60, 138.64, 139.02, 147.78, 161.30; HR-MS (ESI-MS, positive): *m/z* = 584.0551. calcd for $C_{27}H_{19}N_3O_7S_2Na$: 584.0557 [*M* + Na]$^+$.

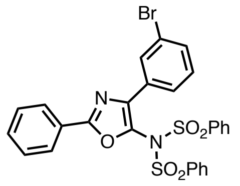

***N*-[4-(3-bromophenyl)-2-phenyloxazol-5-yl]-*N*-(phenylsulfonyl)benzenesulfonamide (35):** ^{1}H NMR ($CDCl_3$) δ 7.02 (t, *J* = 7.8 Hz, 1H), 7.34 (dd, *J* = 7.2 Hz, 1.8 Hz, 1H), 7.47–7.53 (m, 7H), 7.60–7.65 (m, 3H), 7.84 (t, *J* = 1.8 Hz, 1H), 7.98–8.01 (m, 6H); ^{13}C NMR ($CDCl_3$) δ 122.76, 125.44, 126.76, 126.94, 129.01, 129.11, 129.27, 129.89, 130.05, 131.43, 131.65, 132.09, 133.72, 134.83, 139.13, 139.49, 160.96; HR-MS (ESI-MS, positive): *m/z* = 616.9825. calcd for $C_{27}H_{19}BrN_2O_5S_2Na$: 616.9811 [*M* + Na]$^+$.

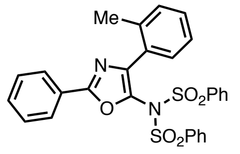

***N*-[2-phenyl-4-(*o*-tolyl)oxazol-5-yl]-*N*-(phenylsulfonyl)benzenesulfonamide (36):** ^{1}H NMR ($CDCl_3$) δ 2.39 (s, 3H), 7.04 (t, *J* = 7.8 Hz, 1H), 7.17 (d, *J* = 7.8 Hz, 1H), 7.22 (t, *J* = 7.8 Hz, 1H), 7.41 (t, *J* = 7.8 Hz, 4H), 7.45–7.53 (m, 4H), 7.59 (t, *J* = 7.8 Hz, 2H), 7.87 (d, *J* = 7.8 Hz, 4H), 7.95 (d, *J* = 7.8 Hz, 2H); ^{13}C NMR ($CDCl_3$) δ 20.56, 126.06, 126.76, 127.09, 128.87, 129.00, 129.05, 129.52, 129.67, 130.81, 131.37, 134.43, 134.69, 137.94, 139.11, 142.17, 160.13 (one sp^2 peak was not observed because of overlapping.); HR-MS (ESI-MS, positive): *m/z* = 553.0865. calcd for $C_{28}H_{22}N_2O_5S_2Na$: 553.0862 [*M* + Na]$^+$.

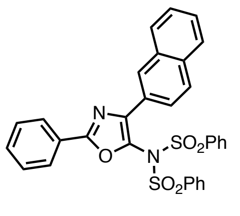

***N*-[4-(naphthalen-2-yl)-2-phenyloxazol-5-yl]-*N*-(phenylsulfonyl)benzenesulfonamide (37):** ^{1}H NMR ($CDCl_3$) δ 7.30–7.33 (m, 4H), 7.41–7.53 (m, 7H), 7.62 (d, *J* = 8.4 Hz, 1H), 7.69 (d, *J* = 8.4 Hz, 1H), 7.75 (d, *J* = 7.2 Hz, 1H), 7.78 (dd, *J* = 8.4 Hz, 1.2 Hz, 1H), 7.97 (d, *J* = 7.8 Hz, 4H), 8.04 (d, *J* = 7.2 Hz, 2H), 8.22 (s, 1H); ^{13}C NMR ($CDCl_3$) δ 124.17, 126.22, 126.72, 126.83, 126.93, 126.98, 127.59, 128.22, 128.89, 129.00, 129.07, 131.50, 133.15, 133.59, 134.45, 139.28, 140.92, 160.87 (three sp^2 peaks were not observed because of overlapping.); HR-MS (ESI-MS, positive): *m/z* = 589.0863. calcd for $C_{31}H_{22}N_2O_5S_2Na$: 589.0862 [*M* + Na]$^+$.

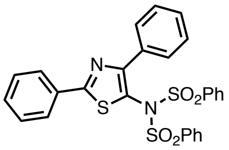

***N*-(2,4-diphenylthiazol-5-yl)-*N*-(phenylsulfonyl)benzenesulfonamide (39):** ^{1}H NMR ($CDCl_3$) δ 7.18 (t, *J* = 7.2 Hz, 2H), 7.24 (t, *J* = 7.2 Hz, 1H), 7.42 (t, *J* = 7.8 Hz, 4H), 7.44-7.47 (m, 3H), 7.59 (t, *J* = 7.8 Hz, 2H), 7.75 (d, *J* = 7.2 Hz, 2H), 7.90 (d, *J* = 7.8 Hz, 4H), 7.96 (d, *J* = 7.2 Hz, 2H); ^{13}C NMR ($CDCl_3$) δ 124.16, 126.85, 128.45, 128.66, 129.04, 129.09, 129.16, 129.39, 131.14, 132.72, 133.48, 134.61, 138.57, 156.69, 167.92; HR-MS (ESI-MS, positive): *m/z* = 555.0477. calcd for $C_{27}H_{20}N_2O_4S_3Na$: 555.0477 [*M* + Na]$^+$.

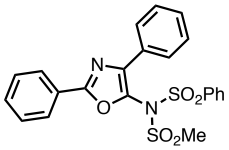

***N*-(2,4-diphenyloxazol-5-yl)-*N*-(methylsulfonyl)benzenesulfonamide (40):** ^{1}H NMR ($CDCl_3$) δ 3.51 (s, 3H), 7.25–7.29 (m, 3H), 7.37 (app t, *J* = 7.8 Hz, 2H), 7.45–7.51 (m, 3H), 7.56 (t, *J* = 7.8 Hz, 1H), 7.76 (dd, *J* = 7.8 Hz, 2.4 Hz, 2H), 7.86 (d, *J* = 7.8 Hz, 2H), 8.06 (d, *J* = 7.8 Hz, 2H); ^{13}C NMR ($CDCl_3$) δ 44.78, 126.92, 126.98, 127.13, 128.80, 129.05, 129.11, 129.13, 129.35, 129.43, 131.54, 132.91, 134.83, 136.66, 140.67, 160.82; HR-MS (ESI-MS, positive): *m/z* = 477.0547 calcd for $C_{22}H_{18}N_2O_5S_2Na$: 477.0549 [*M* + Na]$^+$.

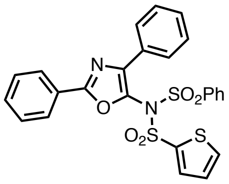

***N*-(2,4-diphenyloxazol-5-yl)-*N*-(phenylsulfonyl)thiophene-2-sulfonamide (41):** ^{1}H NMR ($CDCl_3$) δ 7.05 (t, *J* = 4.8 Hz ,1H), 7.20–7.25 (m, 3H), 7.44–7.53 (m, 5H), 7.61 (t, *J* = 7.8 Hz, 1H), 7.69 (d, *J* = 4.8 Hz, 1H), 7.72 (d, *J* = 7.2 Hz, 2H), 7.81 (d, *J* = 4.8 Hz, 1H), 7.96 (d, *J* = 7.8 Hz, 2H), 8.01 (d, *J* = 7.8 Hz, 2H); ^{13}C NMR ($CDCl_3$) δ 126.93, 127.00, 127.76, 128.61, 129.05, 129.13, 129.16, 129.47, 131.46, 133.03, 134.74, 135.58, 136.58, 138.99, 139.20, 140.94, 160.83 (two sp^2 peaks were not observed because of overlapping); HR-MS (ESI-MS, positive): *m/z* = 545.0275. calcd for $C_{25}H_{18}N_2O_5S_3Na$: 545.0270 [*M* + Na]$^+$.

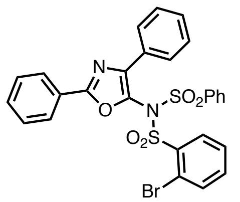

2-bromo-*N*-(2,4-diphenyloxazol-5-yl)-*N*-(phenylsulfonyl)benzenesulfonamide (42): ^{1}H NMR ($CDCl_3$) δ 7.20–7.25 (m, 3H), 7.37 (t, *J* = 7.8 Hz, 1H), 7.42 (t, *J* = 7.8 Hz, 2H), 7.46–7.50 (m, 4H), 7.58–7.60 (m, 2H), 7.77 (d, *J* = 7.8 Hz, 2H), 7.96 (d, *J* = 7.8 Hz, 2H), 8.02 (d, *J* = 7.8 Hz, 2H), 8.31 (d, *J* = 7.8 Hz, 1H); ^{13}C NMR ($CDCl_3$) δ 121.46, 127.04, 127.20, 127.33, 127.88, 128.48, 129.05, 129.16, 129.20, 129.45, 129.55, 131.44, 132.43, 133.57, 134.79, 135.18, 136.13, 138.82, 139.15, 141.90, 160.86; HR-MS (ESI-MS, positive): *m/z* = 616.9809. calcd for $C_{27}H_{19}BrN_2O_5S_2Na$: 616.9811 [*M* + Na]$^+$.

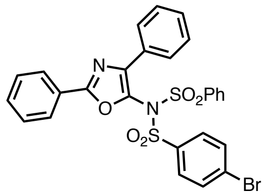

4-bromo-*N*-(2,4-diphenyloxazol-5-yl)-*N*-(phenylsulfonyl)benzenesulfonamide (43): ^{1}H NMR ($CDCl_3$) δ 7.19 (t, *J* = 7.8 Hz, 2H), 7.28 (t, *J* = 7.8 Hz, 1H), 7.48–7.52 (m, 7H), 7.64–7.66 (m, 3H), 7.76 (d, *J* = 8.4 Hz, 2H), 8.00–8.02 (m, 4H); ^{13}C NMR ($CDCl_3$) δ 126.91, 126.98, 128.66, 129.11, 129.15, 129.25, 130.31, 130.45, 131.57, 132.45, 133.00, 134.82, 138.03, 139.26, 141.06, 160.90 (three sp^2 peaks were not observed because of overlapping); HR-MS (ESI-MS, positive): *m/z* = 616.9813. calcd for $C_{27}H_{19}BrN_2O_5S_2Na$: 616.9811 [*M* + Na]$^+$.

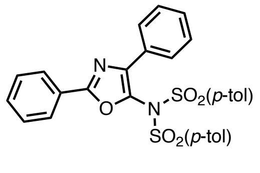

***N*-(2,4-diphenyloxazol-5-yl)-4-methyl-*N*-tosylbenzenesulfonamide (44):** ^{1}H NMR ($CDCl_3$) δ 2.42 (s, 6H), 7.16 (t, *J* = 7.2 Hz, 2H), 7.21–7.23 (m, 5H), 7.49–7.51 (m, 3H), 7.67 (d, *J* = 8.4 Hz, 2H), 7.84 (d, *J* = 8.4 Hz, 4H), 8.01 (d, *J* = 7.2 Hz, 2H); ^{13}C NMR ($CDCl_3$) δ 21.90, 126.90, 127.03, 127.10, 128.46, 128.61, 129.02, 129.11, 129.50, 129.75, 131.37, 133.42, 136.30, 140.78, 145.83, 160.66; HR-MS (ESI-MS, positive): *m/z* = 567.1015. calcd for $C_{29}H_{24}N_2O_5S_2Na$: 567.1019 [*M* + Na]$^+$.

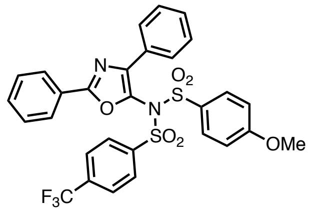

N-(2,4-diphenyloxazol-5-yl)-4-methoxy-N-[(4-trifluoromethylphenyl)sulfonyl]benzenesulfonamide (45): ^{1}H NMR ($CDCl_3$) δ 3.89 (s, 3H), 6.91 (d, J = 8.4 Hz, 2H), 7.17 (t, J = 7.2 Hz, 2H), 7.23 (t, J = 7.2 Hz, 1H), 7.50–7.54 (m, 3H), 7.63–7.66 (m, 4H), 7.92 (d, J = 8.4 Hz, 2H), 8.02 (dd, J = 7.8 Hz, 1.2 Hz, 2H), 8.07 (d, J = 8.4 Hz, 2H); ^{13}C NMR ($CDCl_3$) δ 55.98, 114.27, 114.48, 123.18 (q, J = 270 Hz), 126.19 (q, J = 4.0 Hz), 126.91, 126.98, 128.60, 129.11, 129.21, 129.34, 129.51, 130.16, 131.59, 131.62, 133.08, 135.89 (q, J = 32 Hz), 140.98, 142.82, 160.93, 164.81; HR-MS (ESI-MS, positive): m/z = 637.0682. calcd for $C_{29}H_{21}F_3N_2O_6S_2Na$: 637.0685 $[M + Na]^+$.

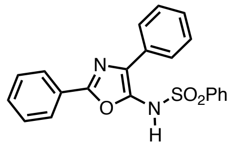

N-(2,4-diphenyloxazol-5-yl)benzenesulfonamide (46): ^{1}H NMR ($CDCl_3$) δ 6.58 (br s, 1H), 7.29–7.35 (m, 3H), 7.42–7.48 (m, 5H), 7.59 (t, J = 7.8 Hz, 1H), 7.85–7.90 (m, 6H); ^{13}C NMR ($CDCl_3$) δ 126.59, 126.84, 127.06, 127.82, 128.67, 128.75, 128.95, 129.31, 129.90, 131.08, 133.77, 134.94, 136.26, 139.81, 159.25; HR-MS (ESI-MS, positive): m/z = 377.0956. calcd for $C_{21}H_{17}N_2O_3S$: 377.0954 $[M + H]^+$.

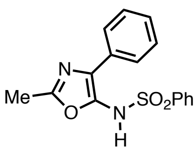

N-(2-methyl-4-phenyloxazol-5-yl)benzenesulfonamide (47): ^{1}H NMR (CD_2Cl_2) δ 2.35 (s, 3H), 7.24–7.31 (m, 3H), 7.45 (t, J = 7.8 Hz, 2H), 7.57 (tt, J = 7.5, 1.4 Hz, 1H), 7.68–7.71 (m, 2H), 7.78–7.81 (m, 2H); ^{13}C NMR (CD_2Cl_2) δ 14.46, 126.65, 127.73, 128.59, 128.72, 129.47, 130.27, 133.92, 134.88, 135.03, 139.96, 160.06; HR-MS (ESI-MS, positive): m/z =315.0797. calcd for $C_{16}H_{15}N_2O_3S$: 315.0798 $[M + H]^+$.

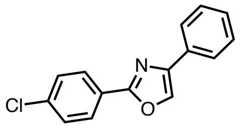

2-(4-chlorophenyl)-4-phenyloxazole (oxa1): ^{1}H NMR ($CDCl_3$) δ 7.34 (t, *J* = 7.2 Hz, 1H), 7.42–7.47 (m, 4H), 7.81 (d, *J* = 7.2 Hz, 2H), 7.97 (s, 1H), 8.06 (d, *J* = 8.4 Hz, 2H); ^{13}C NMR ($CDCl_3$) δ 125.83, 126.19, 128.00, 128.44, 128.98, 129.29, 131.13, 133.79, 136.71, 142.40, 161.22; HR-MS (ESI-MS, positive): *m/z* = 256.0524. calcd for $C_{15}H_{11}ClNO$: 256.0524 [*M* + H]$^{+}$.

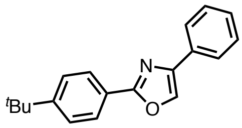

2-[4-(*tert*-butyl)phenyl]-4-phenyloxazole (oxa3): ^{1}H NMR ($CDCl_3$) δ 1.36 (s, 9H), 7.33 (t, *J* = 7.8 Hz, 1H), 7.43 (t, *J* = 7.8 Hz, 2H), 7.50 (d, *J* = 8.4 Hz, 2H), 7.82 (d, *J* = 7.8 Hz, 2H), 7.94 (s, 1H), 8.04 (d, *J* = 8.4 Hz, 2H); ^{13}C NMR ($CDCl_3$) δ 31.39, 35.13, 125.00, 125.85, 125.90, 126.53, 128.22, 128.91, 131.48, 133.36, 142.10, 153.97, 162.31; HR-MS (ESI-MS, positive): *m/z* = 300.1360. calcd for $C_{19}H_{19}NONa$: 300.1359 [*M* + Na]$^{+}$.

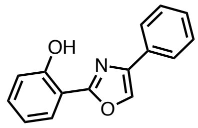

2-(4-phenyloxazol-2-yl)phenol (oxa4): ^{1}H NMR ($CDCl_3$) δ 6.98 (t, *J* = 7.8 Hz, 1H), 7.10 (d, *J* = 7.8 Hz, 1H), 7.34–7.39 (m, 2H), 7.44 (t, *J* = 7.8 Hz, 2H), 7.77 (d, *J* = 7.8 Hz, 2H), 7.87 (dd, *J* = 7.8 Hz, 1.2 Hz, 1H), 7.96 (s, 1H), 11.27 (s, 1H); ^{13}C NMR ($CDCl_3$) δ 111.21, 117.42, 119.66, 125.75, 126.24, 128.75, 129.05, 130.03, 132.32, 132.71, 140.45, 157.48, 161.81; HR-MS (ESI-MS, positive): *m/z* = 260.0681. calcd for $C_{15}H_{11}NO_2Na$: 260.0682 [*M* + Na]$^+$.

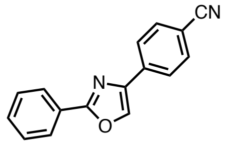

4-(2-phenyloxazol-4-yl)benzonitrile (oxa9): ^{1}H NMR ($CDCl_3$) δ 7.49–7.51 (m, 3H), 7.72 (d, *J* = 8.4 Hz, 2H), 7.94 (d, *J* = 8.4 Hz, 2H), 8.07 (s, 1H), 8.11–8.12 (m, 2H); ^{13}C NMR ($CDCl_3$) δ 111.63, 119.04, 126.24, 126.82, 127.21, 129.07, 131.04, 132.81, 135.22, 135.78, 140.70, 162.70; HR-MS (ESI-MS, positive): *m/z* = 269.0689. calcd for $C_{16}H_{10}N_2ONa$: 269.0685 [*M* + Na]$^+$.

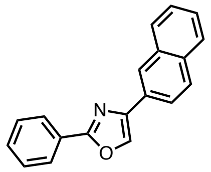

4-(naphthalen-2-yl)-2-phenyloxazole (oxa11): ^{1}H NMR ($CDCl_3$) δ 7.45–7.50 (m, 5H), 7.82–7.83 (m, 2H), 7.86 (d, *J* = 7.8 Hz, 1H), 7.90 (d, *J* = 7.8 Hz, 1H), 8.03 (s, 1H), 8.15 (dd, *J* = 7.8 Hz, 1.2 Hz, 2H), 8.38 (s, 1H); ^{13}C NMR ($CDCl_3$) δ 123.75, 124.65, 126.24, 126.58, 126.76, 127.68, 127.93, 128.45, 128.60, 128.62, 128.95, 130.63, 133.37, 133.79, 134.00, 142.24, 162.26; HR-MS (ESI-MS, positive): *m/z* = 272.1073. calcd for $C_{19}H_{14}NO$: 272.1070 [*M* + H]$^+$.

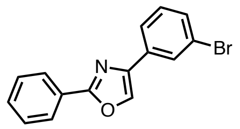

4-(3-bromophenyl)-2-phenyloxazole (oxa14): ^{1}H NMR ($CDCl_3$) δ 7.28 (t, J = 7.8 Hz, 1H), 7.44–7.49 (m, 4H), 7.72 (d, J = 7.2 Hz, 1H), 7.95 (s, 1H), 7.99 (t, J = 1.8 Hz, 1H), 8.10–8.11 (m, 2H); ^{13}C NMR ($CDCl_3$) δ 123.12, 124.32, 126.74, 127.46, 128.83, 128.98, 130.45. 130.76, 131.19, 133.40, 134.10, 140.91, 162.31; HR-MS (ESI-MS, positive): m/z = 300.0025. calcd for $C_{15}H_{11}BrNO$: 300.0019 $[M + H]^+$.

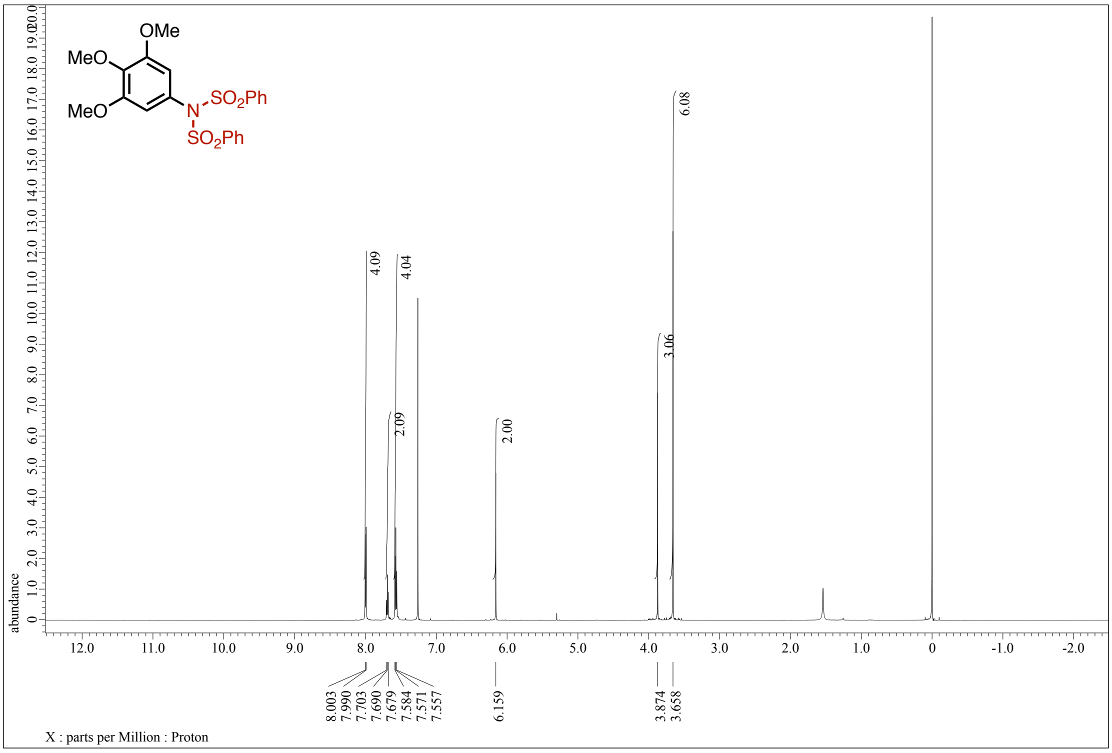

Figure S 4. ^{1}H NMR spectrum of **21.**

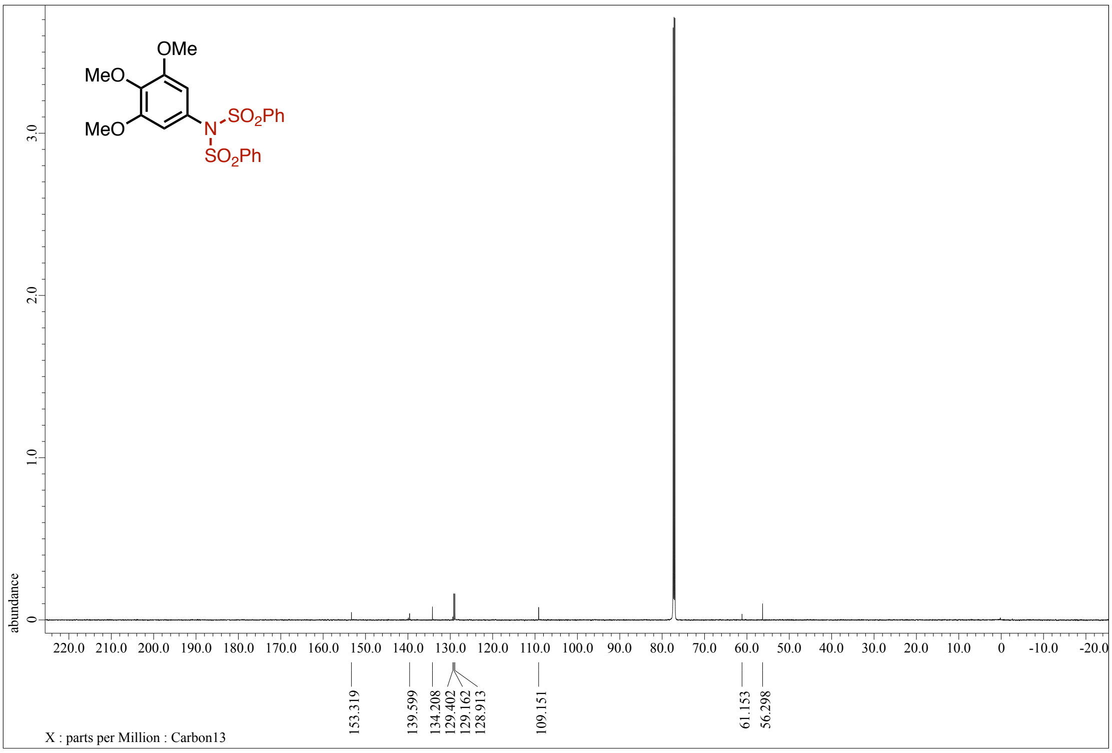

Figure S 5. ^{13}C NMR spectrum of **21**.

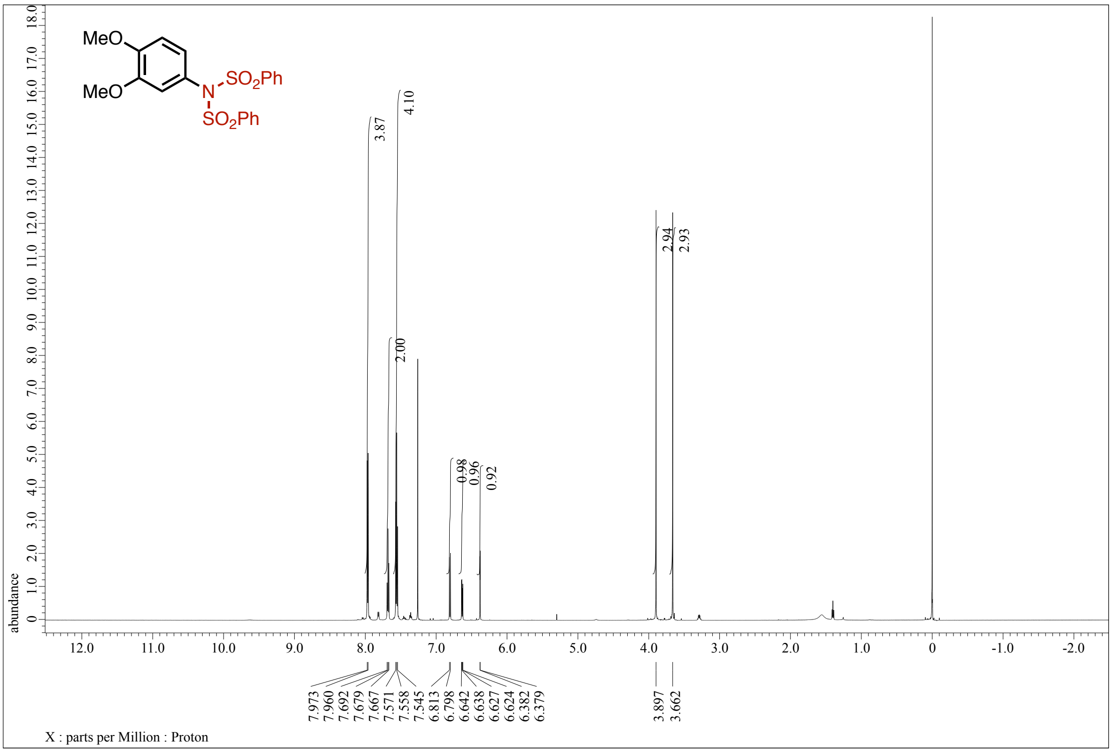

Figure S 6. ^{1}H NMR spectrum of **22**.

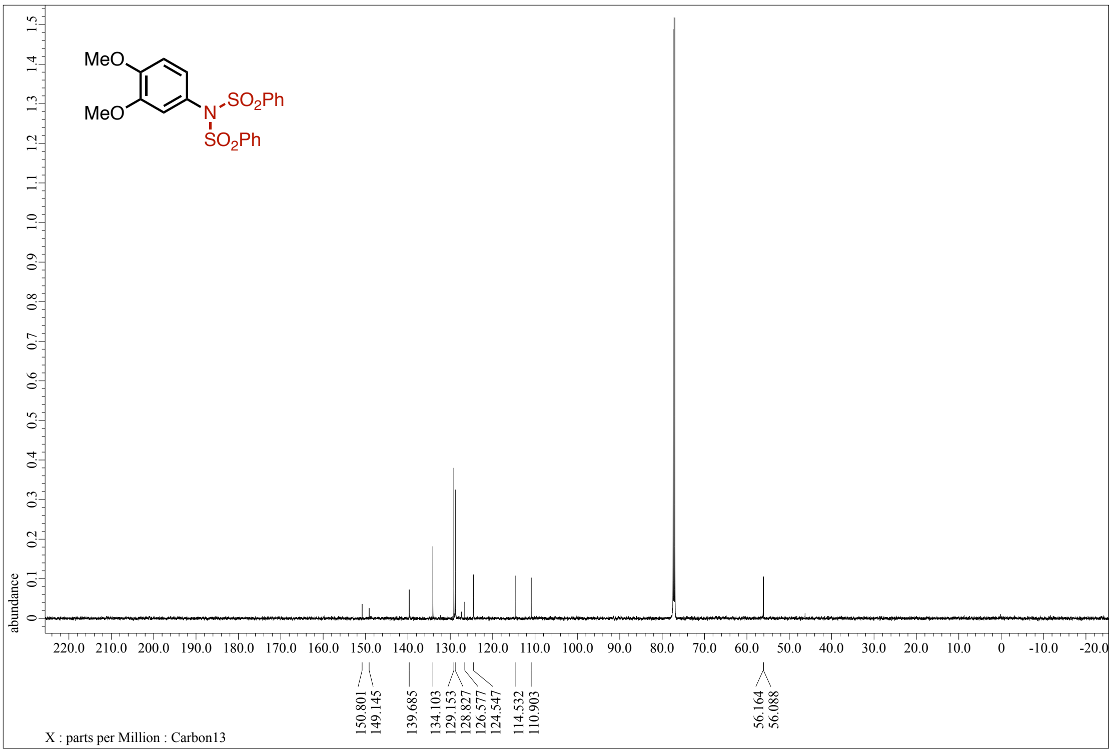

Figure S 7. ^{13}C NMR spectrum of **22**.

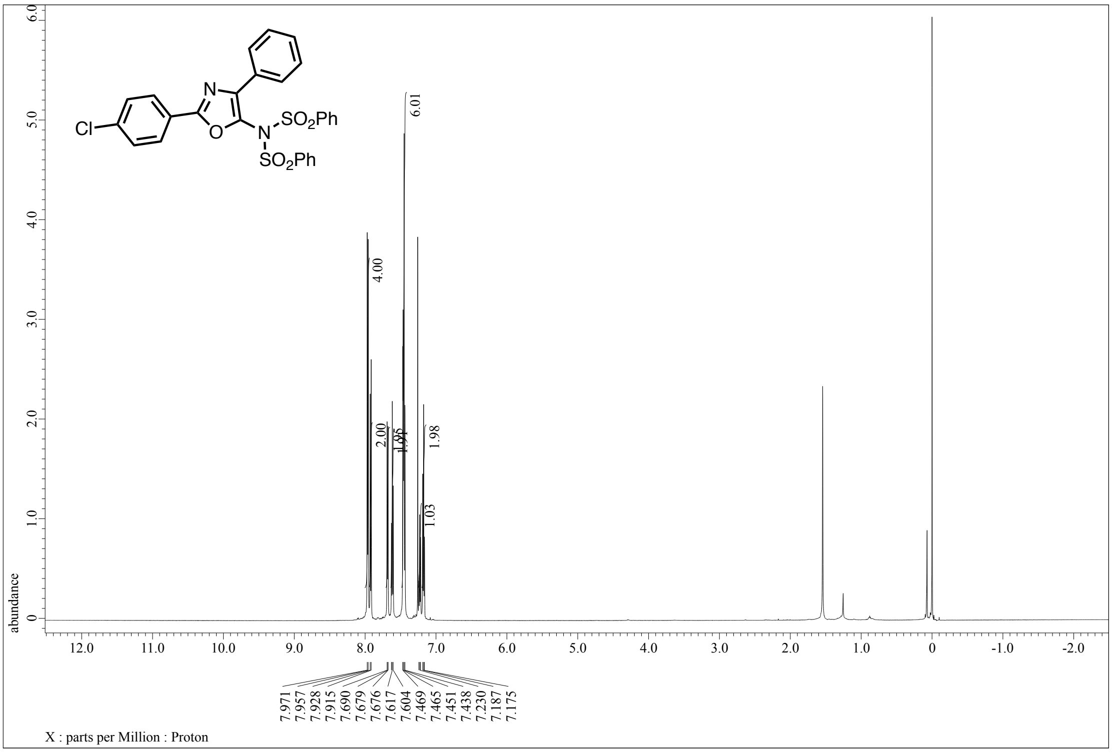

Figure S 8. ^{1}H NMR spectrum of **23**.

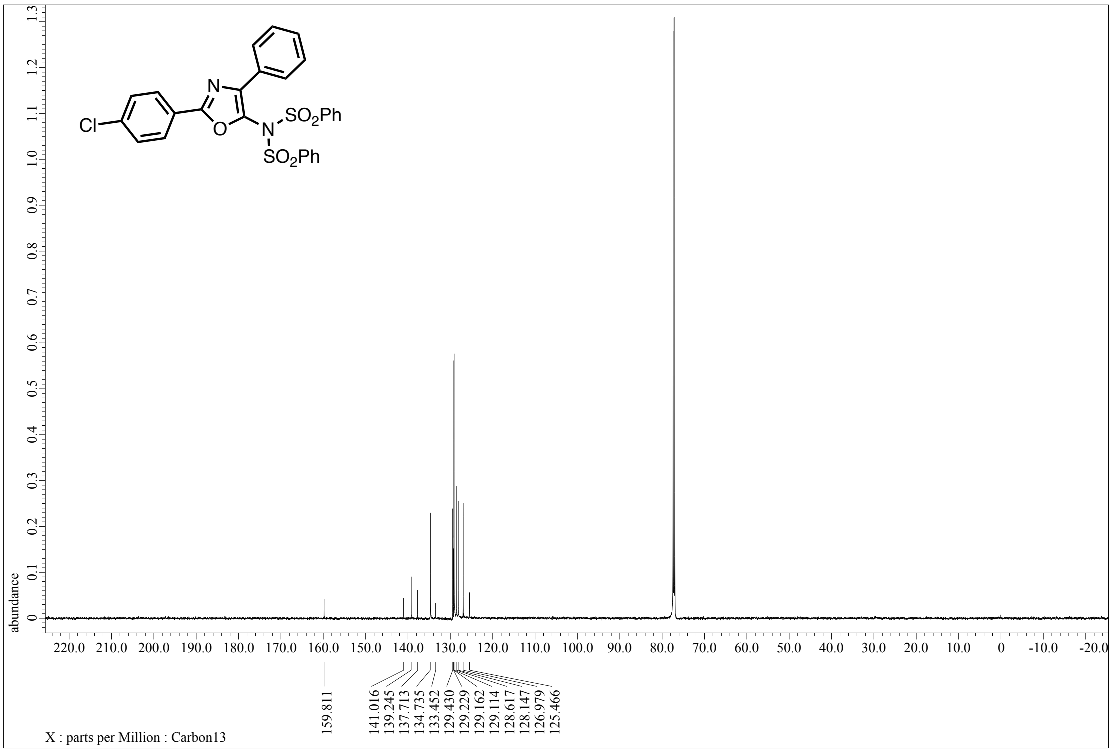

Figure S 9. ^{13}C NMR spectrum of **23**.

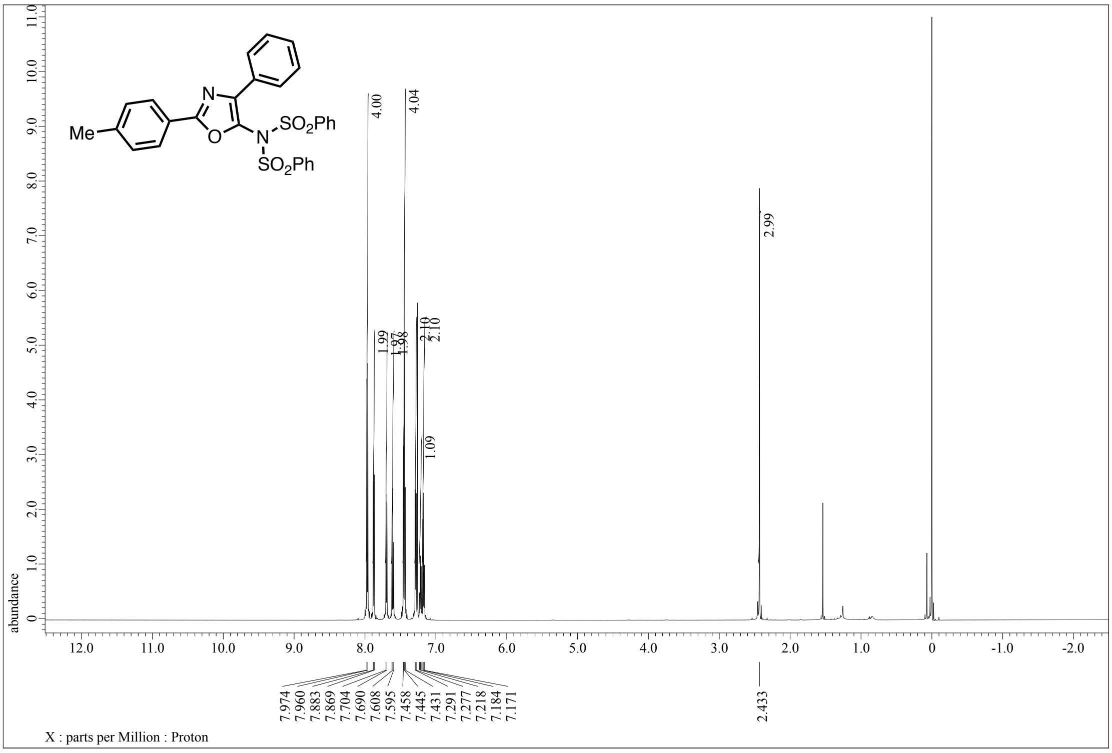

Figure S 10. ^{1}H NMR spectrum of **24**.

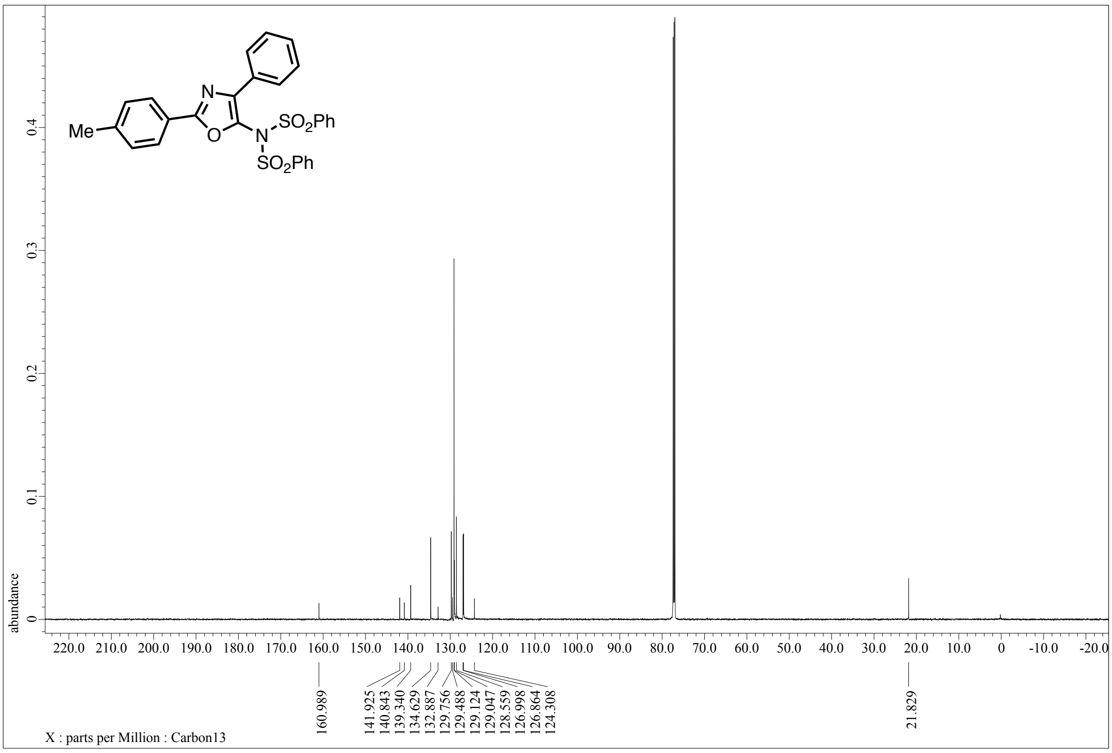

Figure S 11. ^{13}C NMR spectrum of **24**.

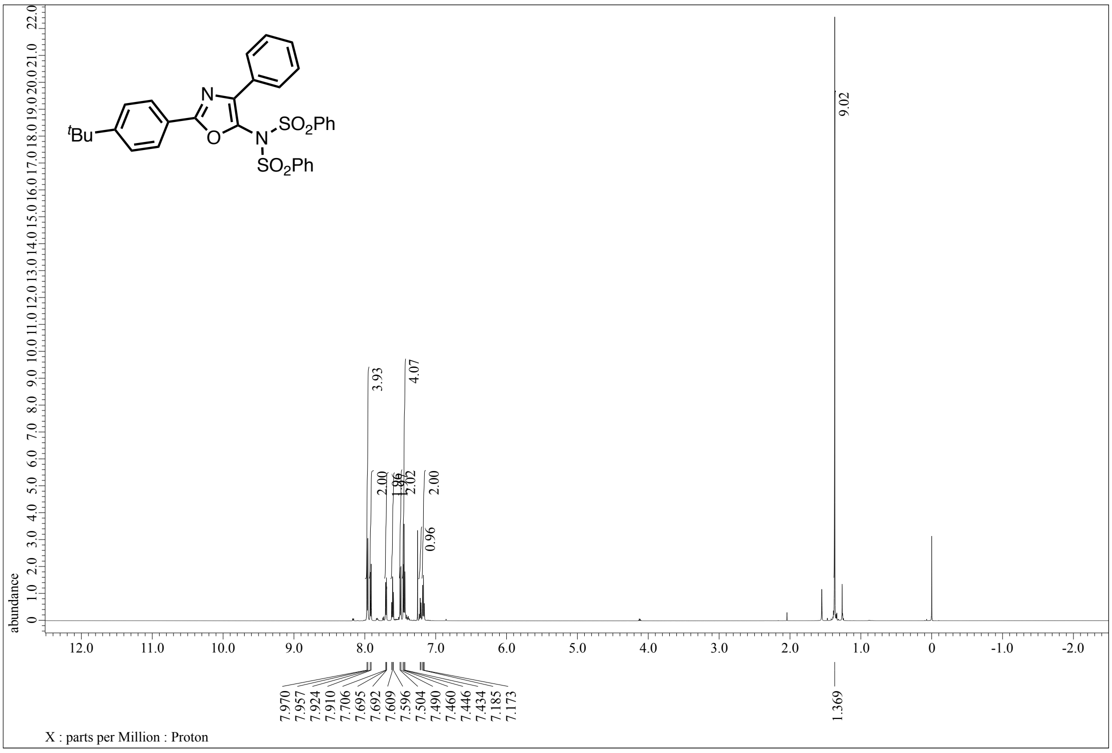

Figure S 12. ^{1}H NMR spectrum of **25**.

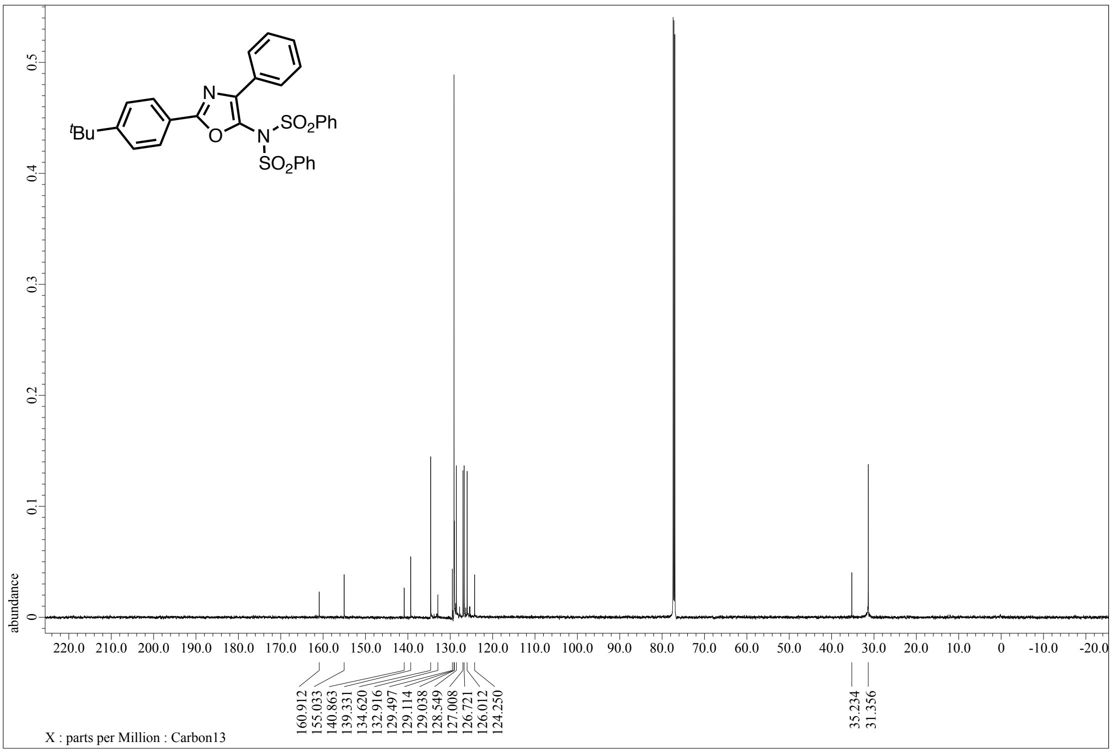

Figure S 13. ^{13}C NMR spectrum of **25**.

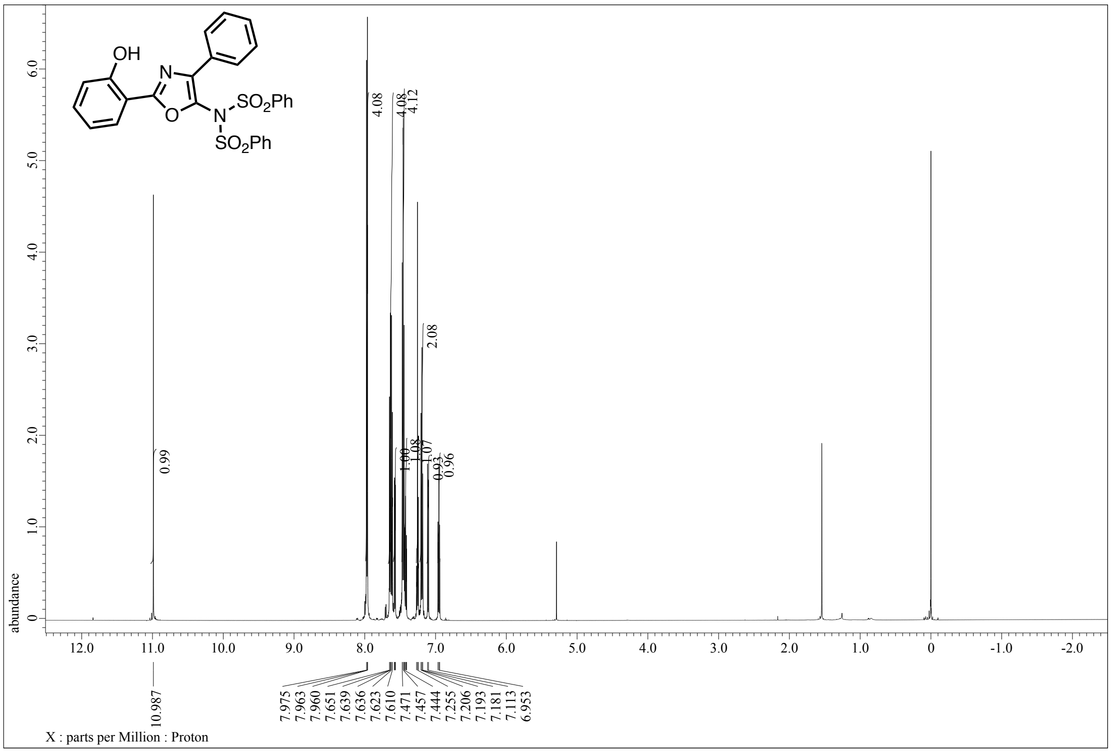

Figure S 14. ^{1}H NMR spectrum of **26**.

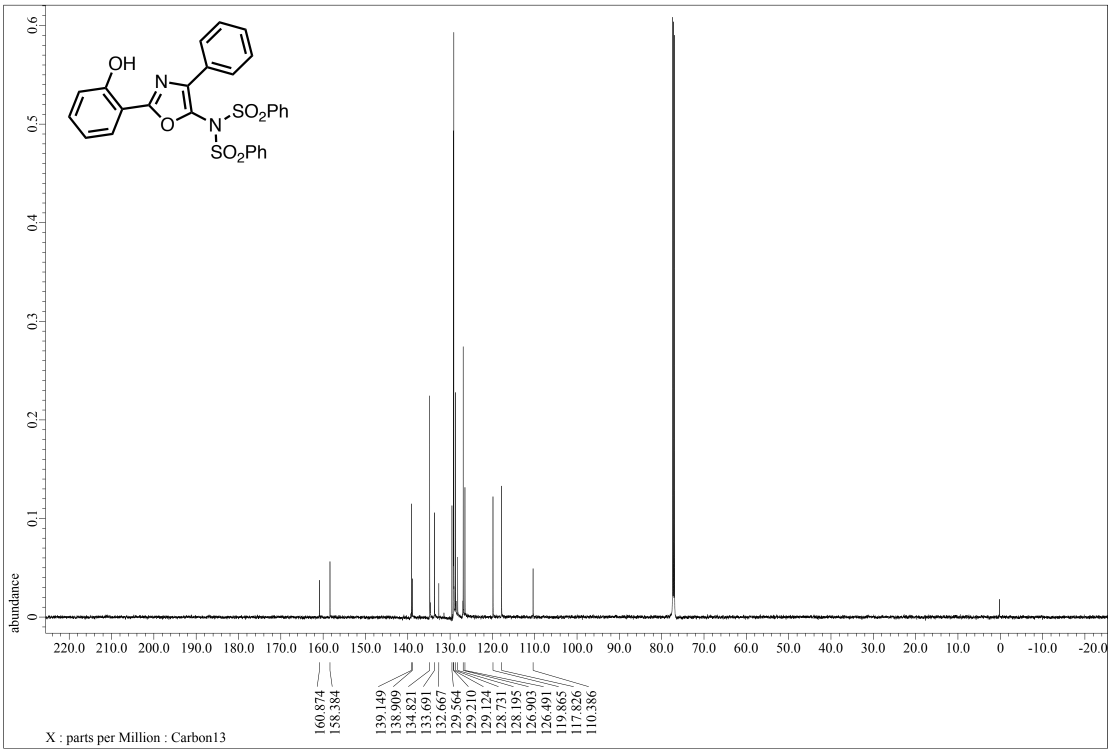

Figure S 15. ^{13}C NMR spectrum of **26**.

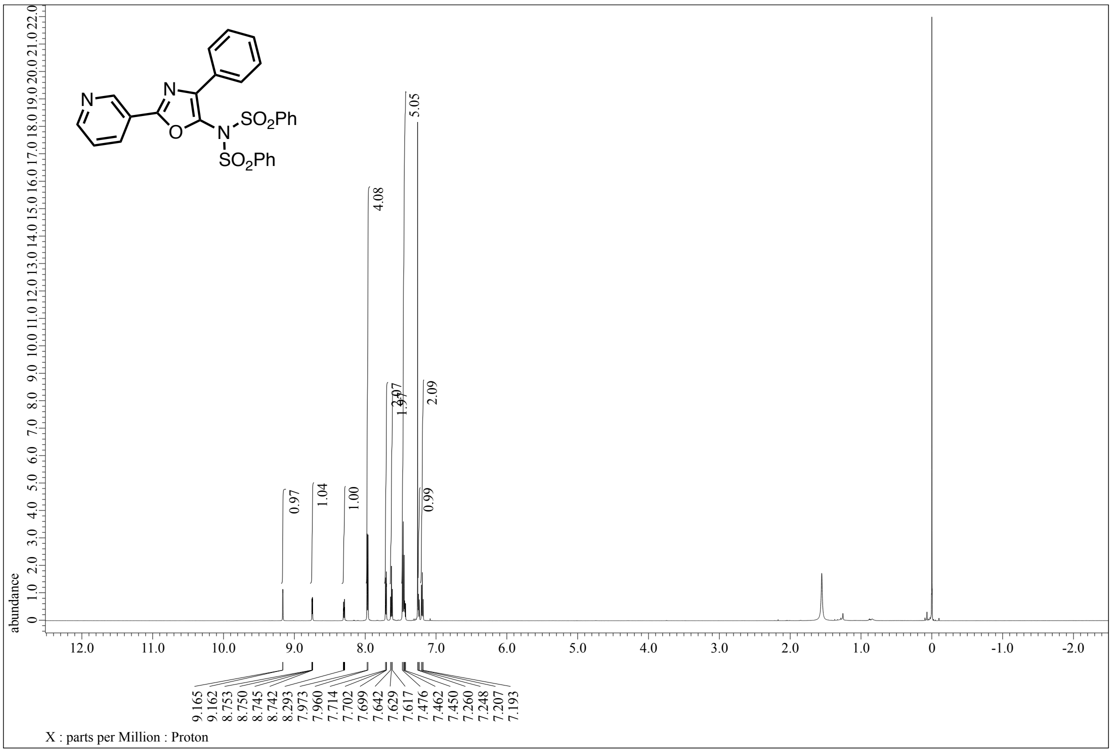

Figure S 16. ^{1}H NMR spectrum of **27**.

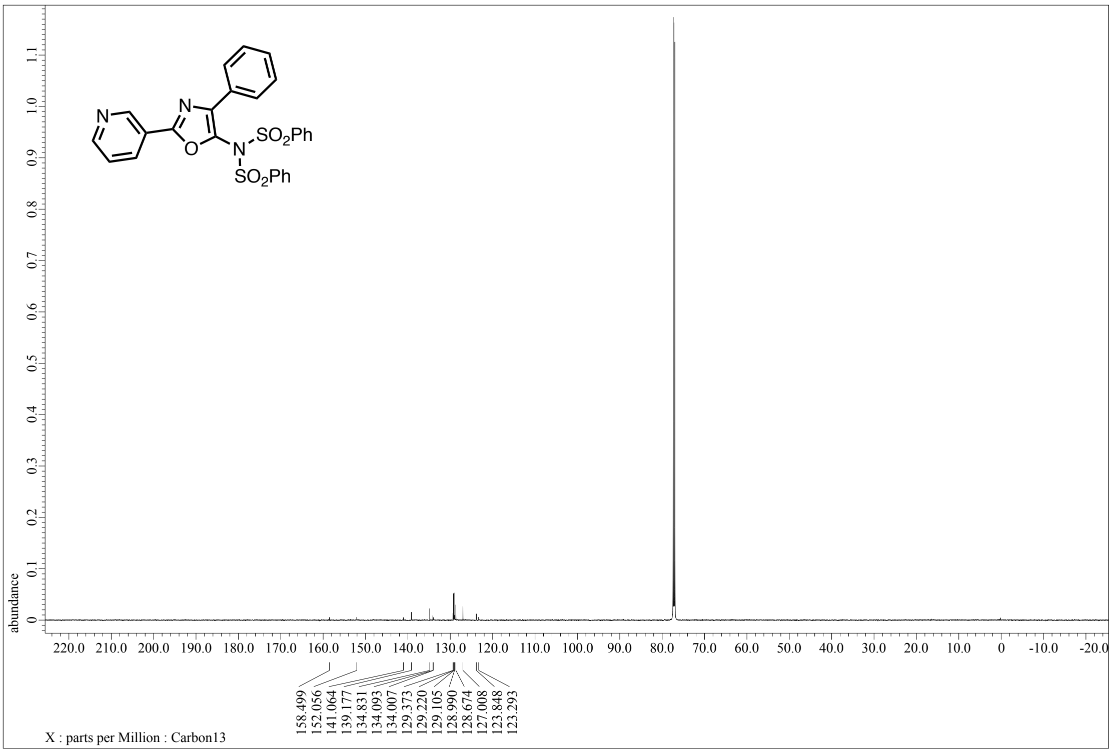

Figure S 17. ^{13}C NMR spectrum of **27**.

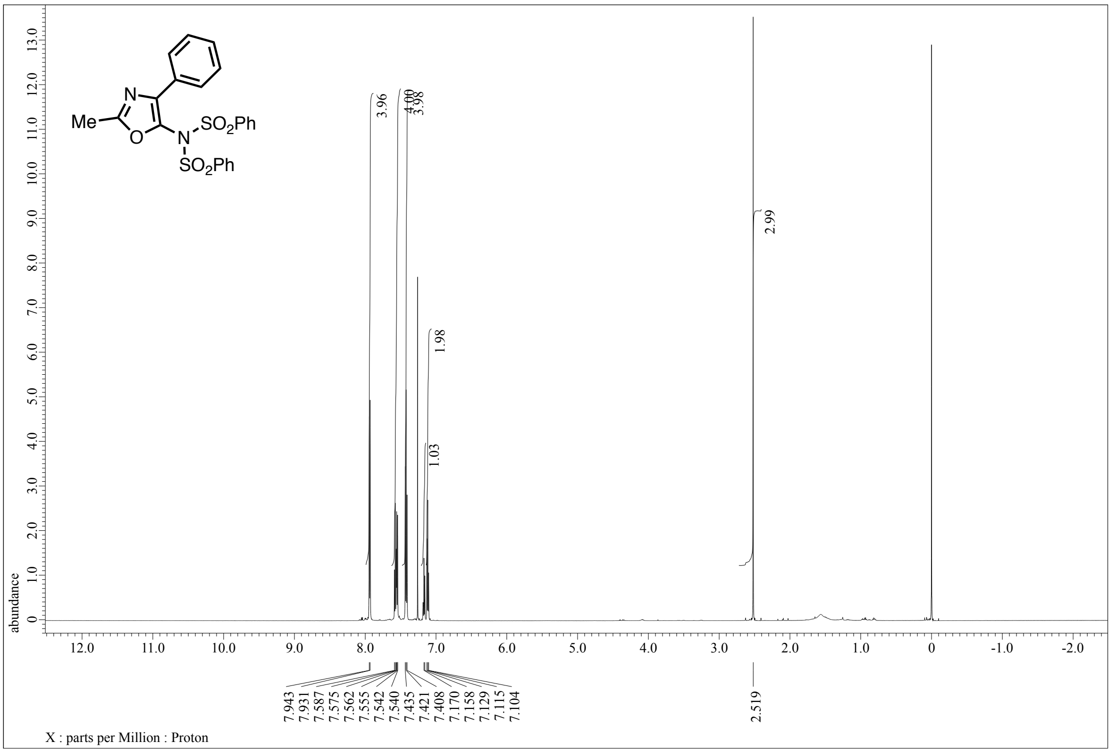

Figure S 18. ^{1}H NMR spectrum of **29**.

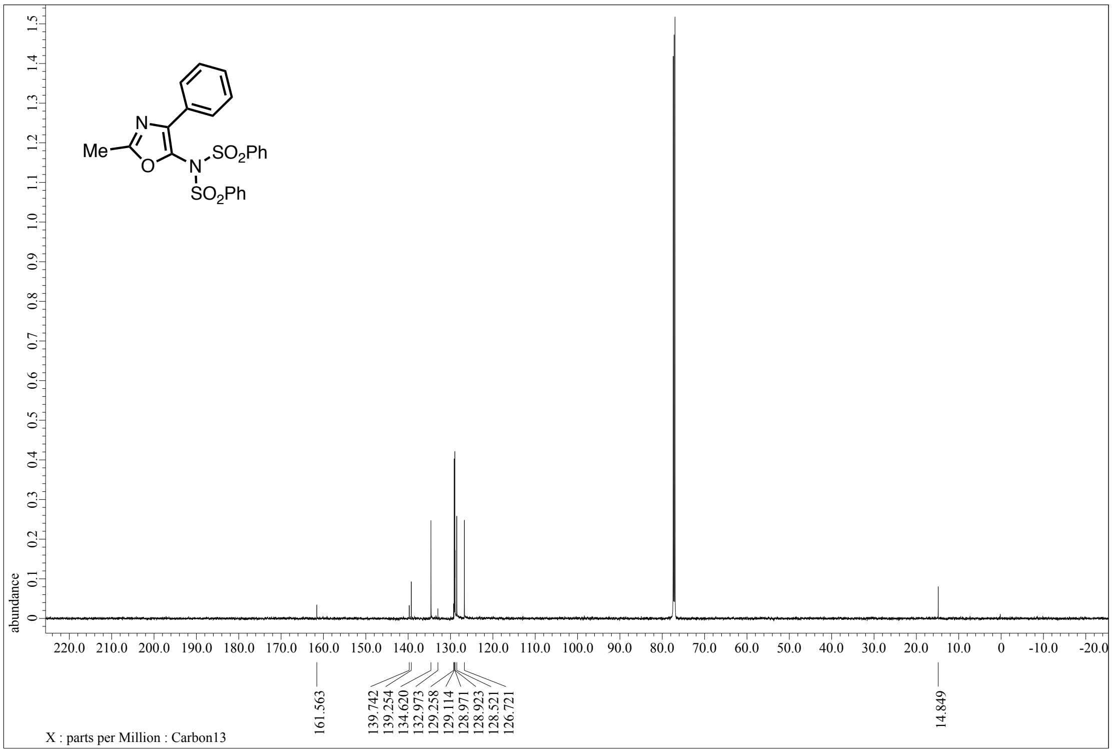

Figure S 19. ^{13}C NMR spectrum of **29**.

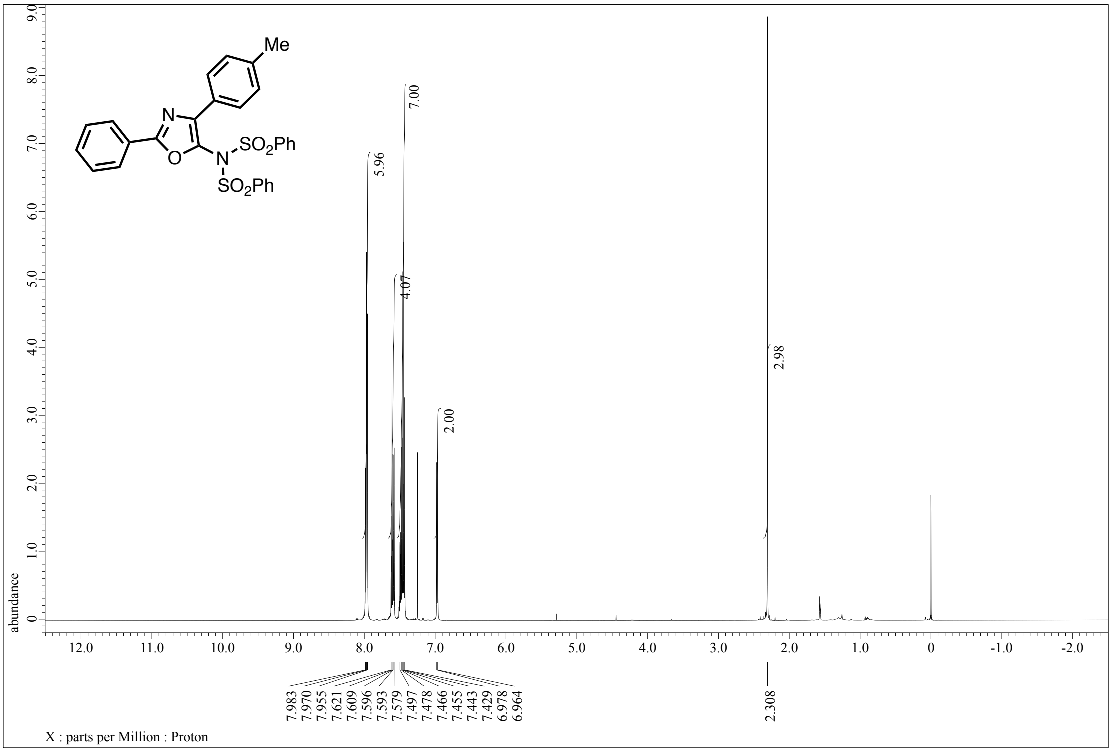

Figure S 20. ^{1}H NMR spectrum of **31**.

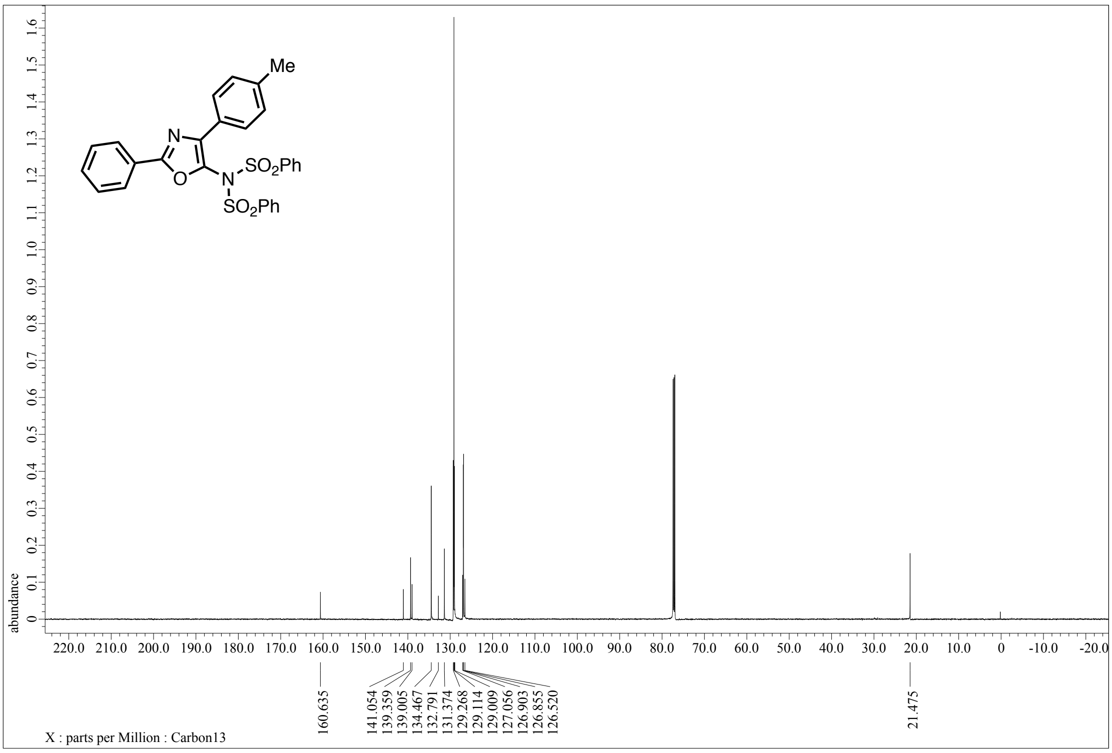

Figure S 21. ^{13}C NMR spectrum of **31**.

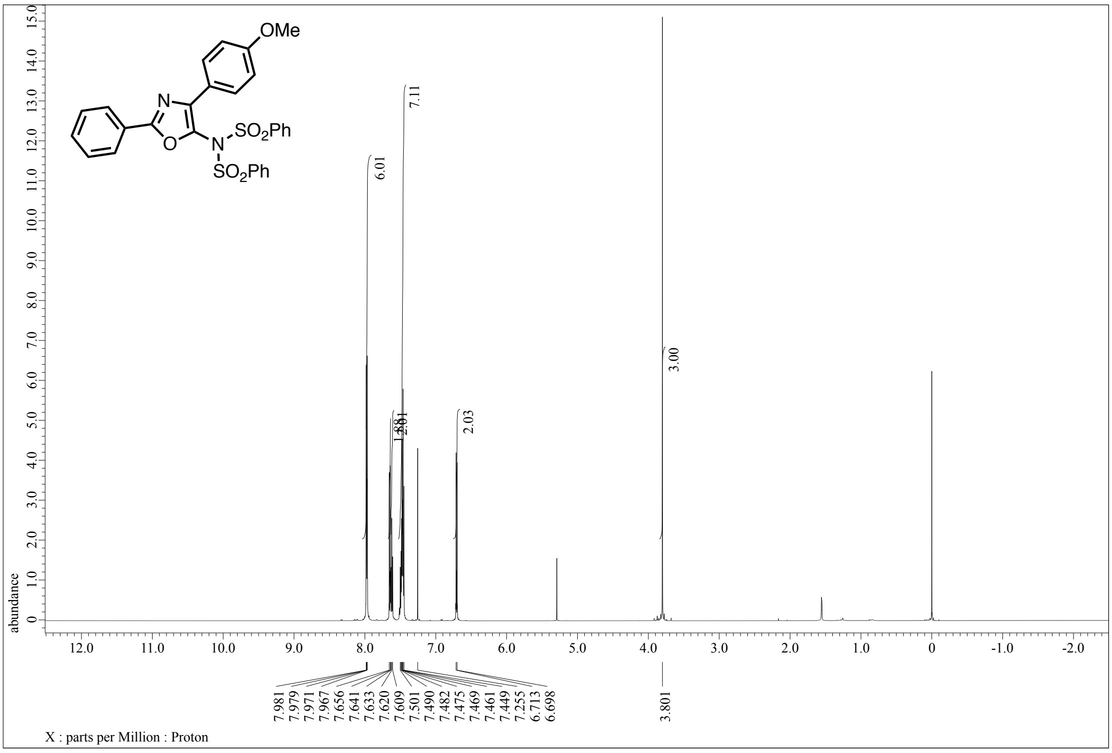

Figure S 22. ^{1}H NMR spectrum of **32.**

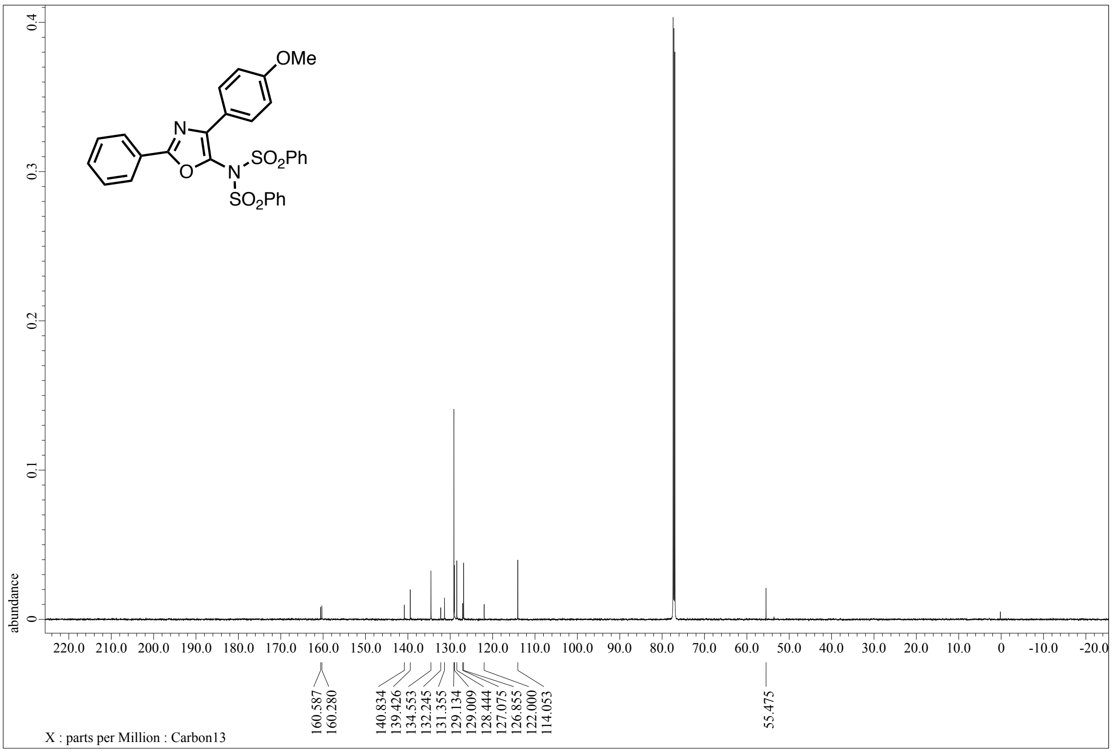

Figure S 23. ^{13}C NMR spectrum of **32**.

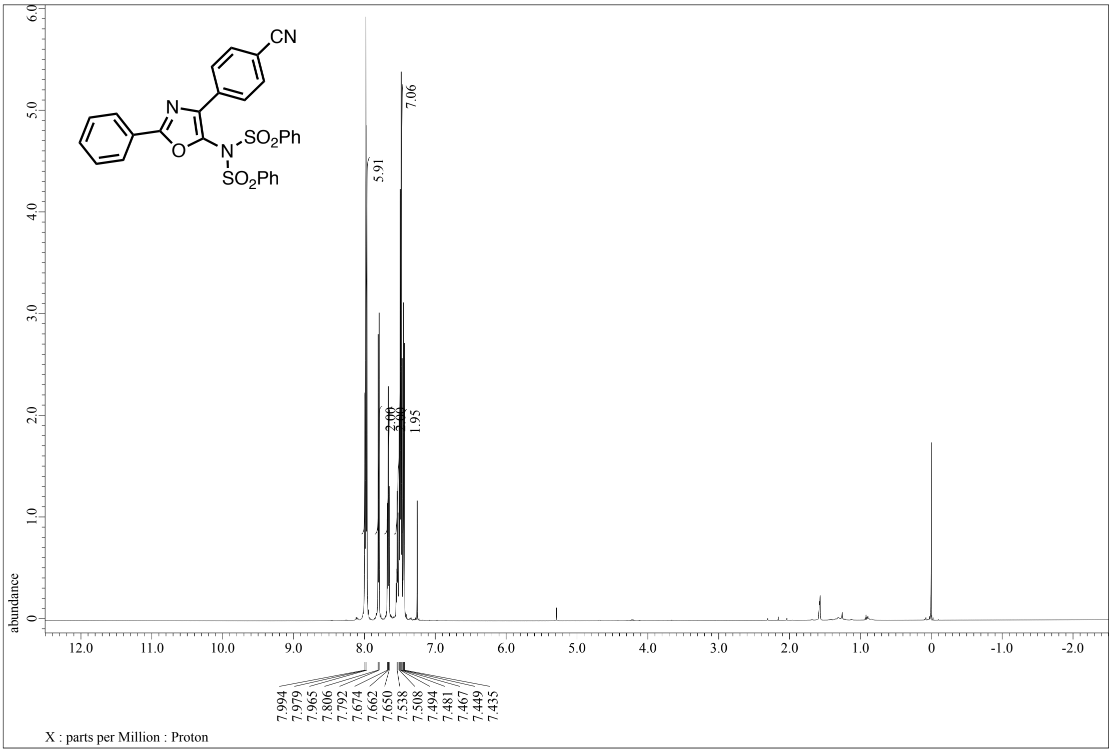

Figure S 24. ^{1}H NMR spectrum of **33**.

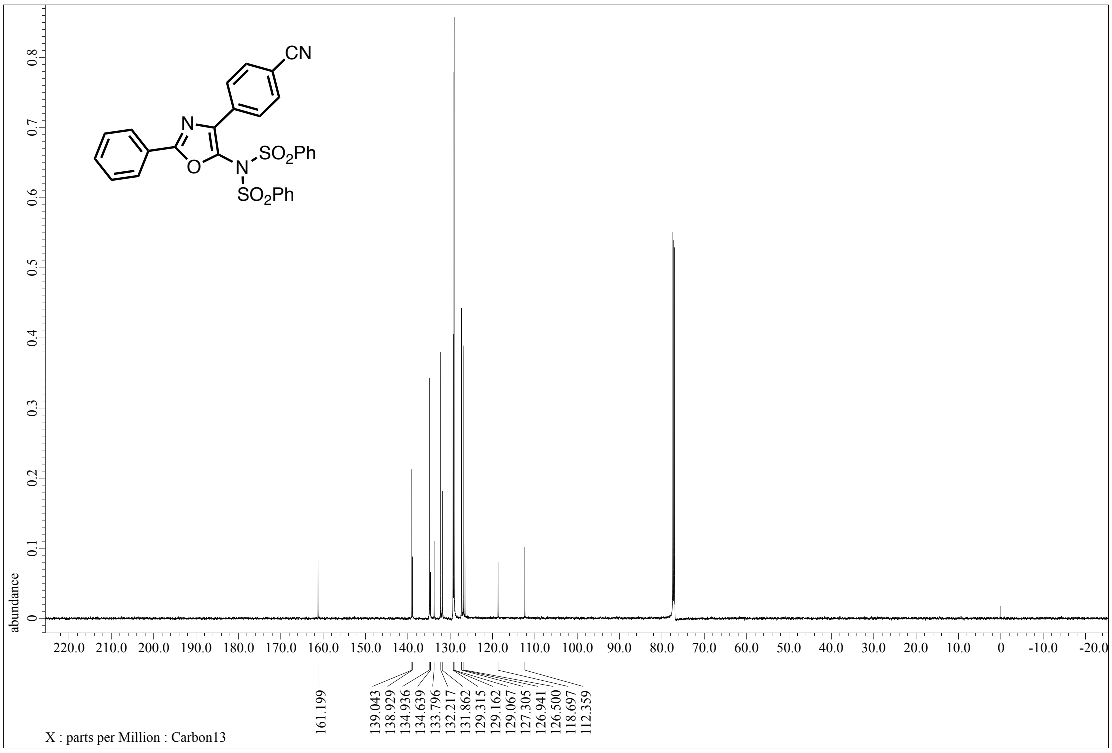

Figure S 25. ^{13}C NMR spectrum of **33**.

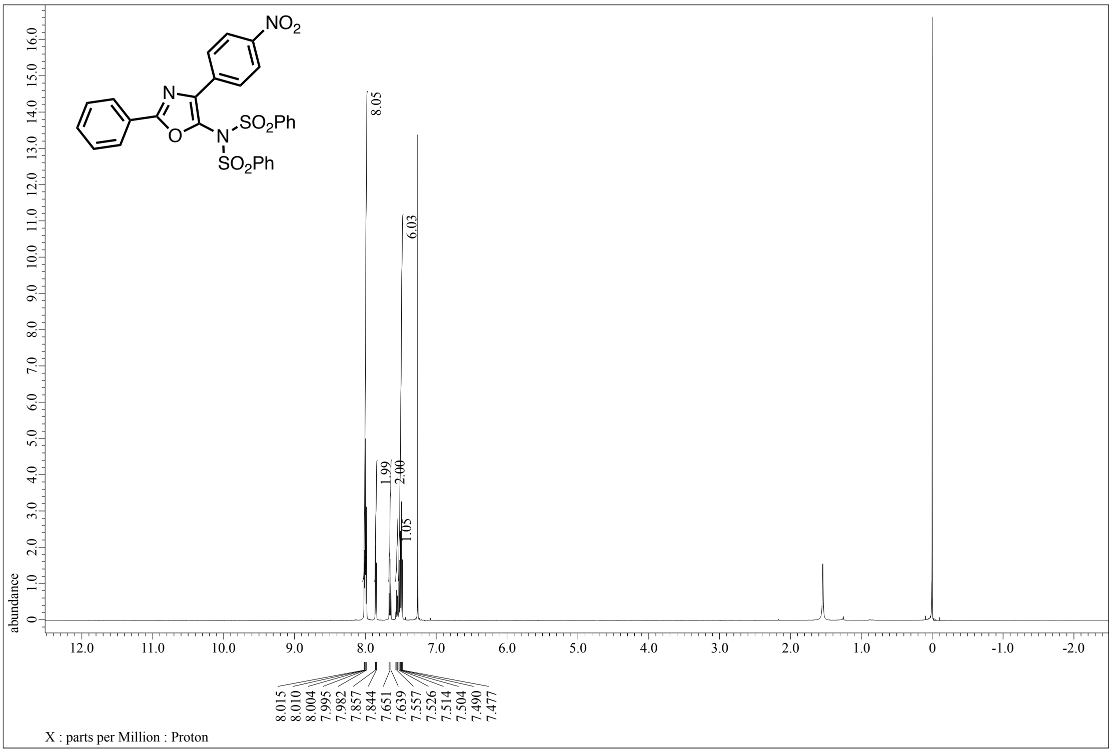

Figure S 26. ^{1}H NMR spectrum of **34**.

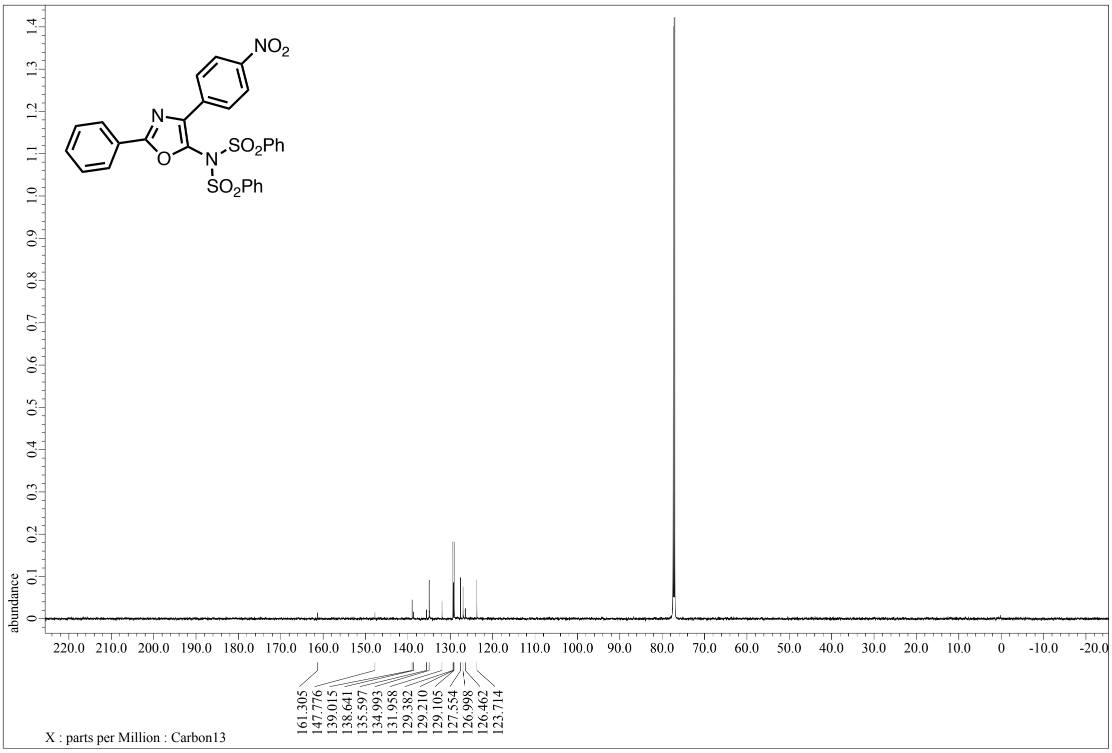

Figure S 27. ^{13}C NMR spectrum of **34**.

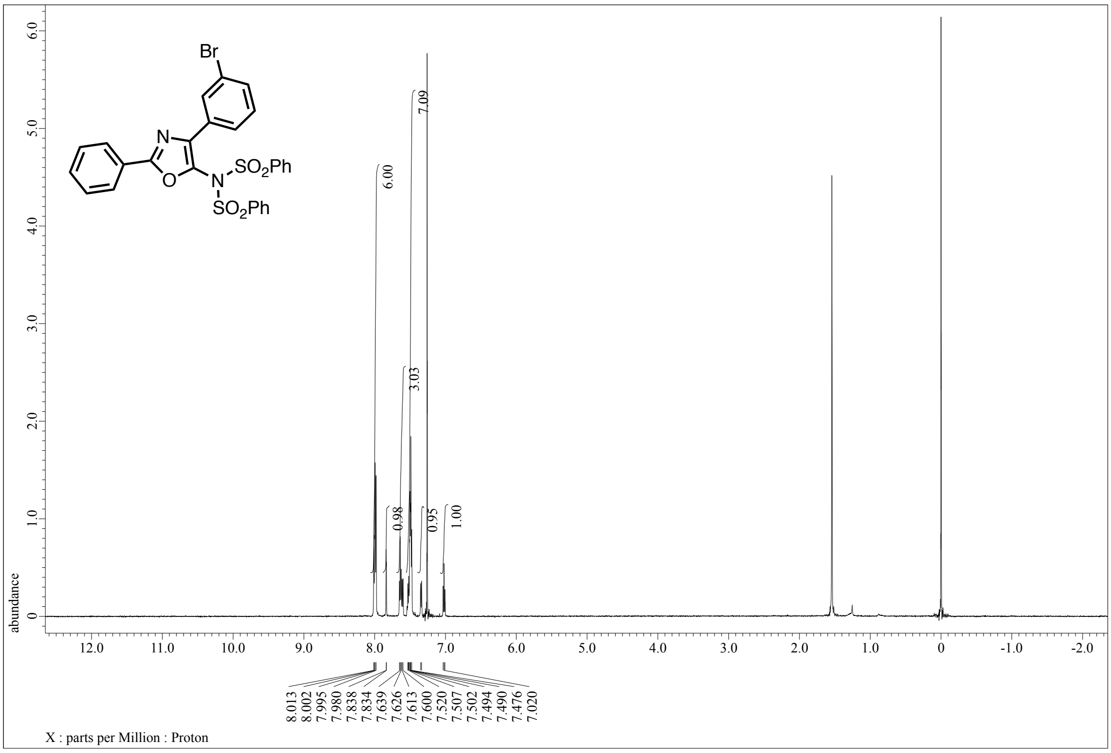

Figure S 28. ^{1}H NMR spectrum of **35**.

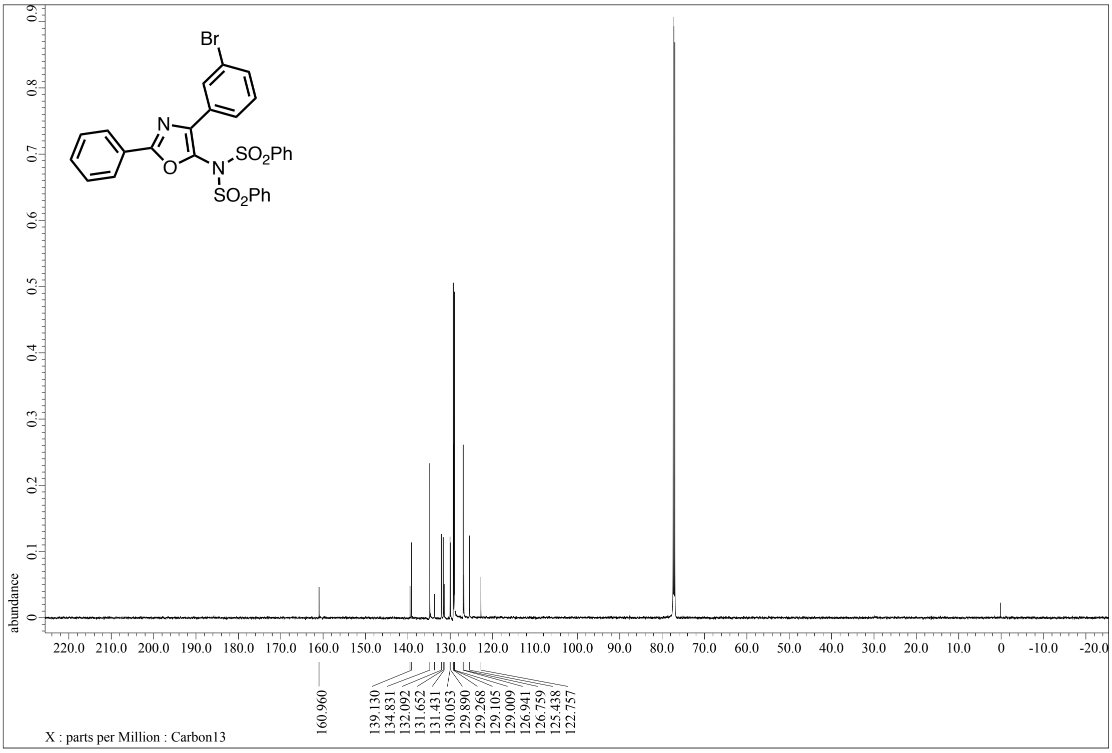

Figure S 29. ^{13}C NMR spectrum of **35**.

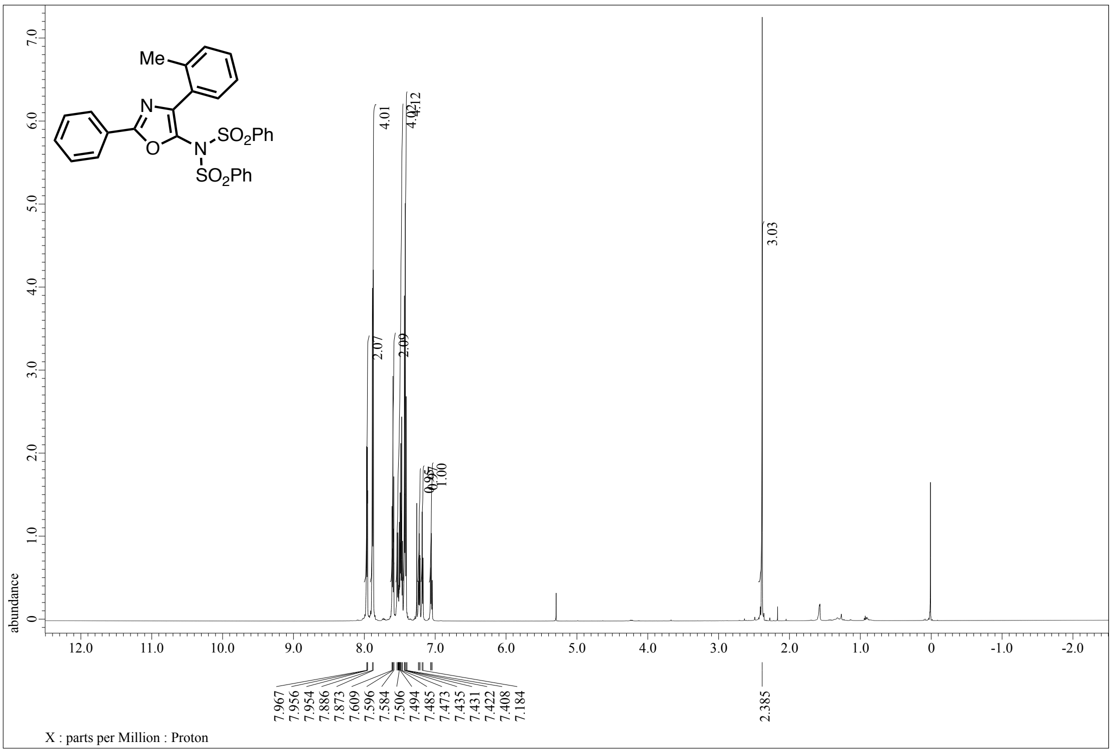

Figure S 30. ^{1}H NMR spectrum of **36**.

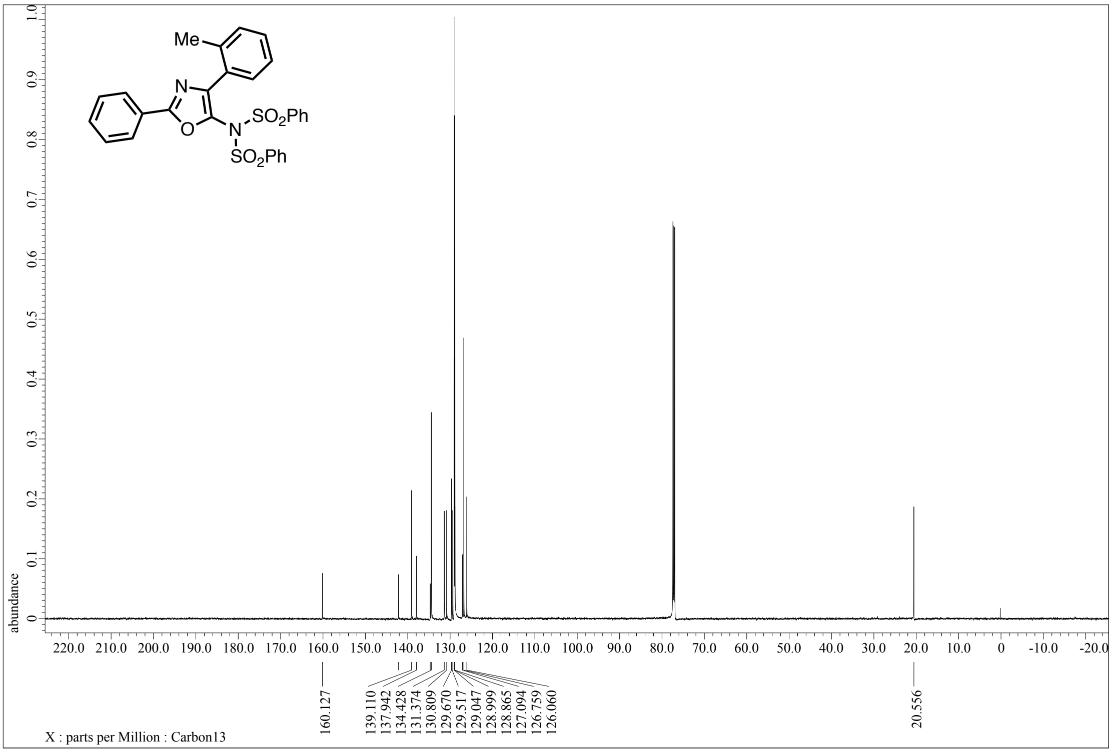

Figure S 31. ^{13}C NMR spectrum of **36**.

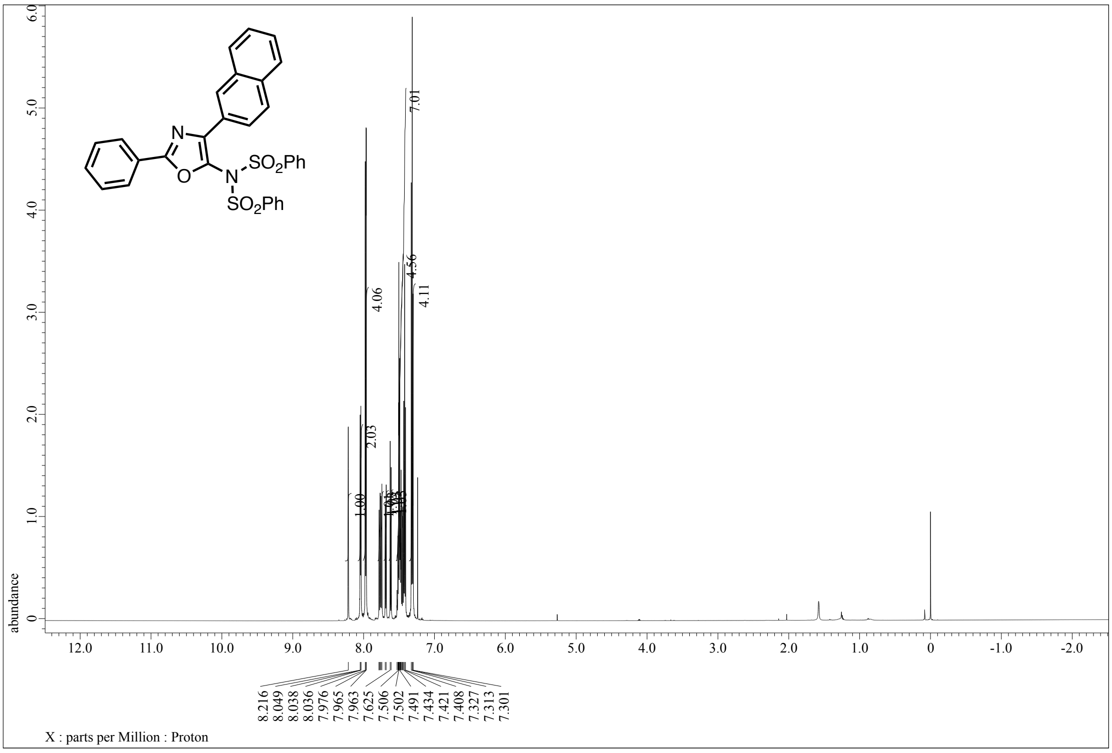

Figure S 32. ^{1}H NMR spectrum of **37**.

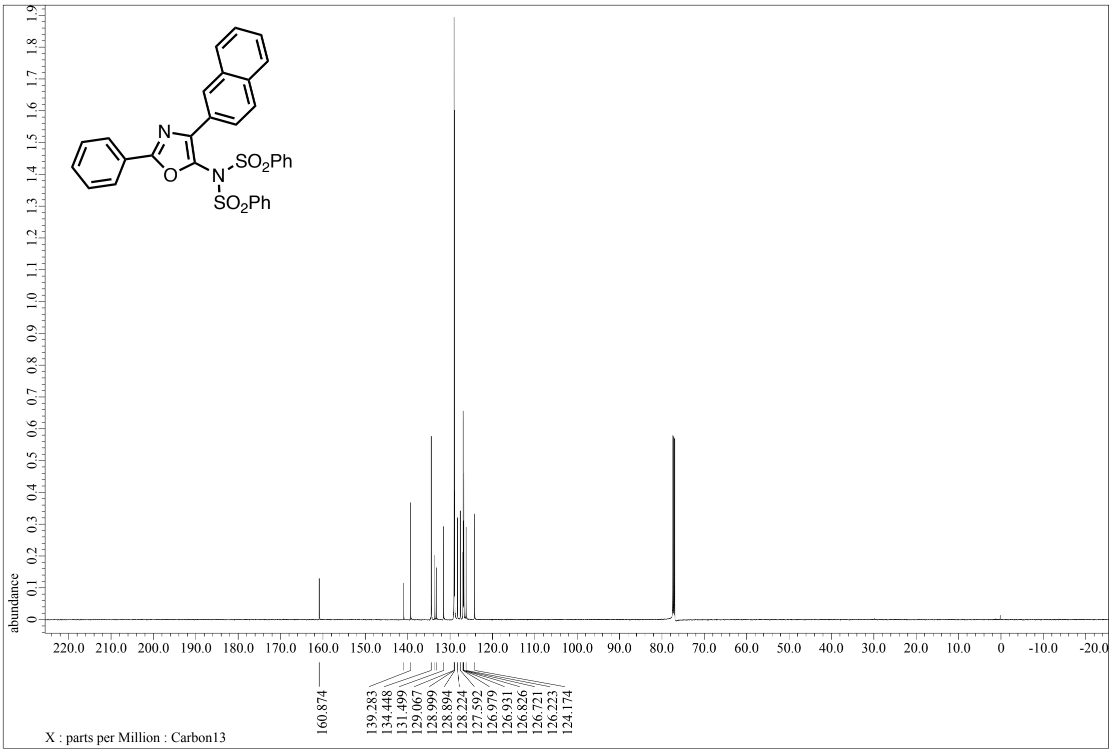

Figure S 33. ^{13}C NMR spectrum of **37**.

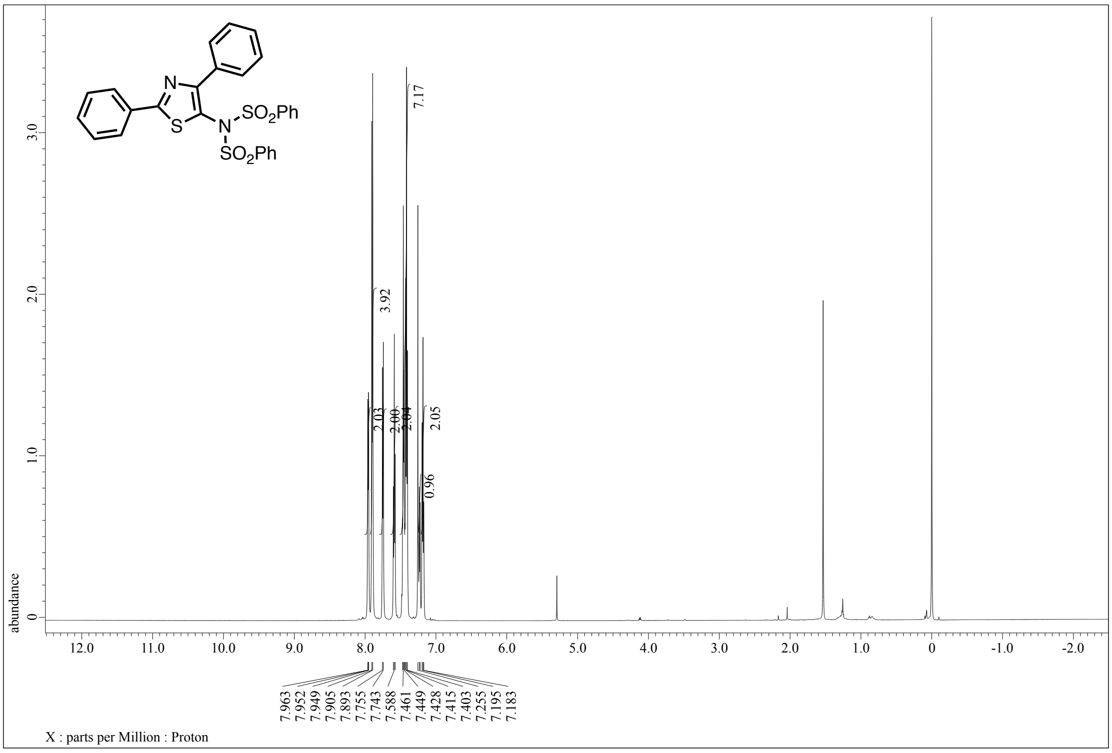

Figure S 34. ^{1}H NMR spectrum of **39**.

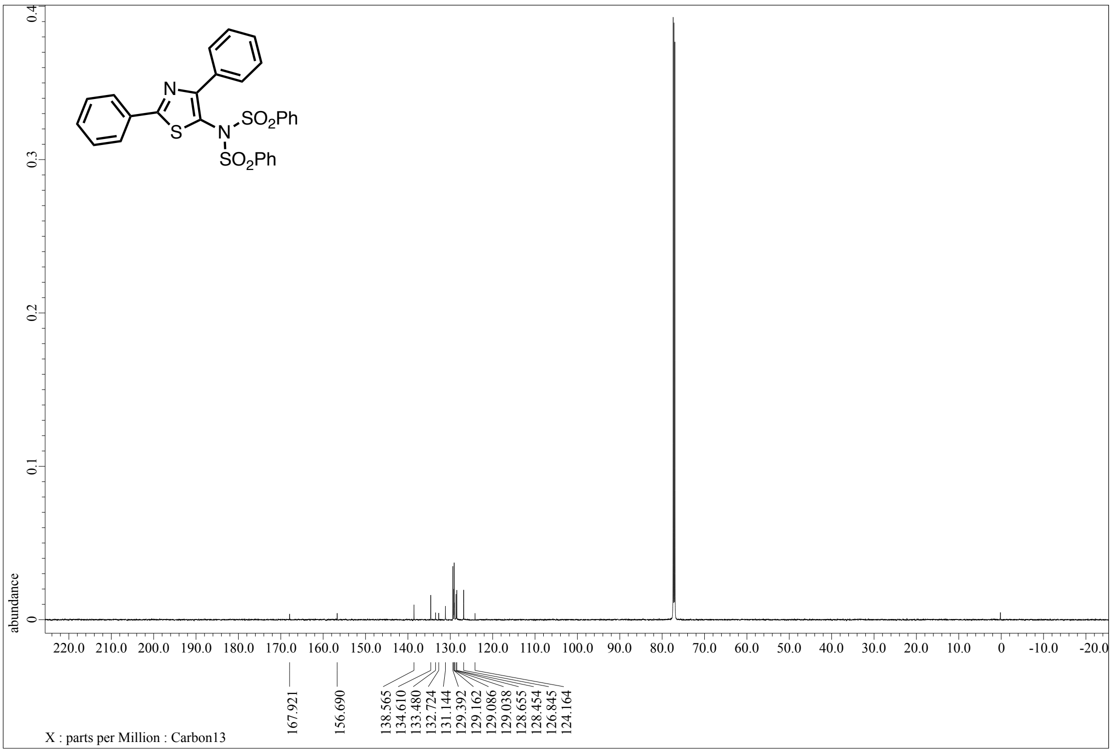

Figure S 35. ^{13}C NMR spectrum of **39**.

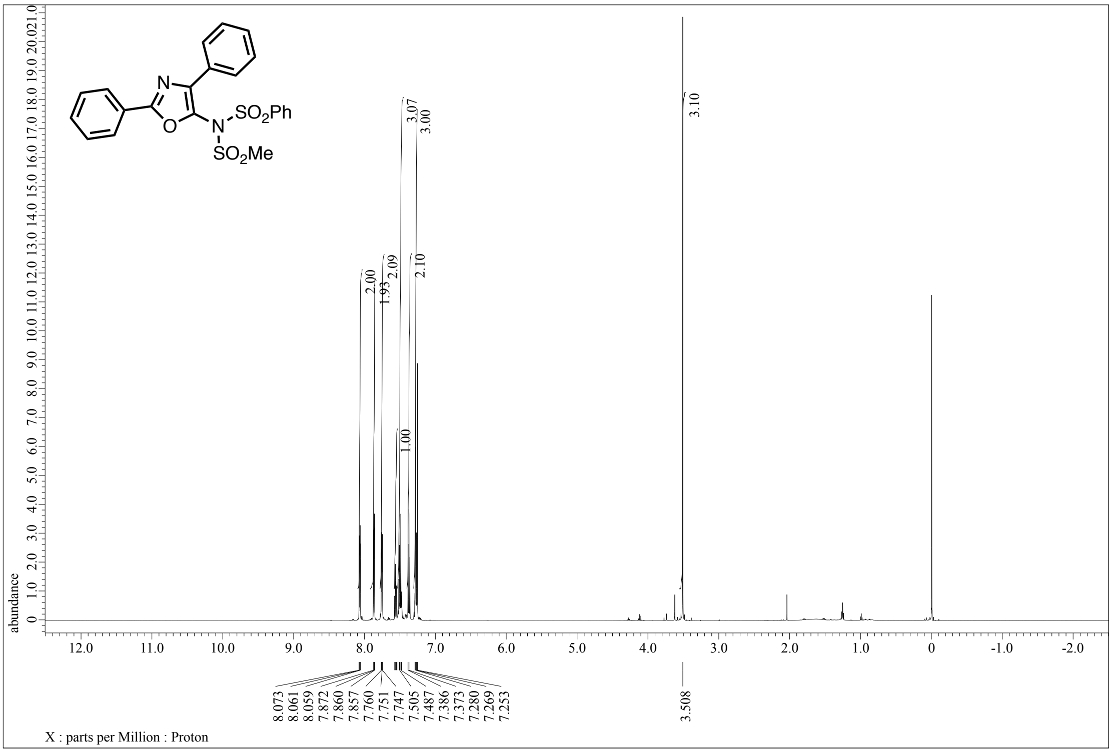

Figure S 36. ^{1}H NMR spectrum of **40**.

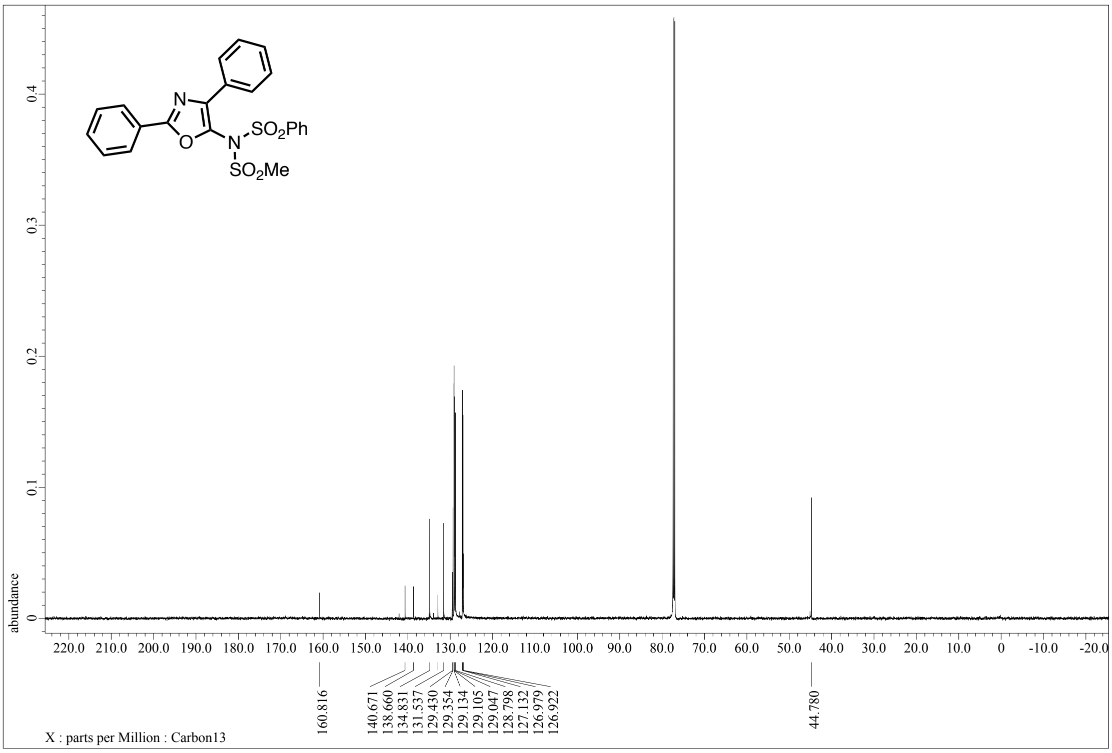

Figure S 37. ^{13}C NMR spectrum of **40**.

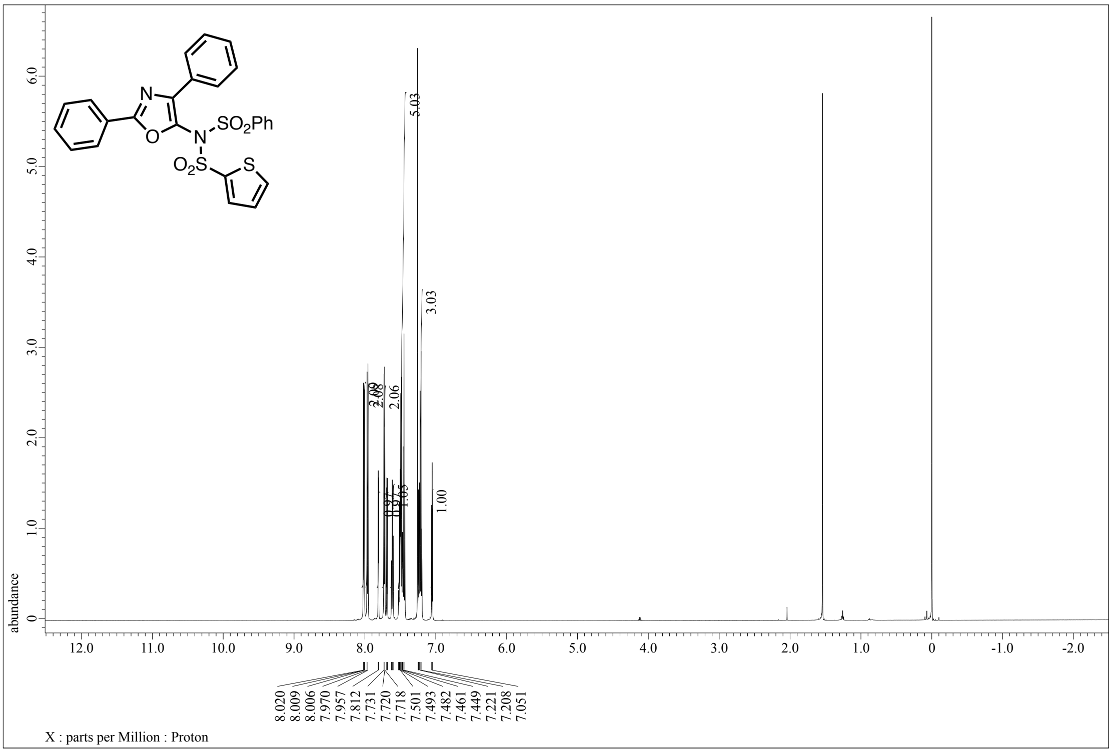

Figure S 38. ^{1}H NMR spectrum of **41**.

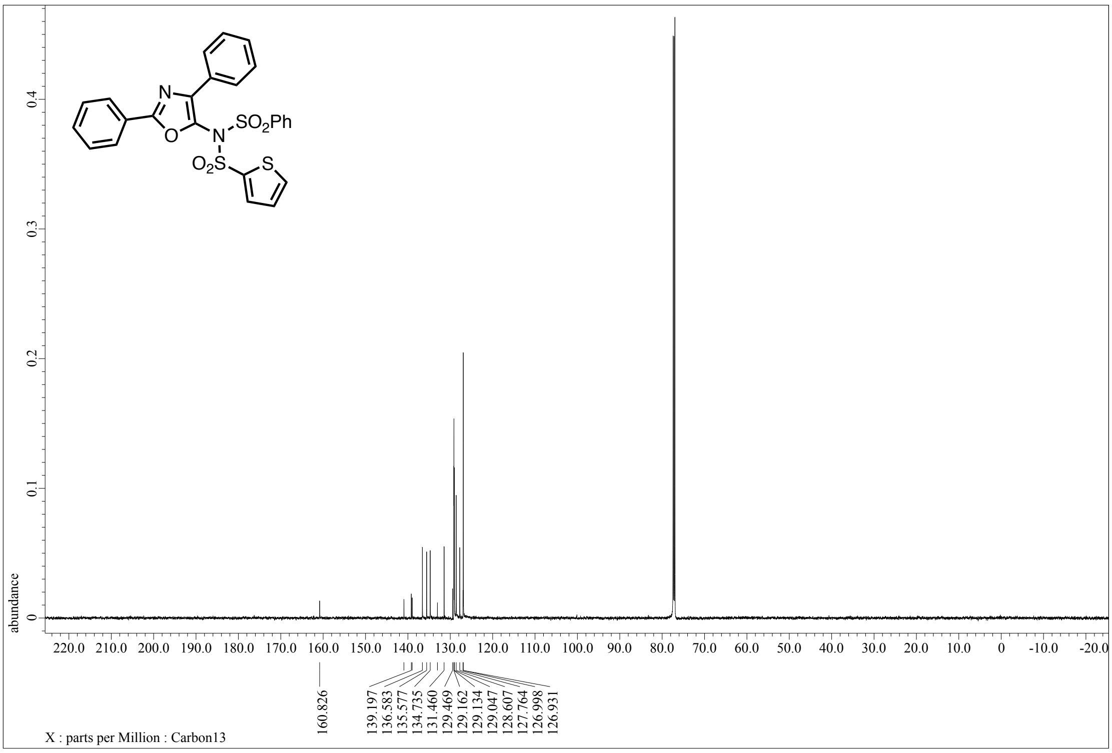

Figure S 39. ^{13}C NMR spectrum of **41**.

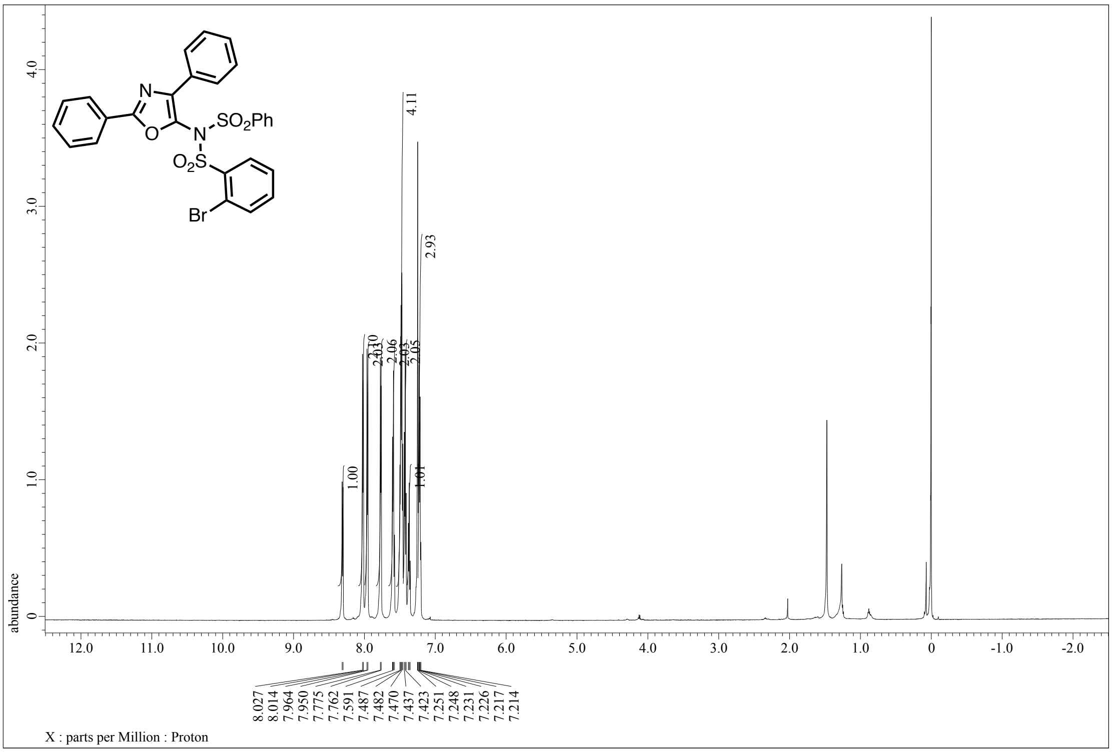

Figure S 40. ^{1}H NMR spectrum of **42**.

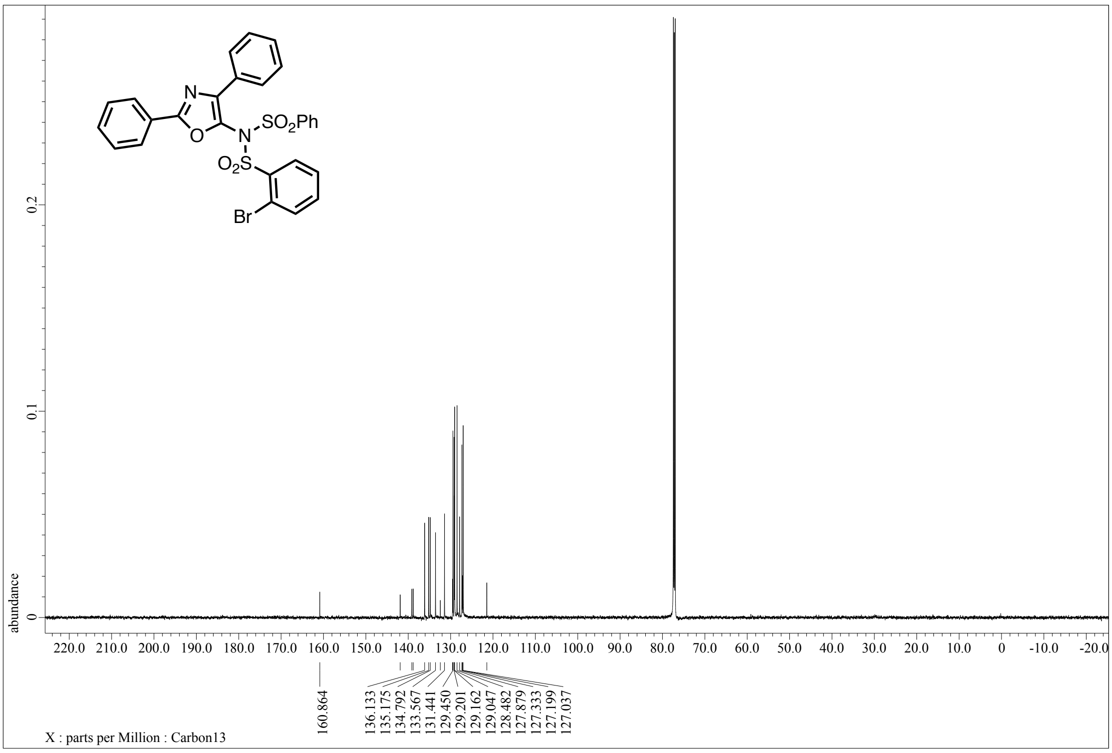

Figure S 41. ^{13}C NMR spectrum of **42**.

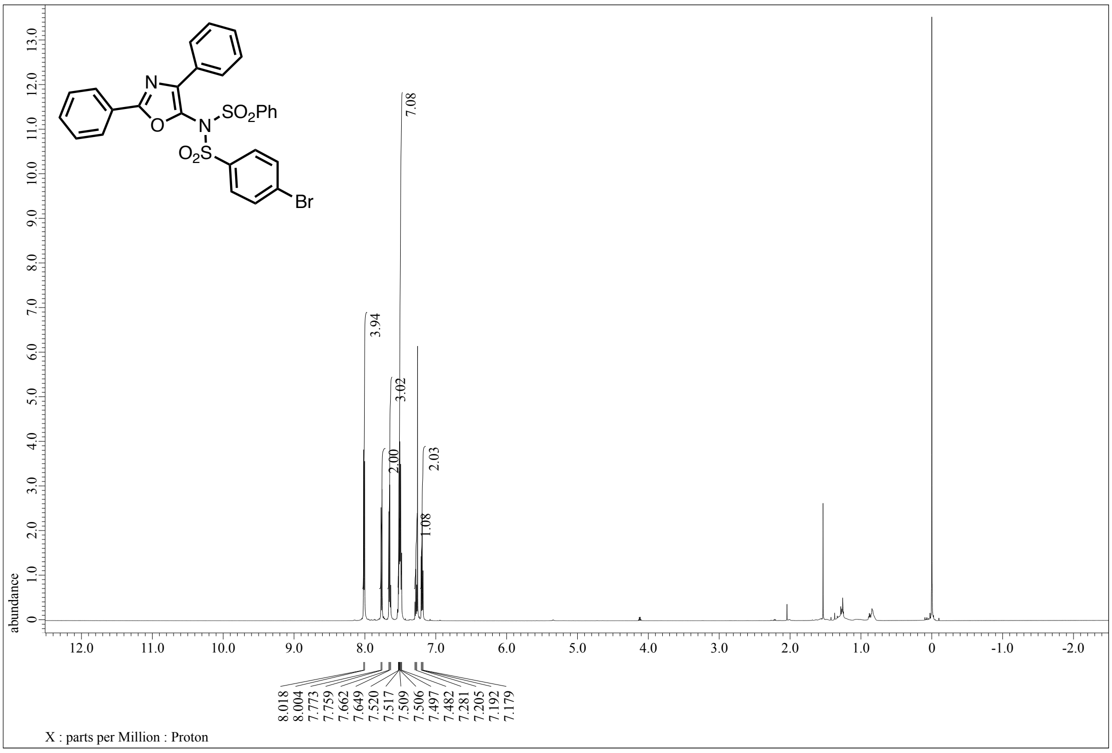

Figure S 42. ^{1}H NMR spectrum of **43**.

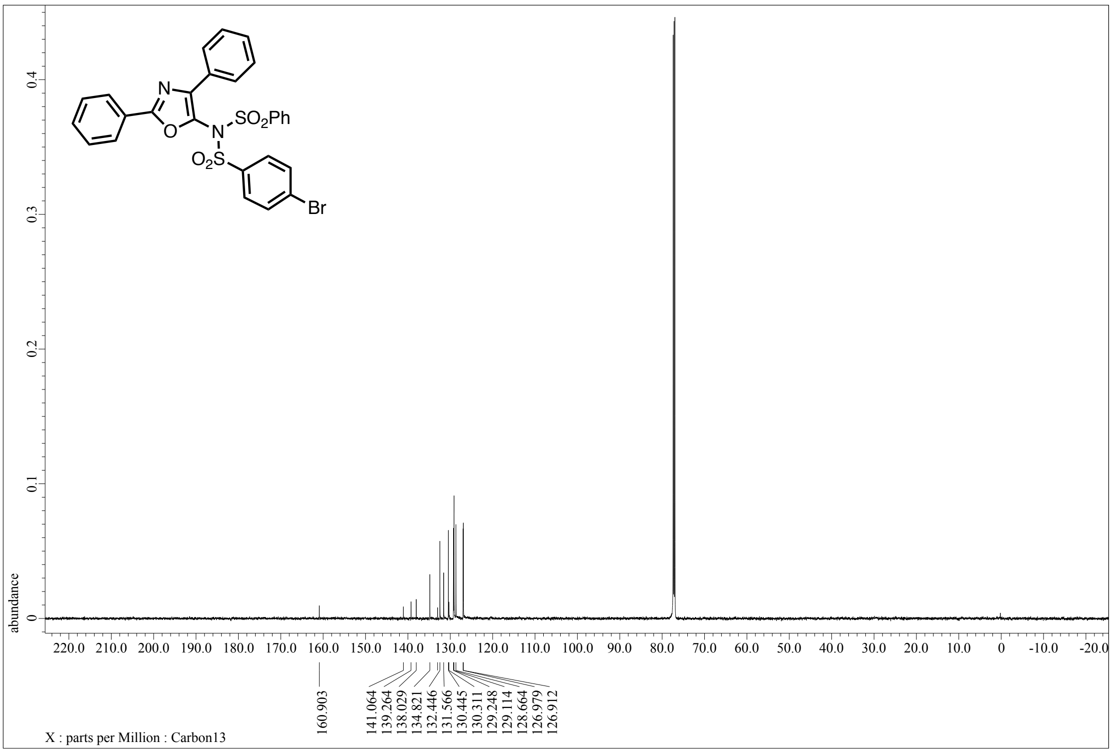

Figure S 43. ^{13}C NMR spectrum of **43**.

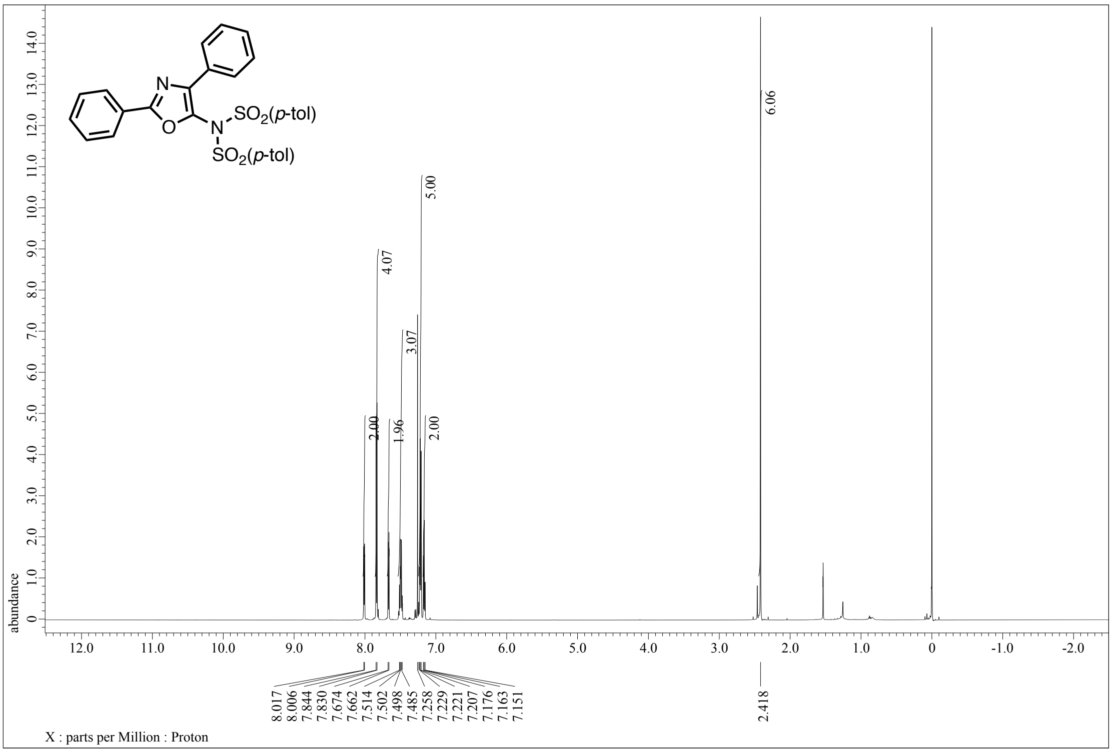

Figure S 44. ^{1}H NMR spectrum of **44**.

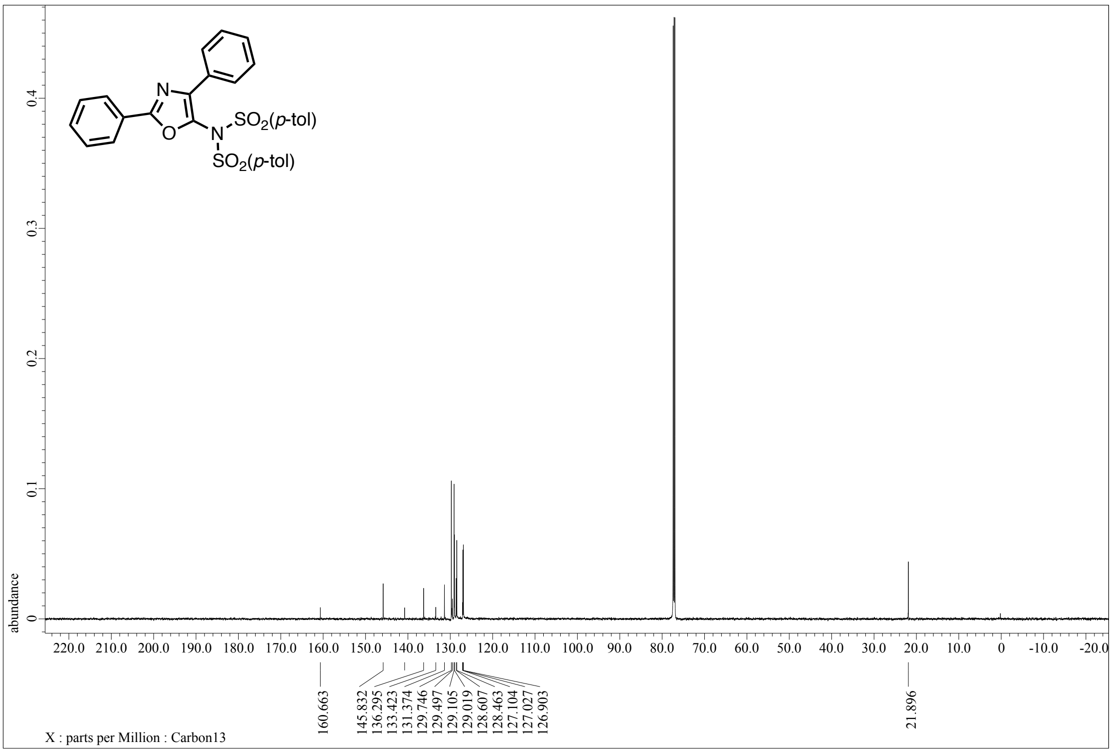

Figure S 45. ^{13}C NMR spectrum of **44**.

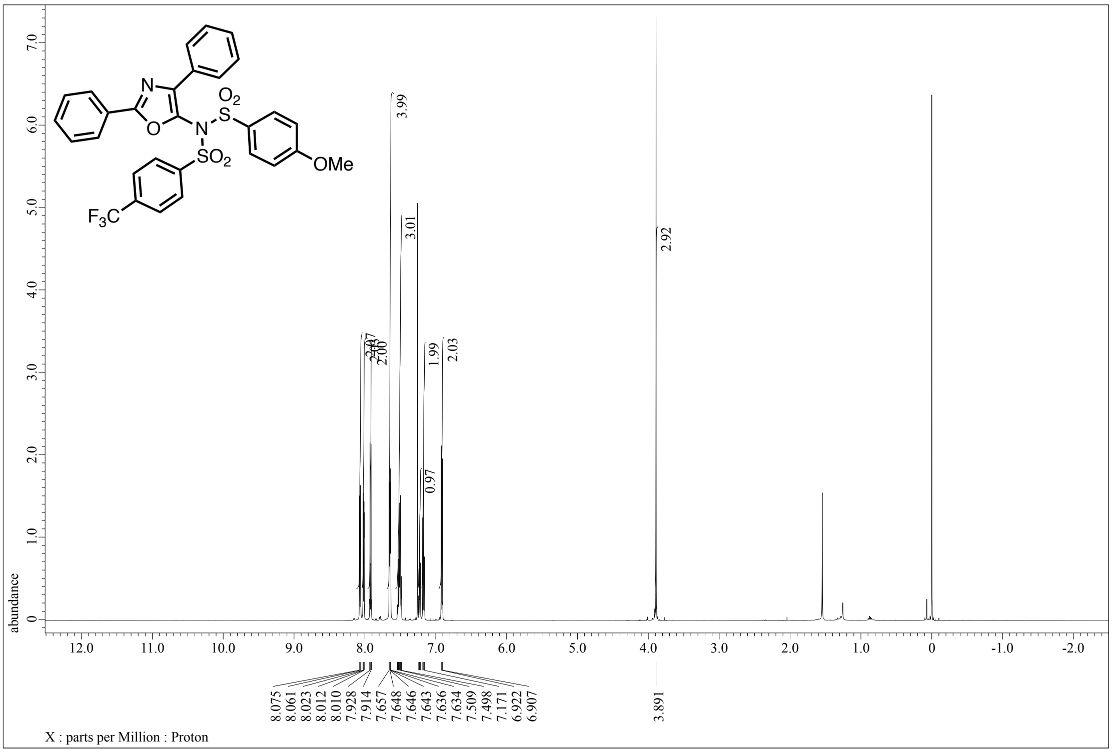

Figure S 46. ^{1}H NMR spectrum of **45**.

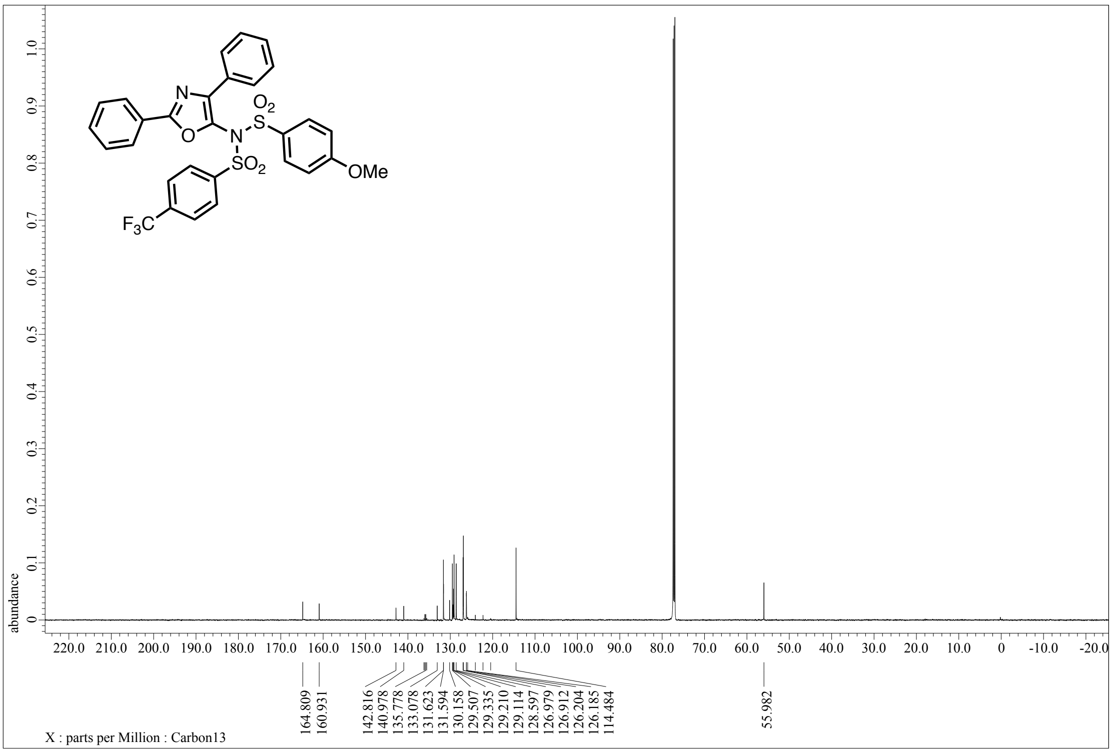

Figure S 47. ^{13}C NMR spectrum of **45**.

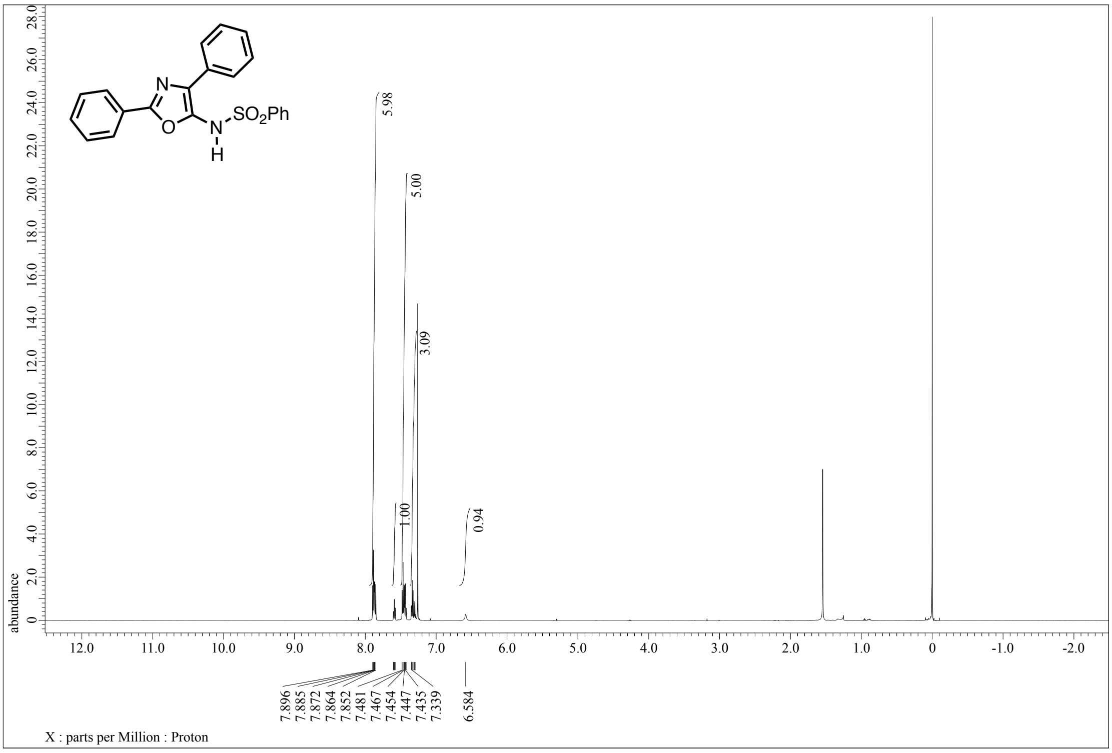

Figure S 48. ^{1}H NMR spectrum of **46**.

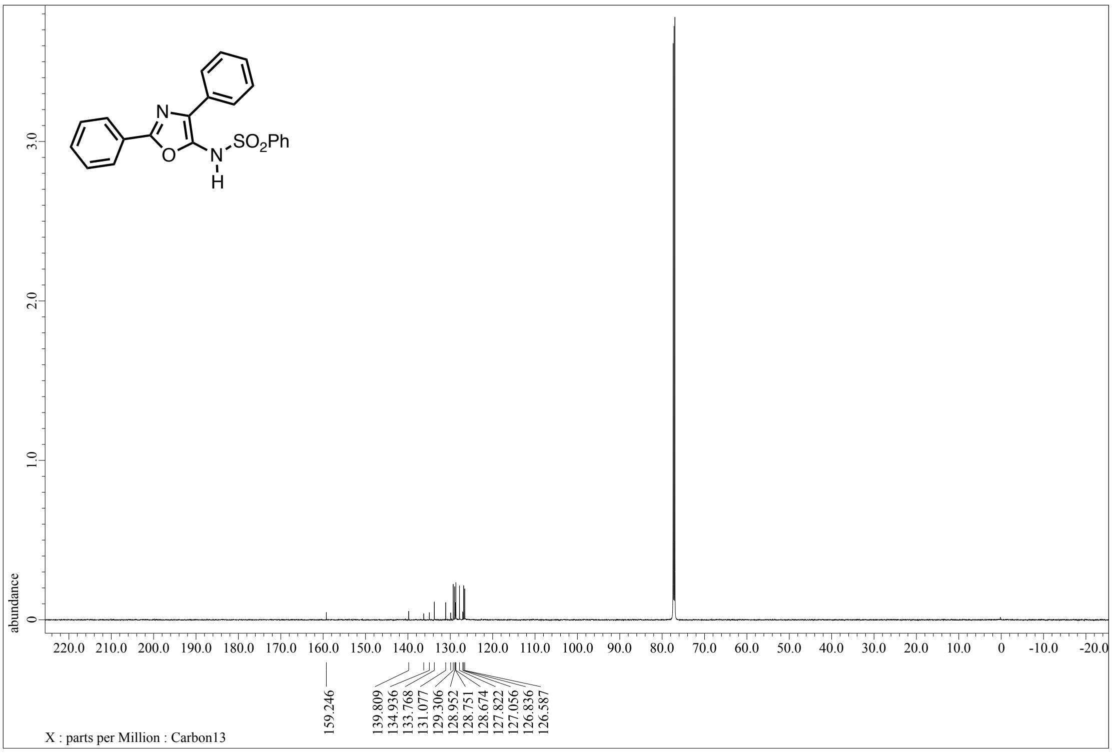

Figure S 49. ^{13}C NMR spectrum of **46**.

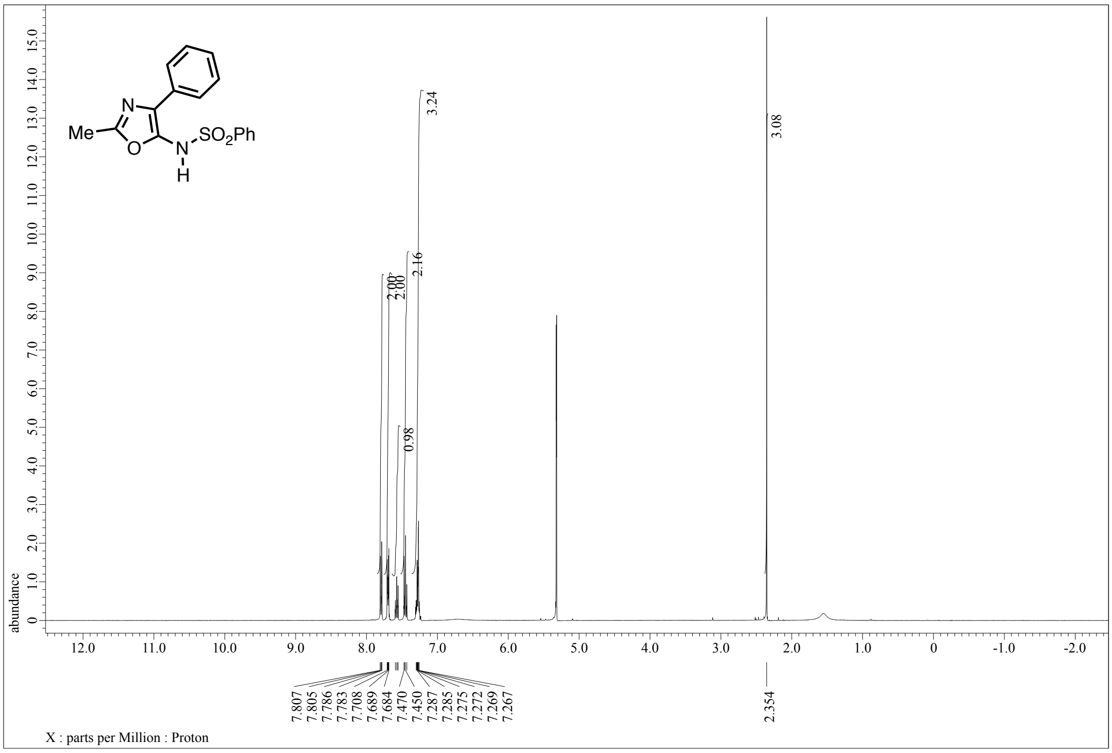

Figure S 50. ^{1}H NMR spectrum of **47**.

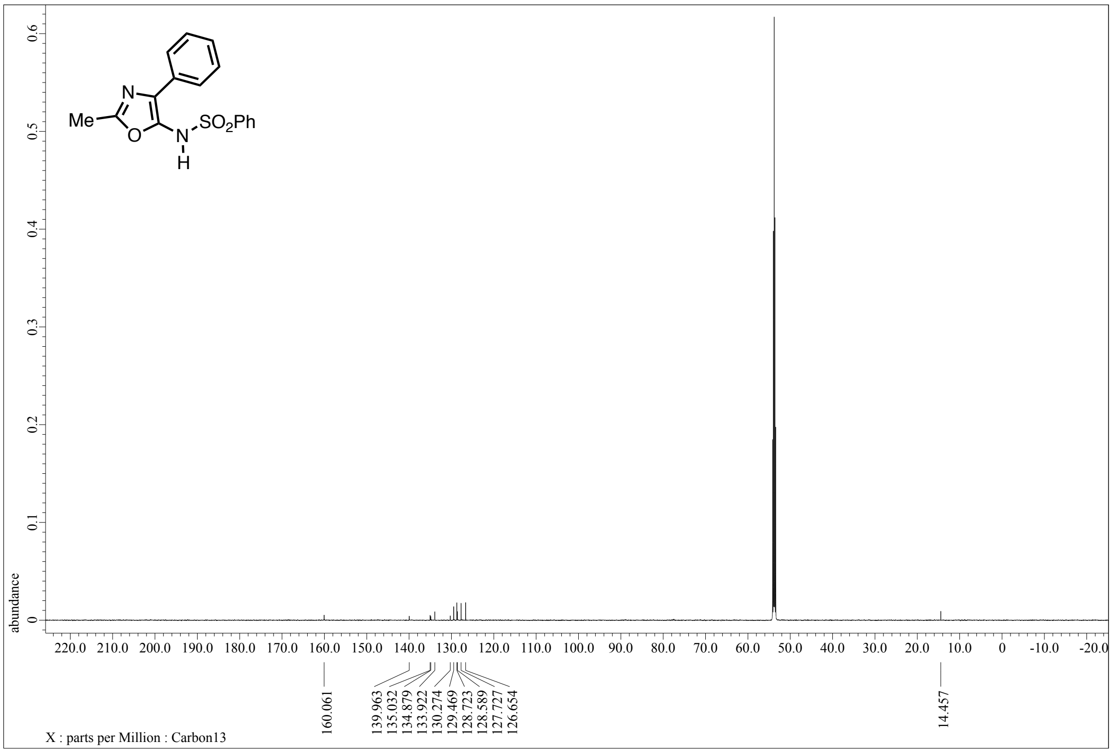

Figure S 51. ^{13}C NMR spectrum of **47**.

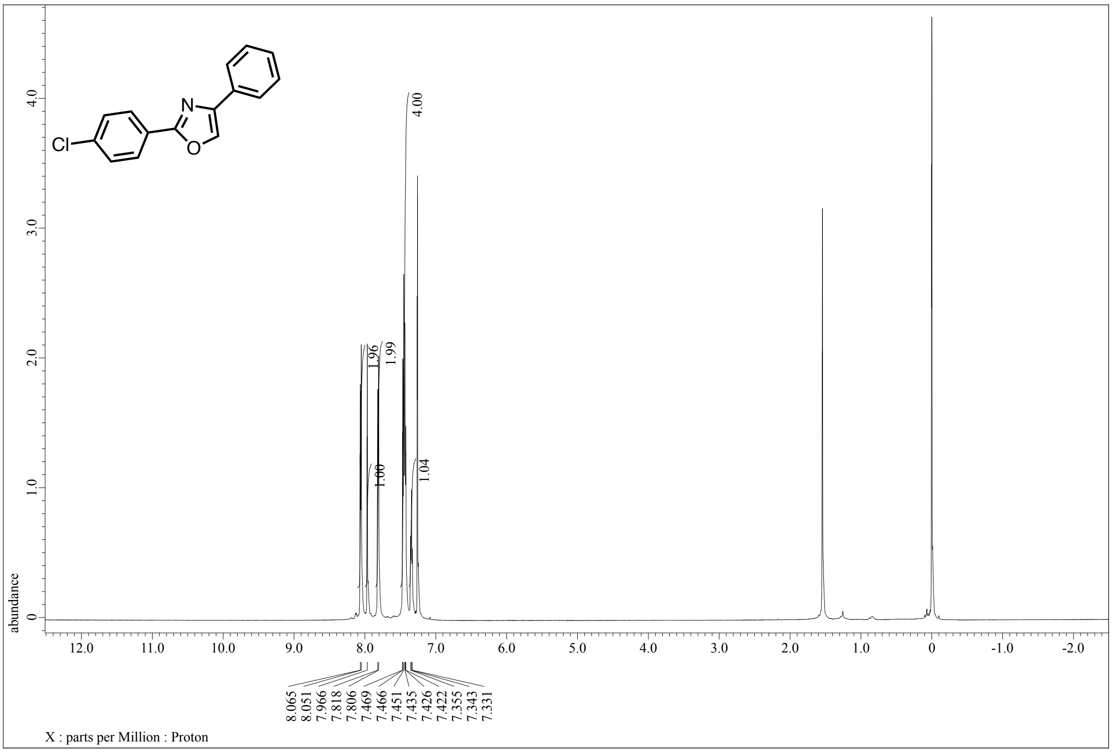

Figure S 52. ^{1}H NMR spectrum of **oxa1**.

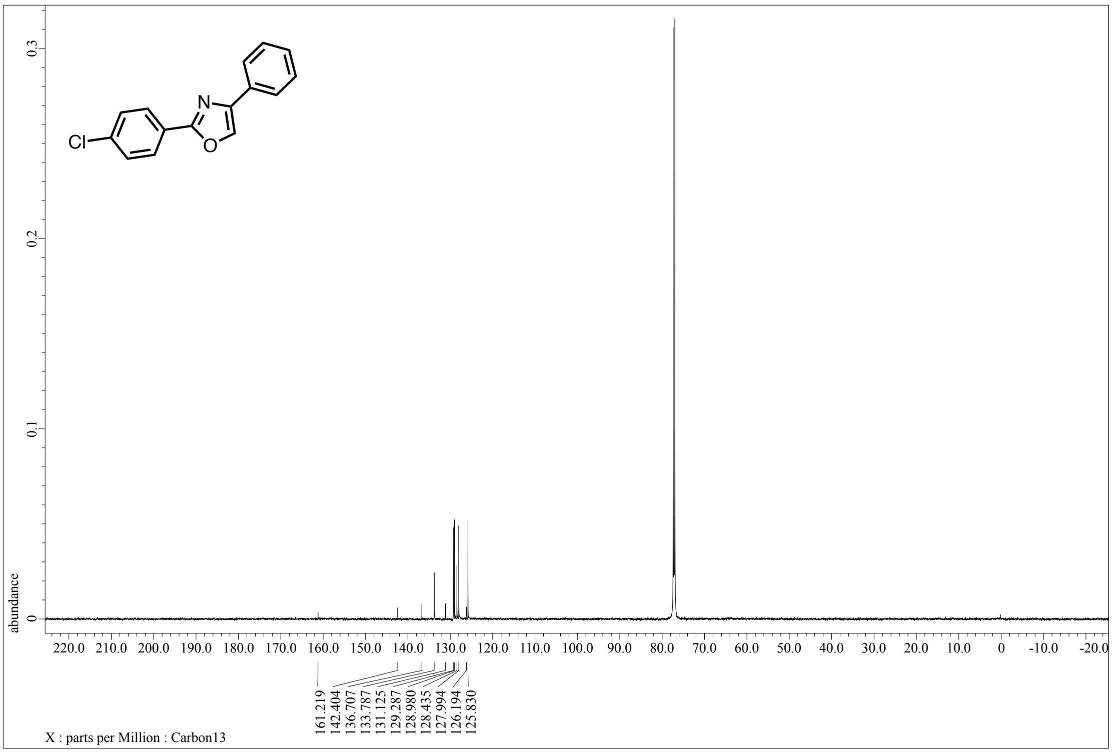

Figure S 53. ^{13}C NMR spectrum of **oxa1**.

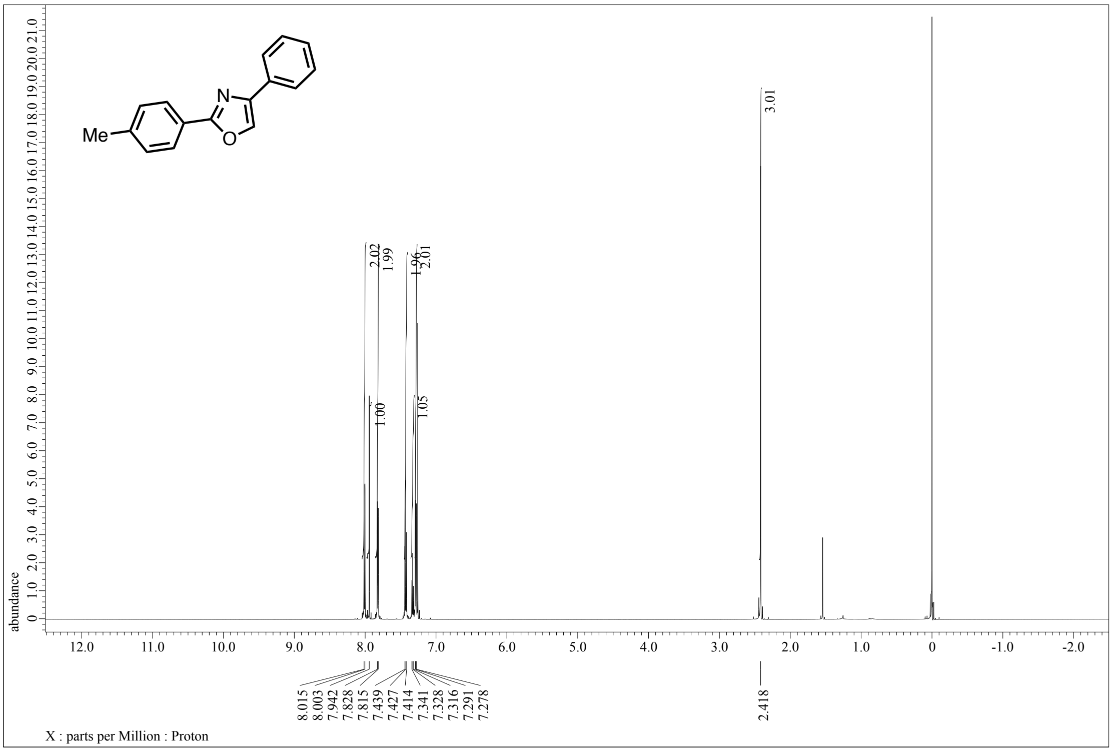

Figure S 54. ^{1}H NMR spectrum of **oxa2**.

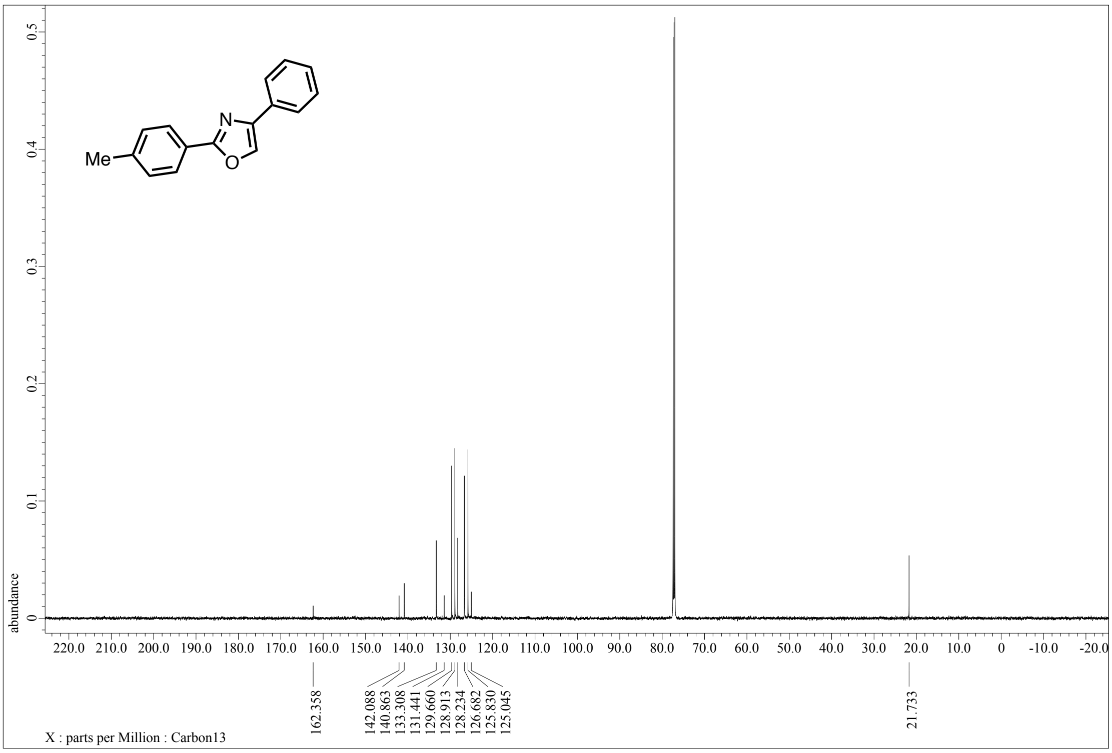

Figure S 55. ^{13}C NMR spectrum of **oxa2**.

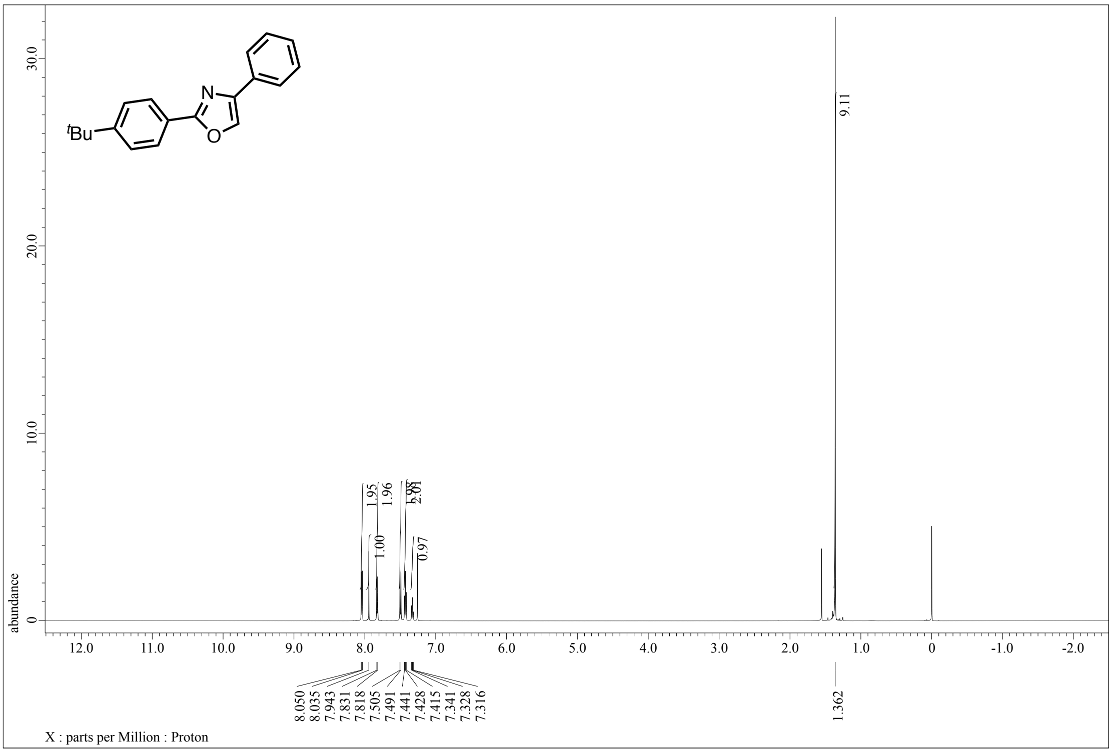

Figure S 56. ^{1}H NMR spectrum of **oxa3**.

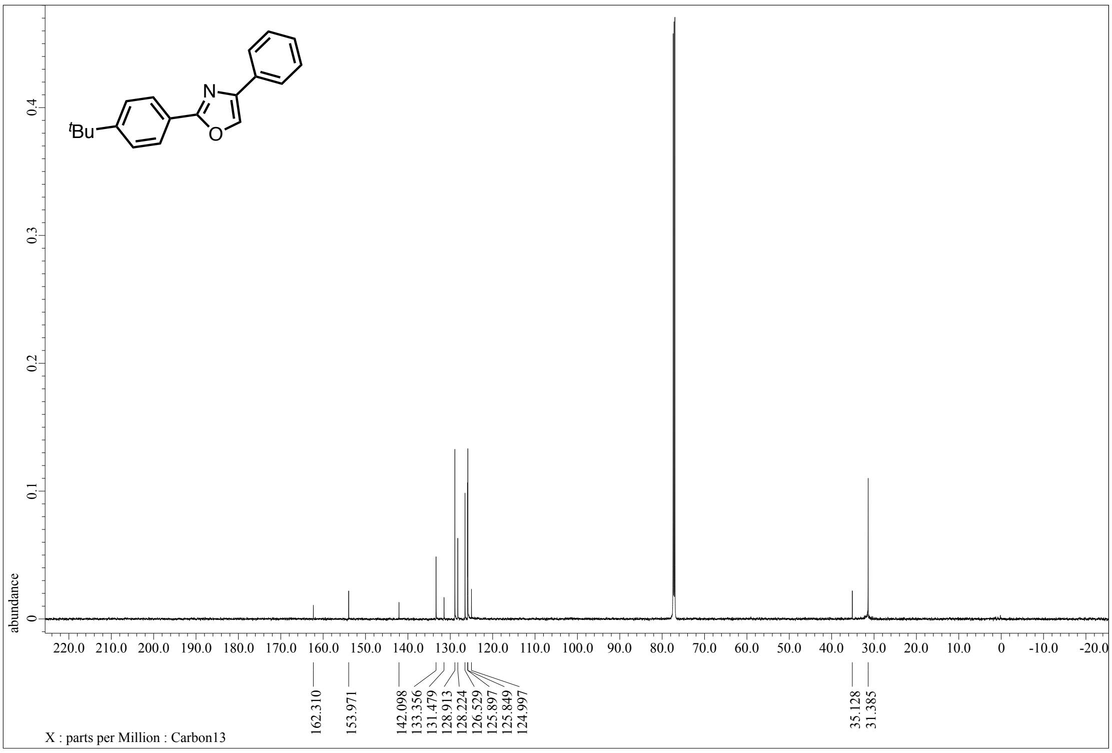

Figure S 57. ^{13}C NMR spectrum of **oxa3**.

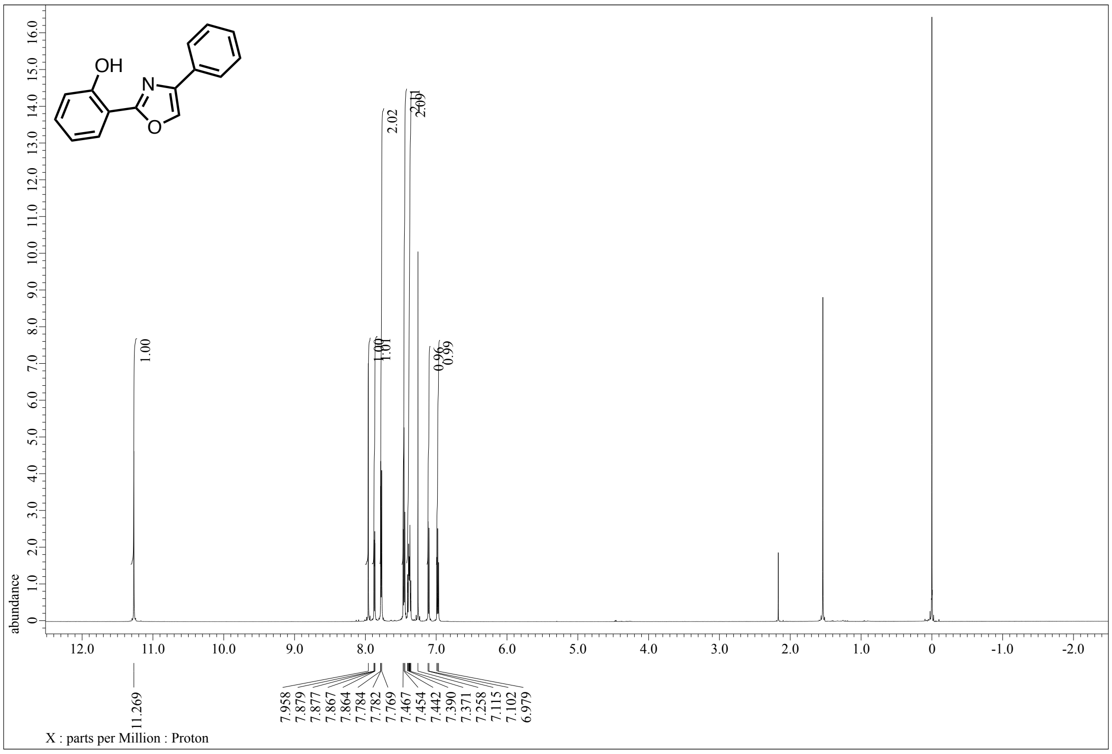

Figure S 58. ^{1}H NMR spectrum of **oxa4**.

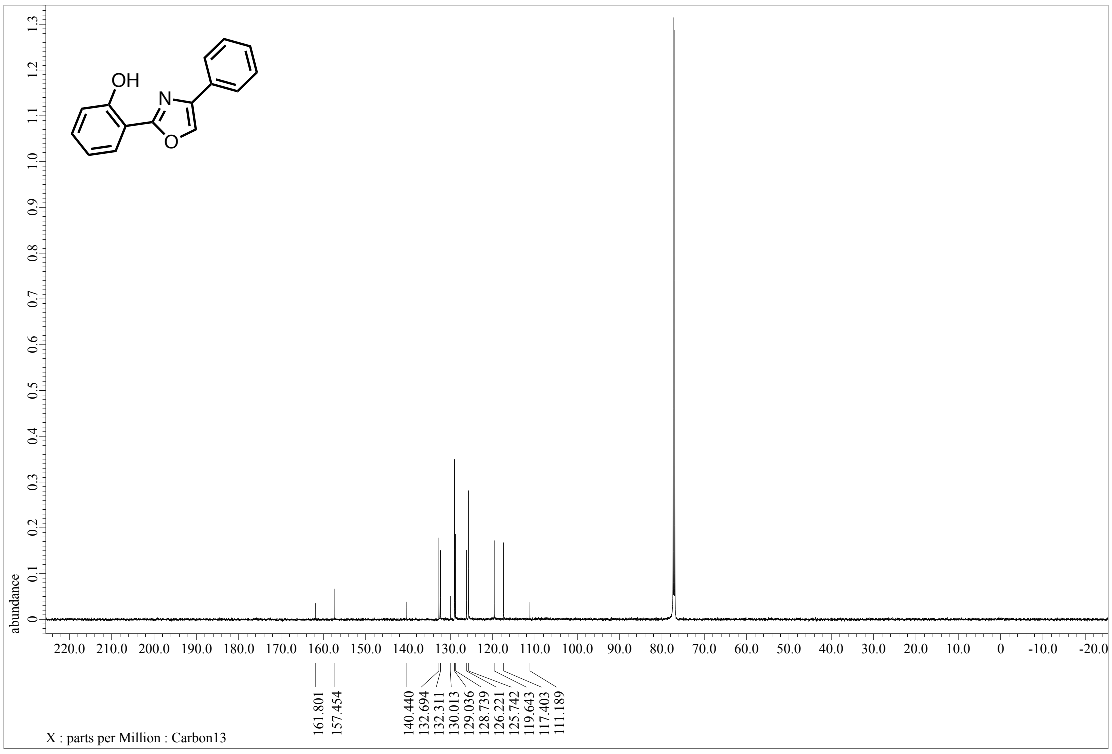

Figure S 59. ^{13}C NMR spectrum of **oxa4**.

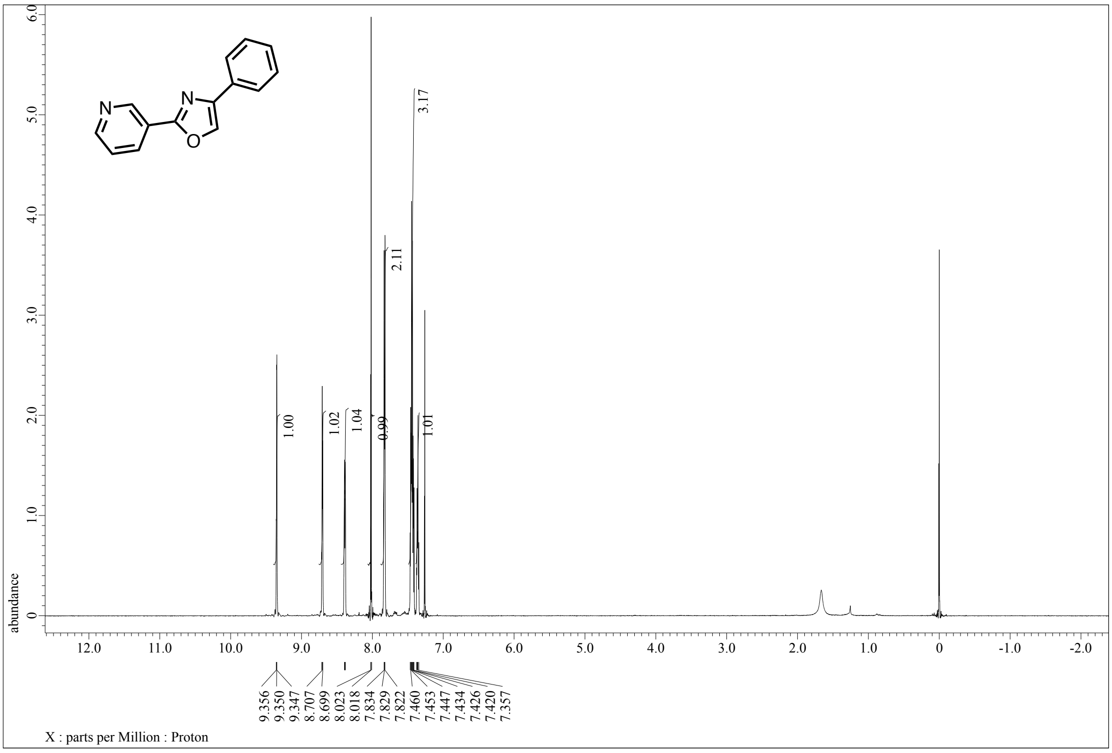

Figure S 60. ^{1}H NMR spectrum of **oxa5**.

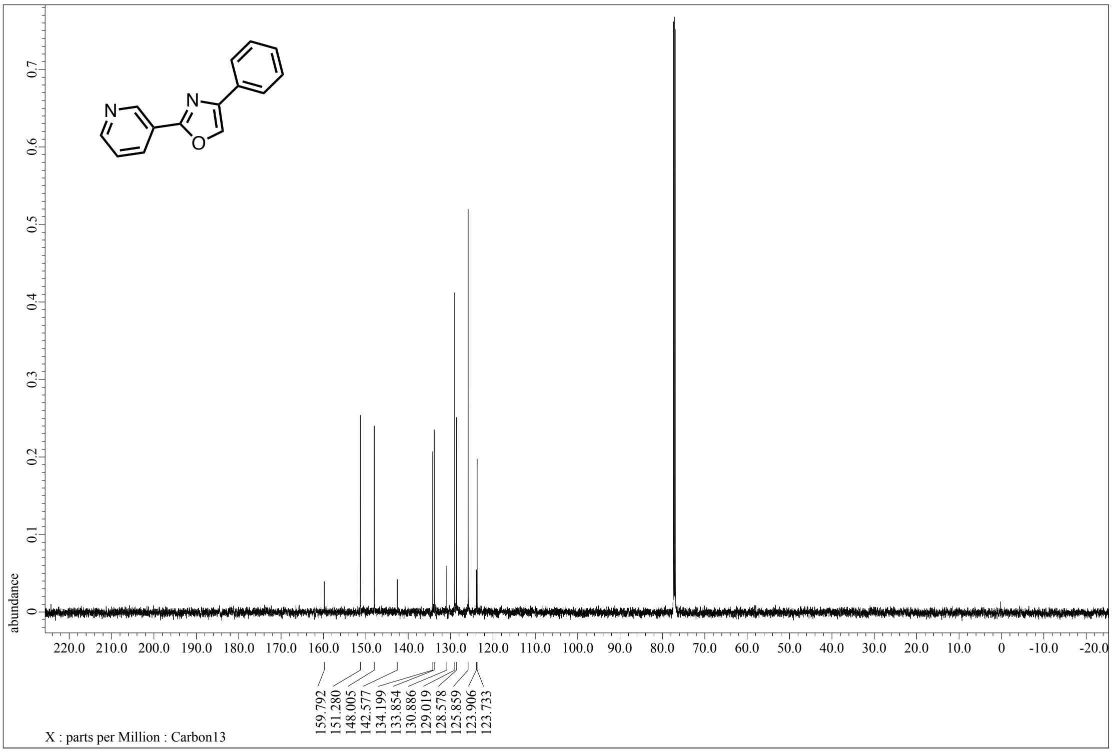

Figure S 61. ^{13}C NMR spectrum of **oxa5**.

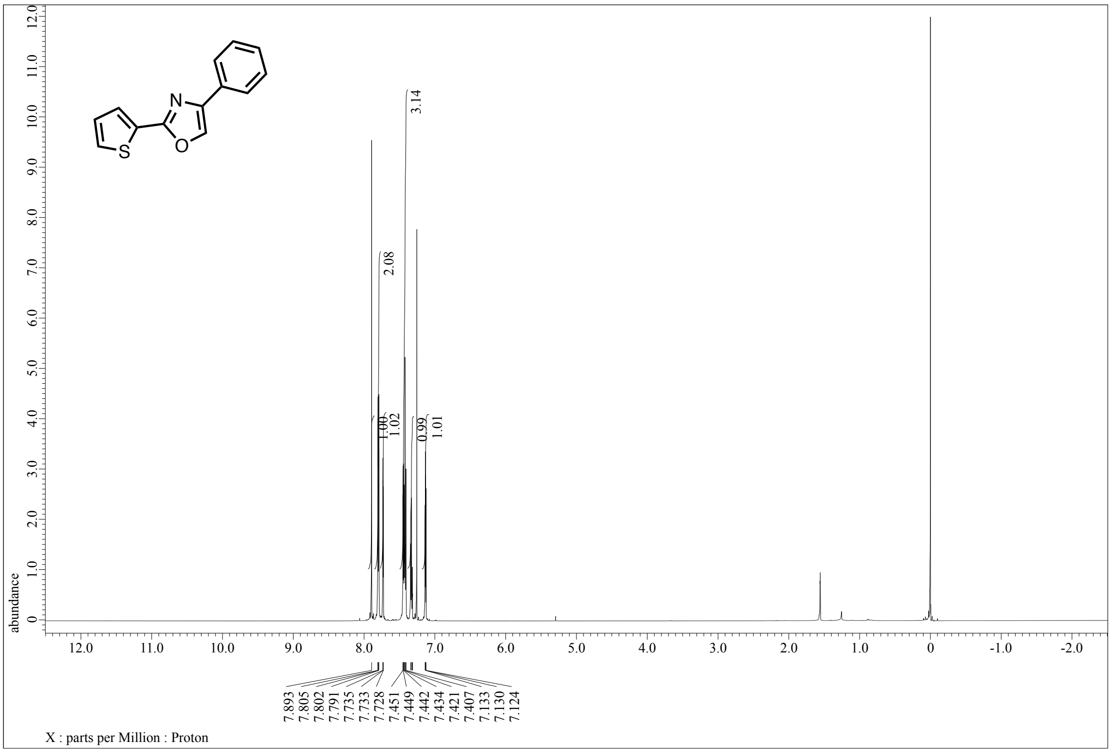

Figure S 62. ^{1}H NMR spectrum of **oxa6**.

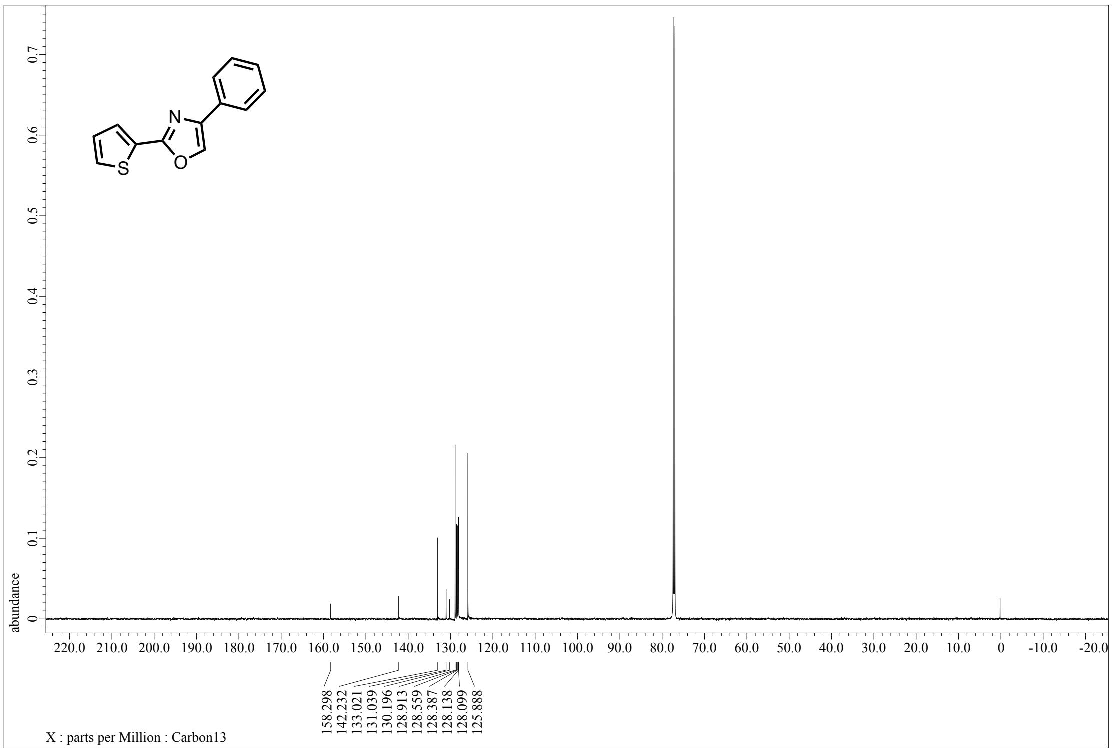

Figure S 63. ^{13}C NMR spectrum of **oxa6**.

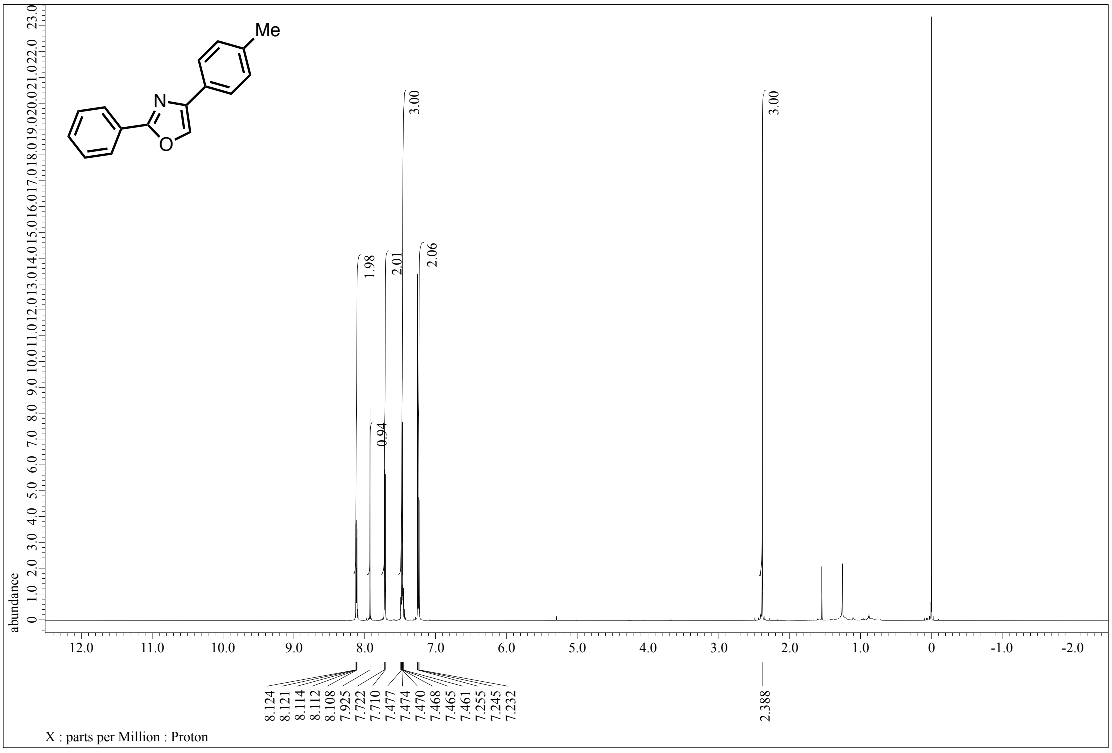

Figure S 64. ^{1}H NMR spectrum of **oxa7**.

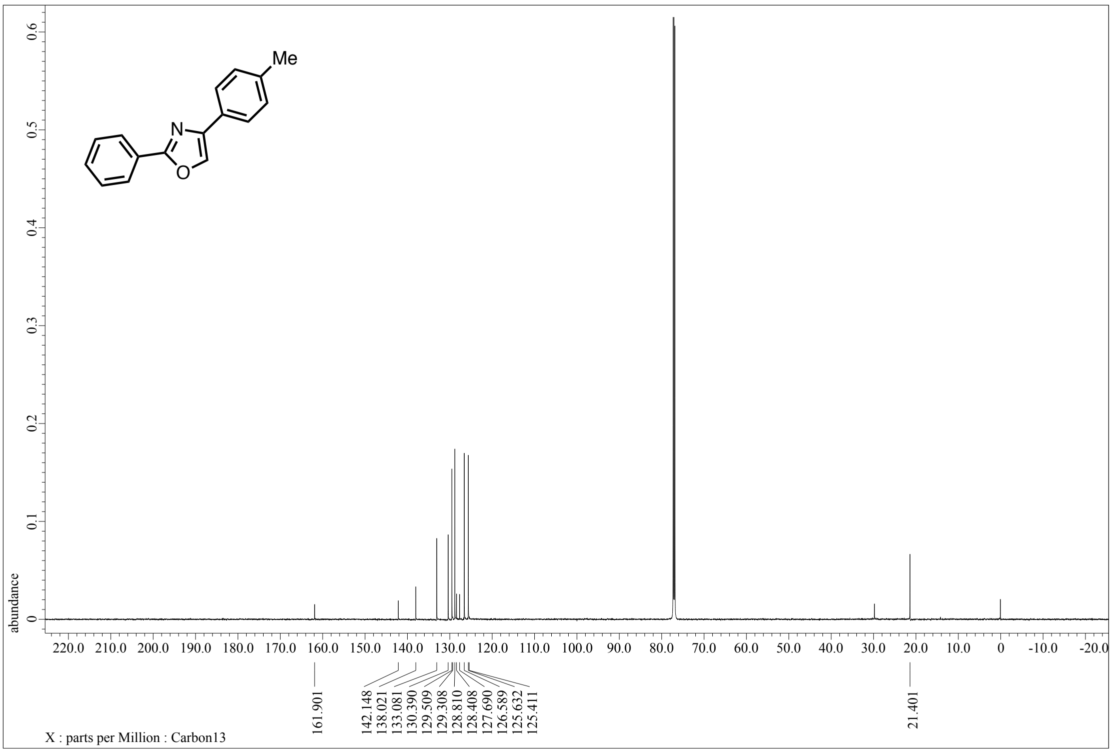

Figure S 65. ^{13}C NMR spectrum of **oxa7**.

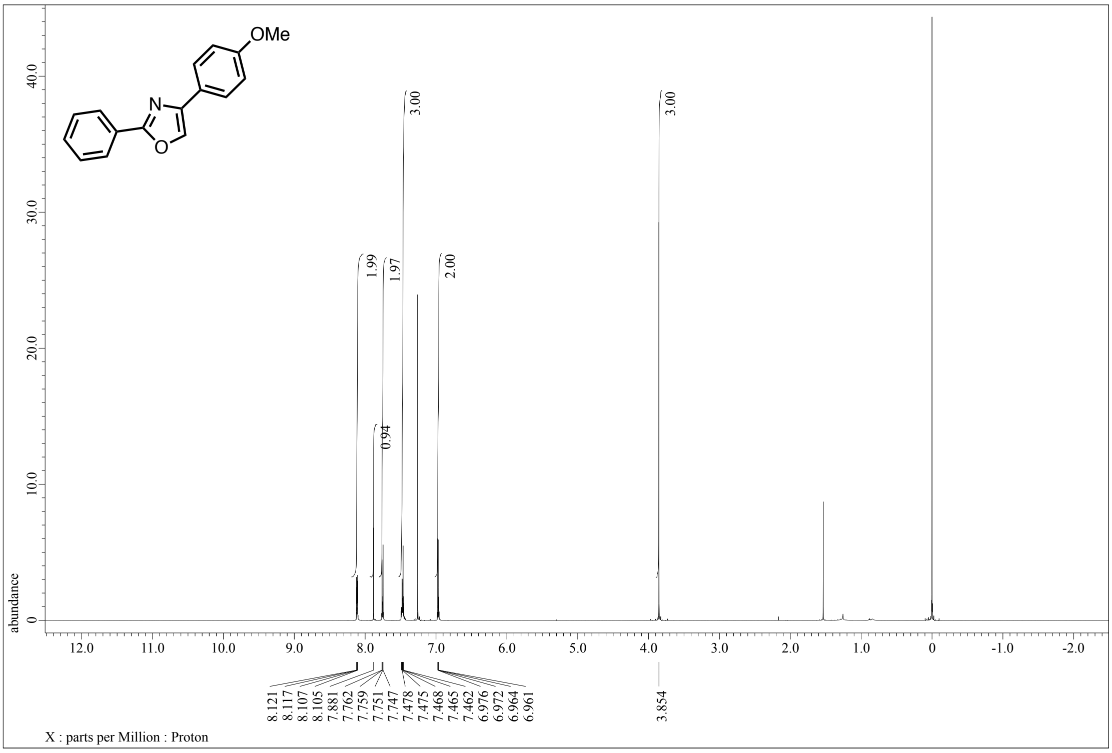

Figure S 66. ^{1}H NMR spectrum of **oxa8**.

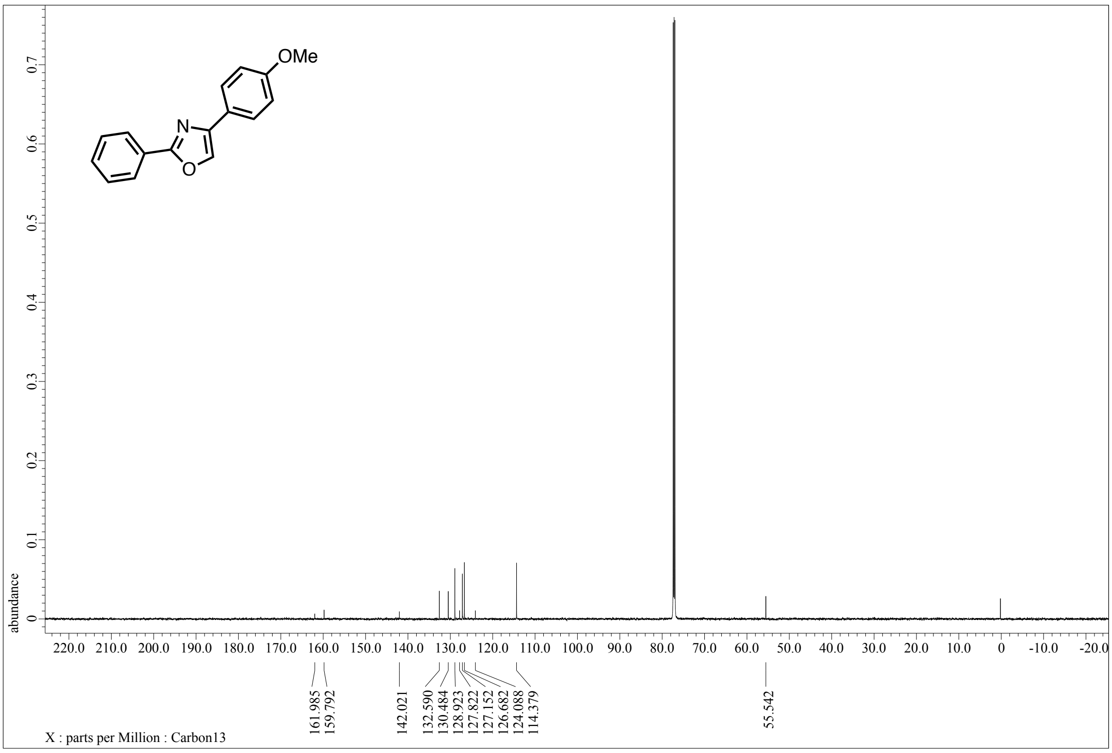

Figure S 67. ^{13}C NMR spectrum of **oxa8**.

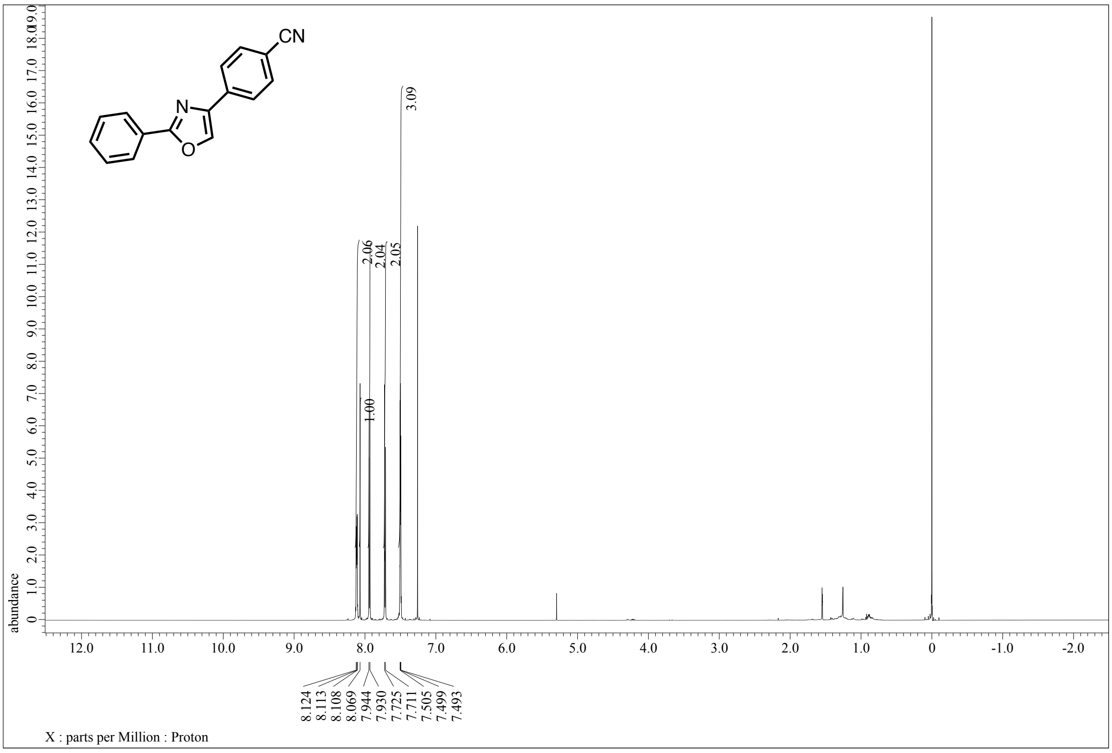

Figure S 68. ^{1}H NMR spectrum of **oxa9**.

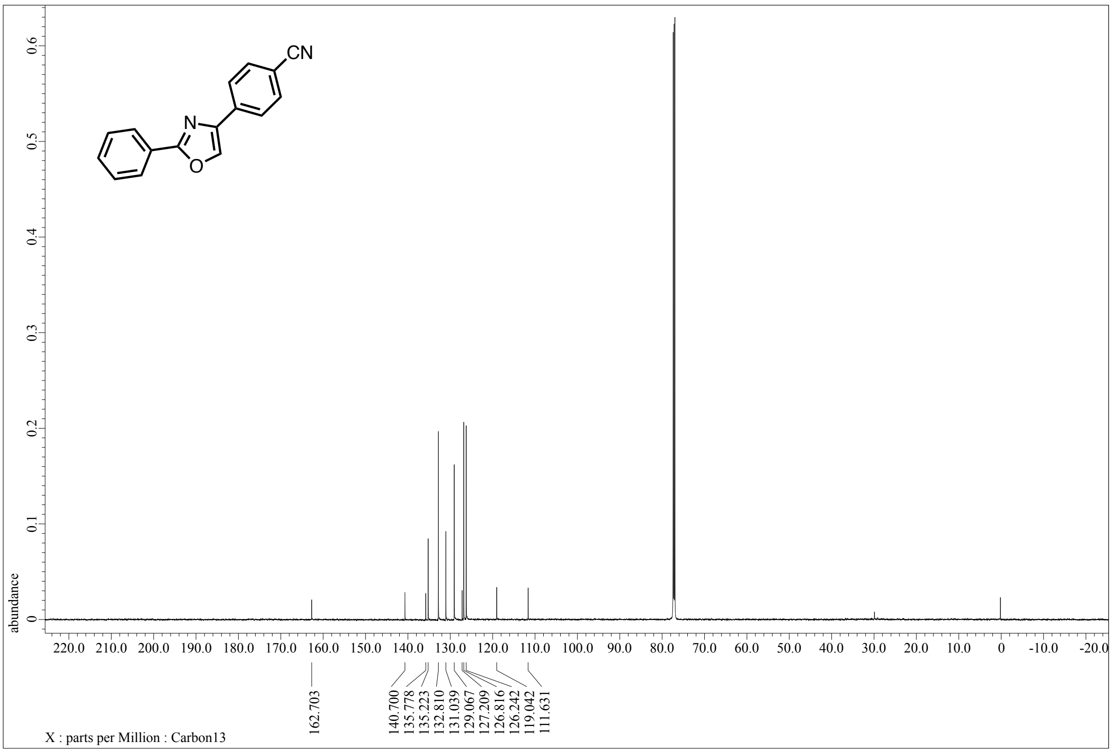

Figure S 69. ^{13}C NMR spectrum of **oxa9**.

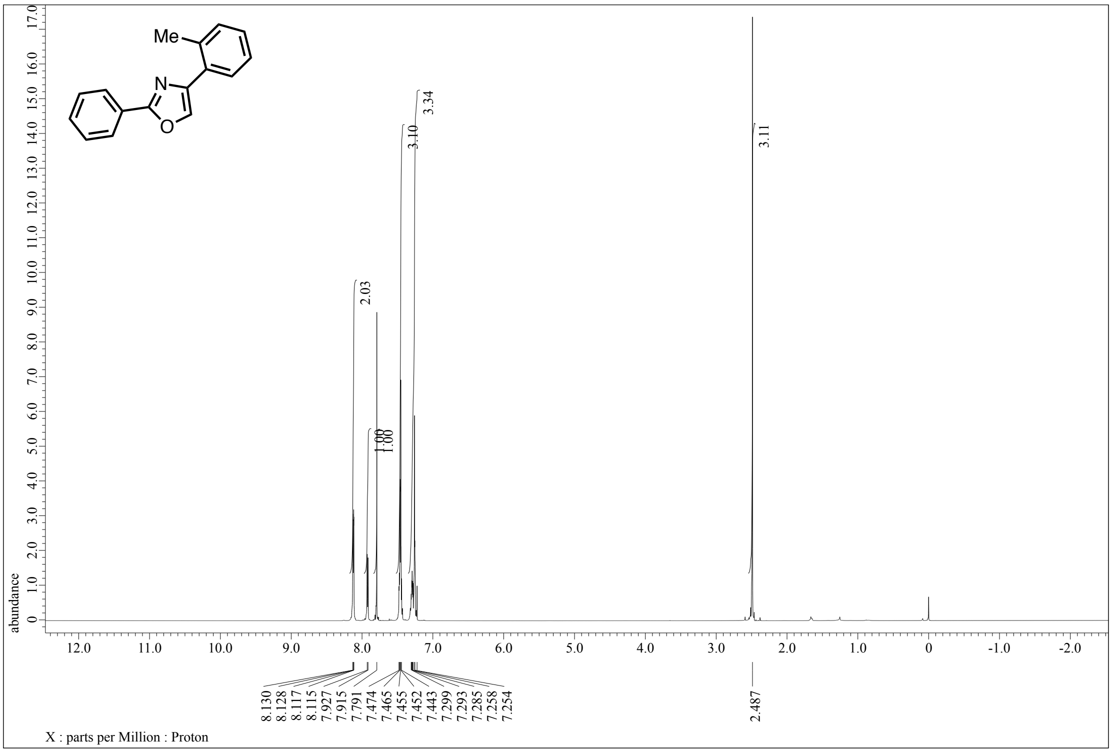

Figure S 70. ^{1}H NMR spectrum of **oxa10**.

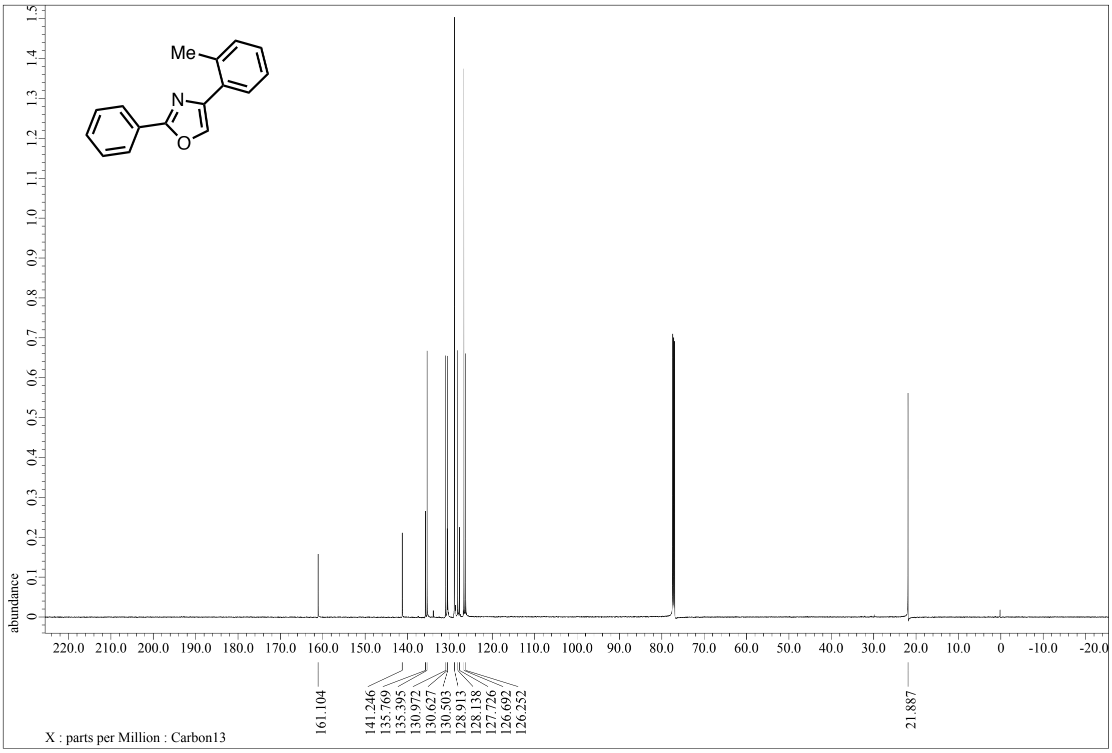

Figure S 71. ^{13}C NMR spectrum of **oxa10**.

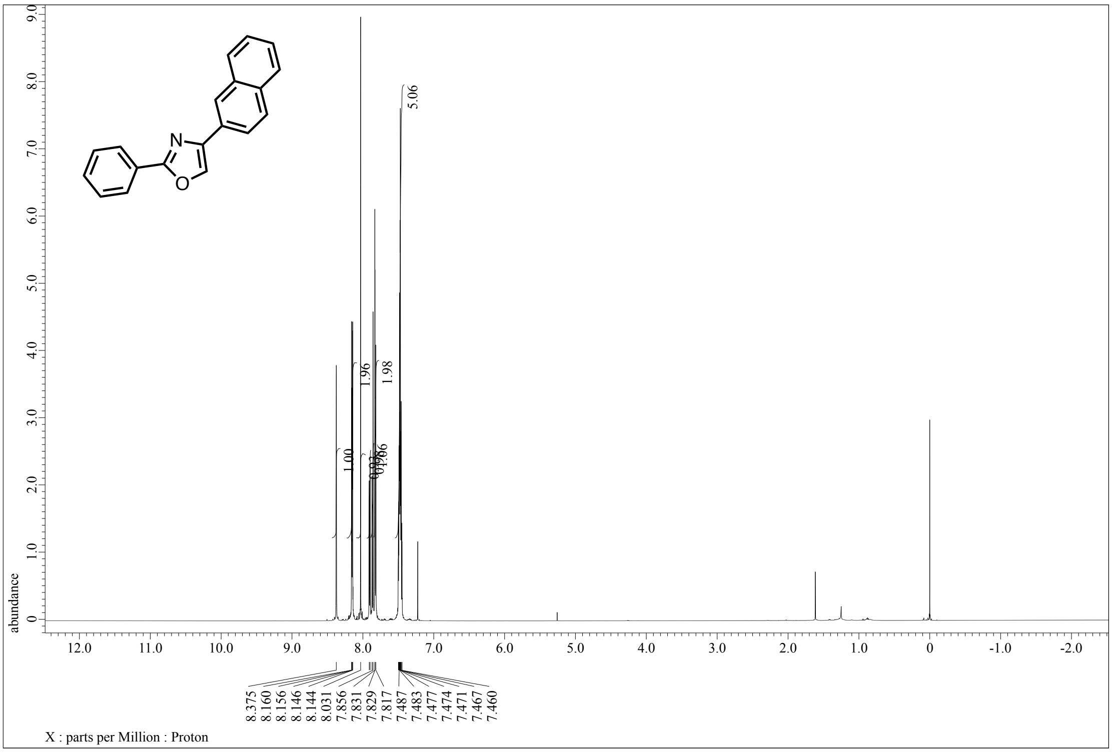

Figure S 72. ^{1}H NMR spectrum of **oxa11**.

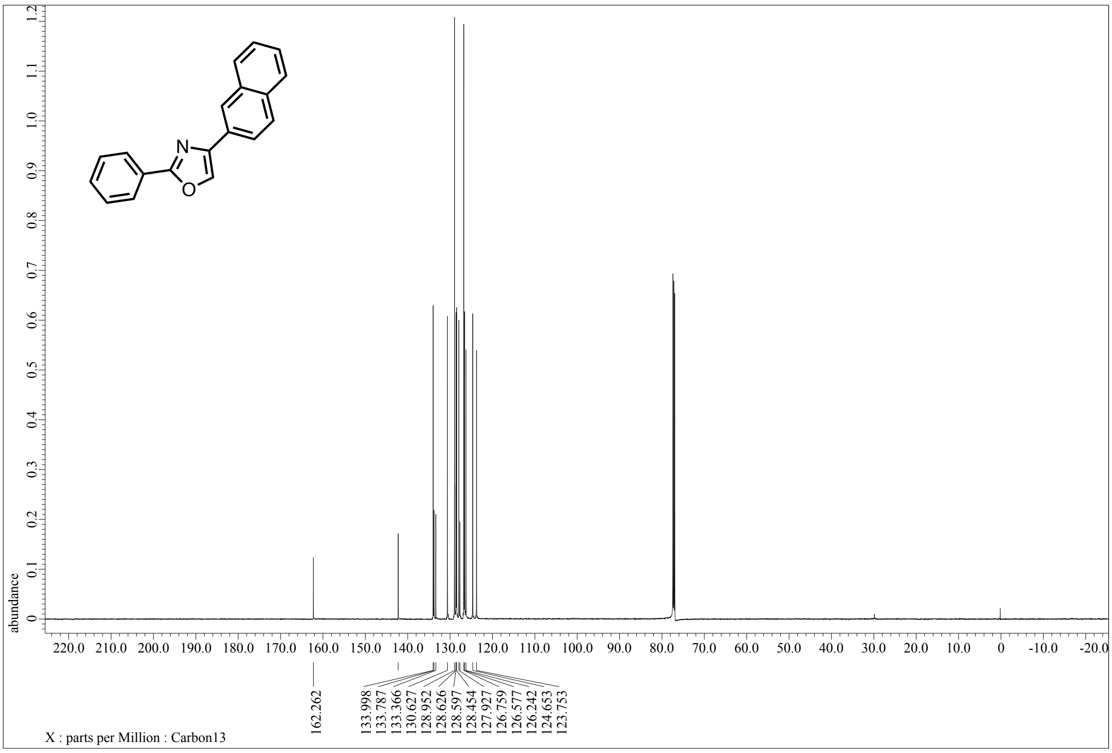

Figure S 73. ^{13}C NMR spectrum of **oxa11**.

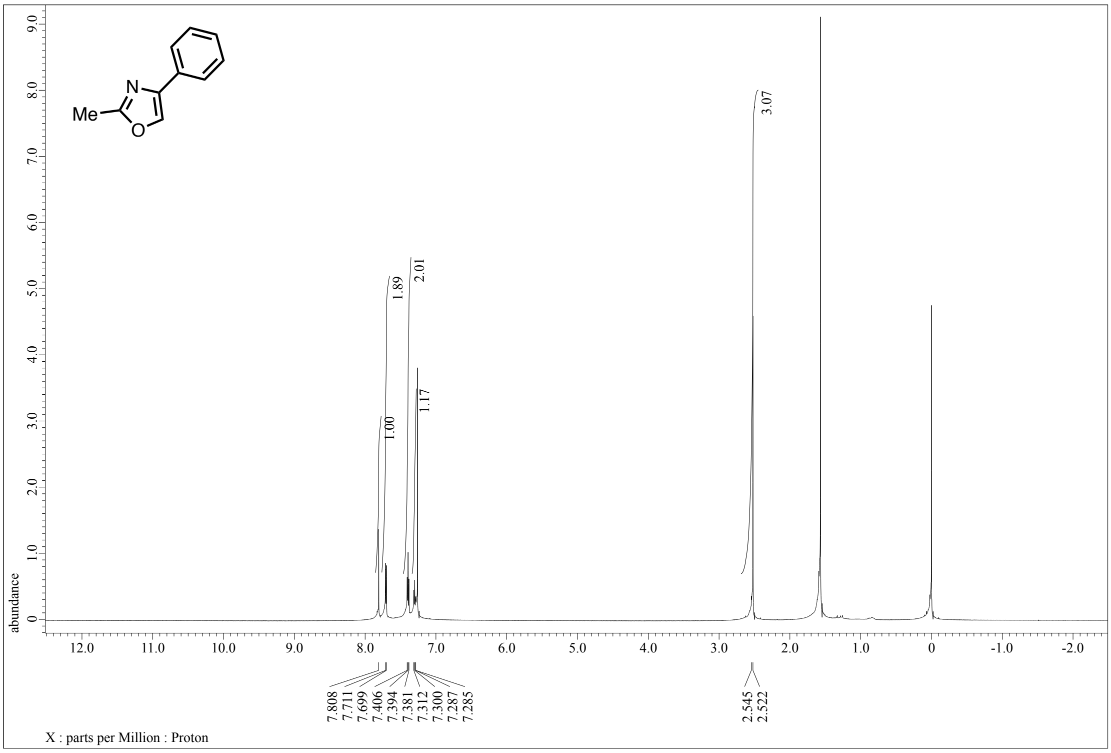

Figure S 74. ^{1}H NMR spectrum of **oxa12**.

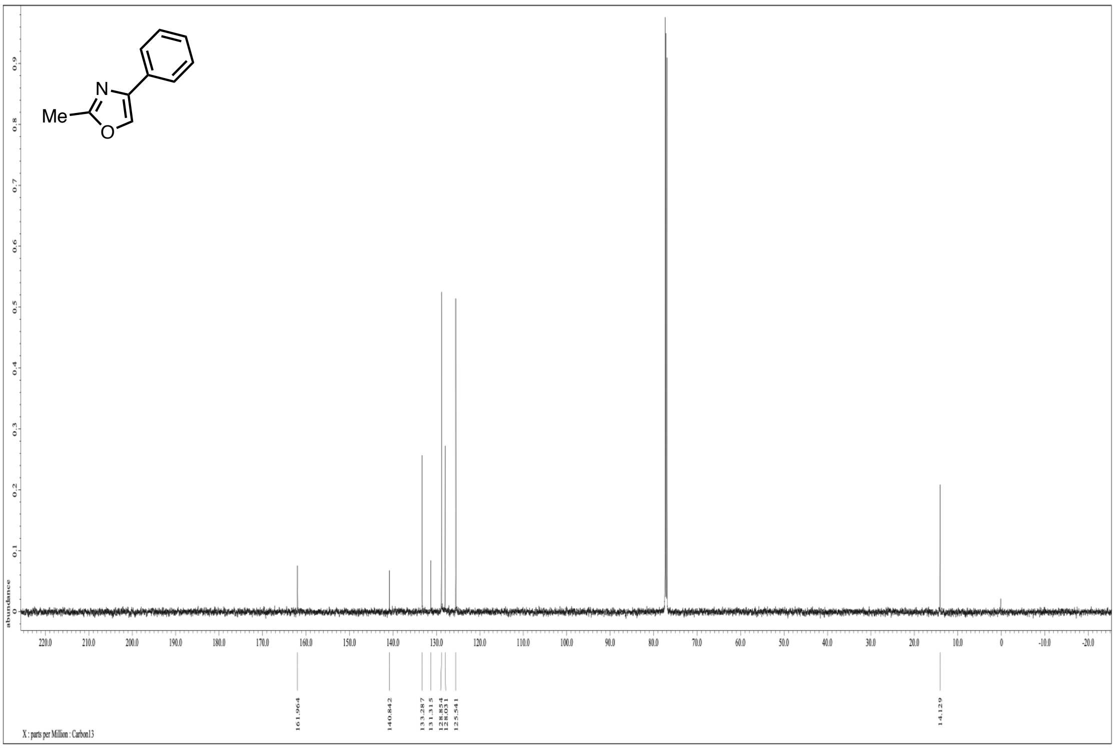

Figure S 75. ^{13}C NMR spectrum of **oxa12**.

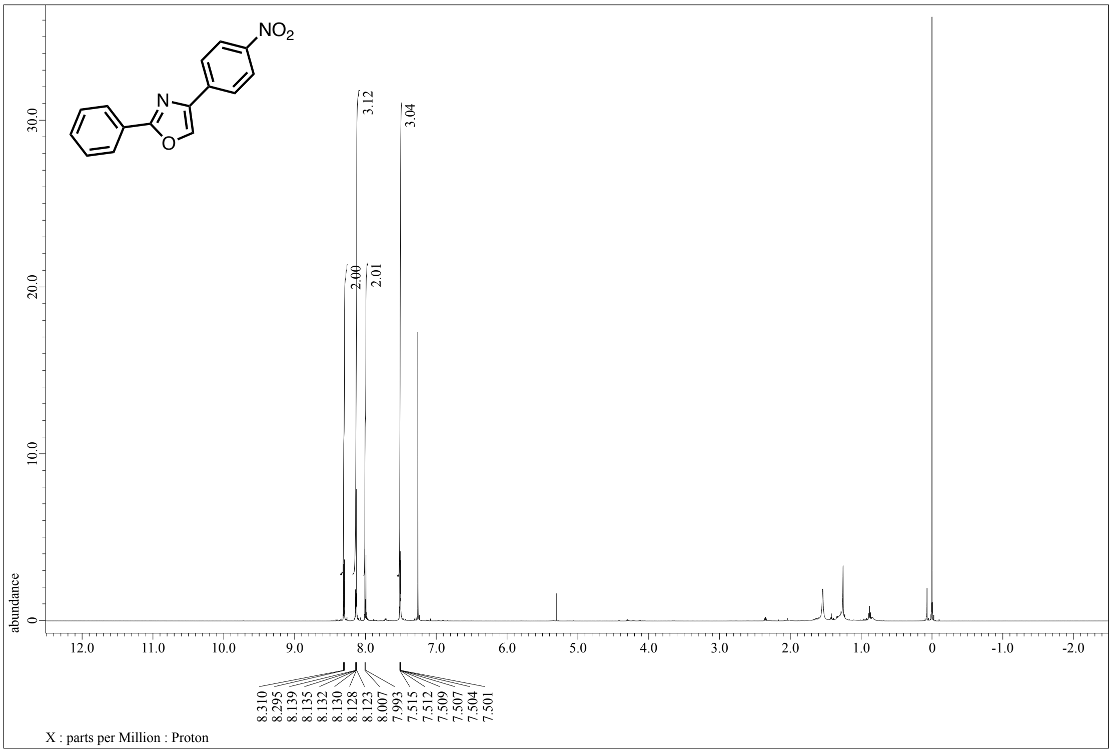

Figure S 76. ^{1}H NMR spectrum of **oxa13**.

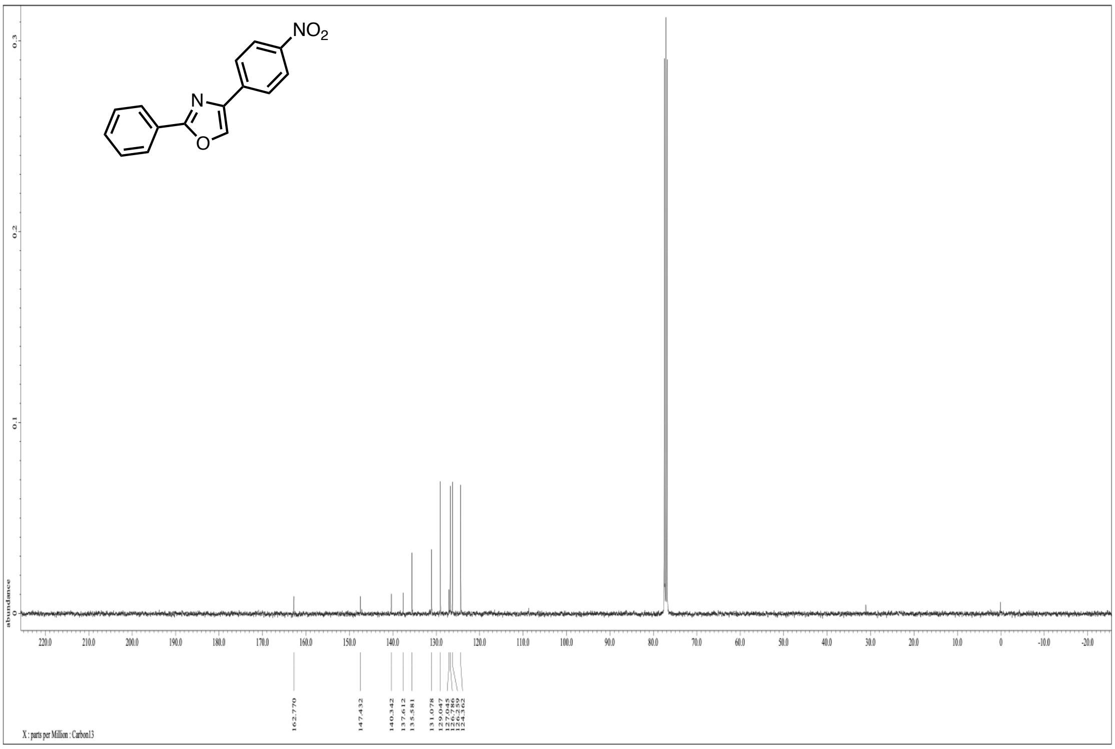

Figure S 77. ^{13}C NMR spectrum of **oxa13**.

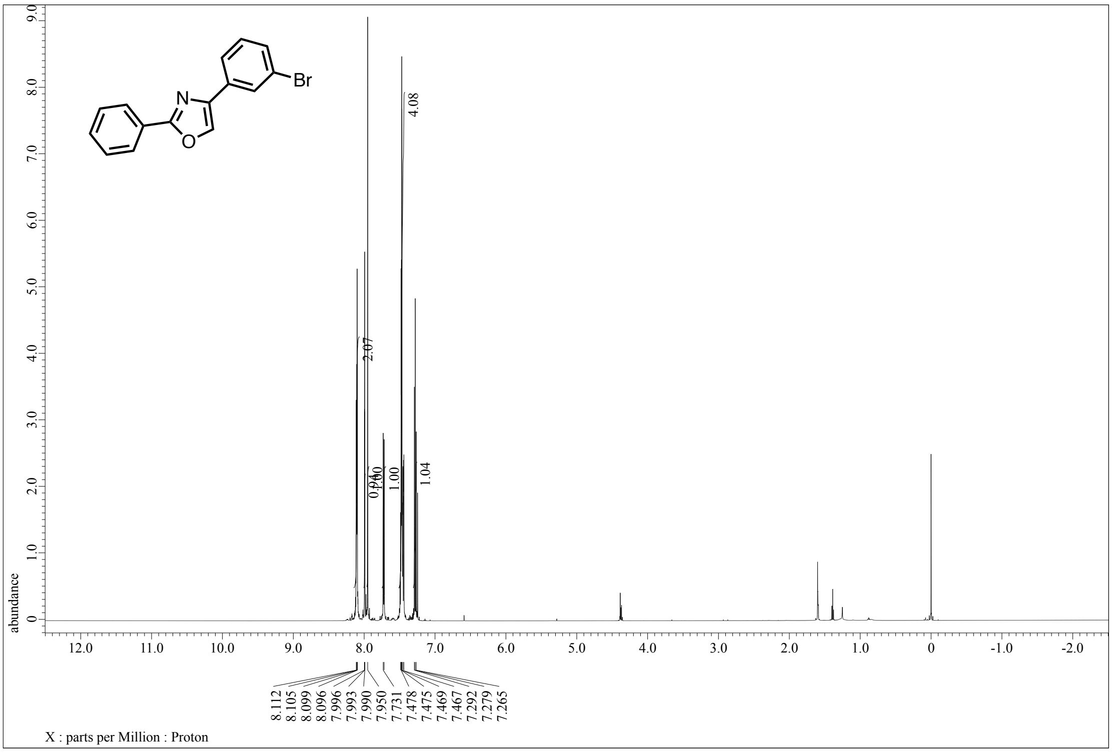

Figure S 78. ^{1}H NMR spectrum of **oxa14**.

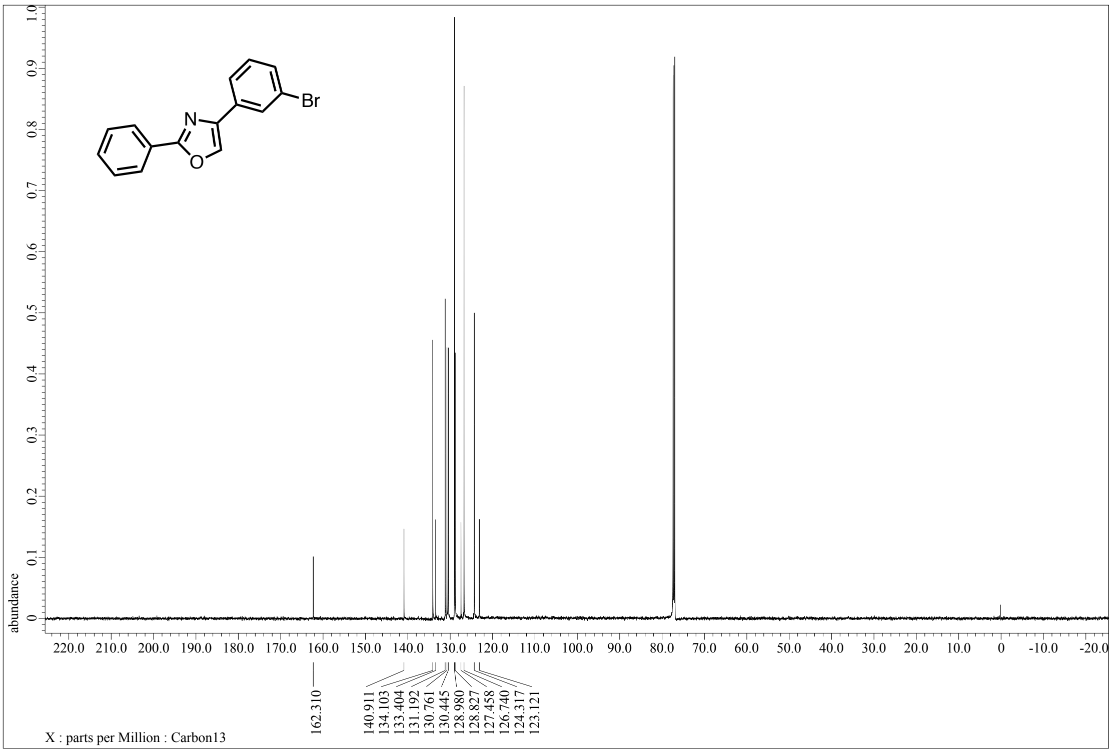

Figure S 79. ^{13}C NMR spectrum of **oxa14**.

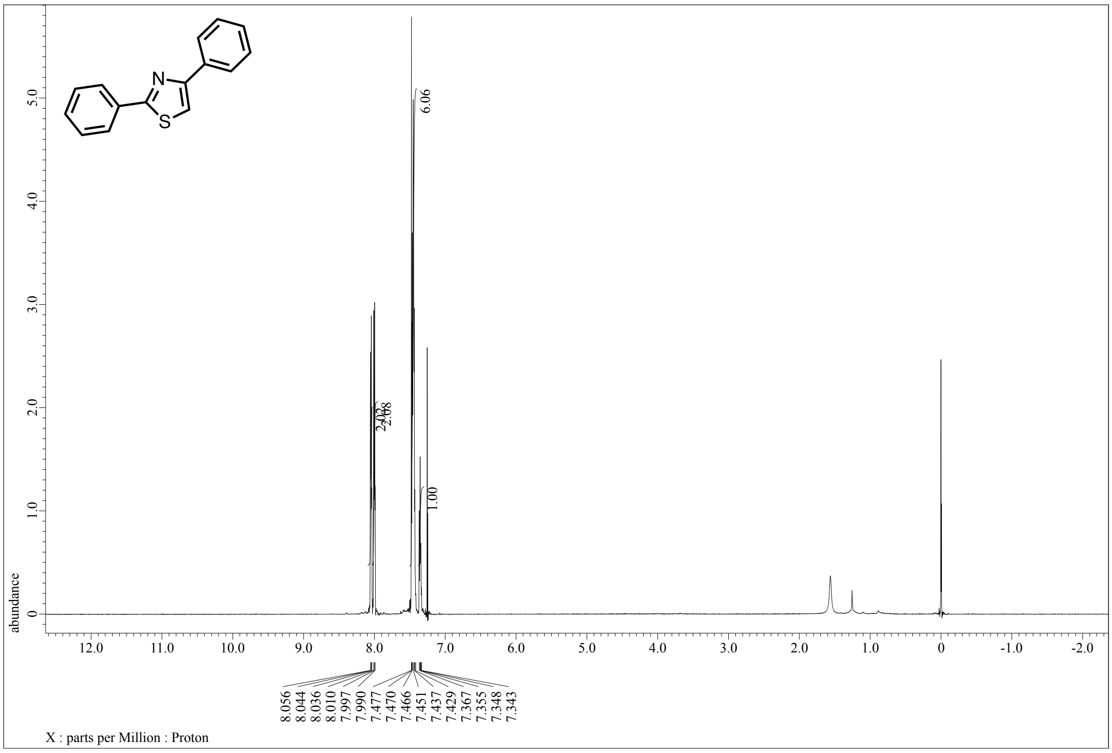

Figure S 80. ^{1}H NMR spectrum of **thia1**.

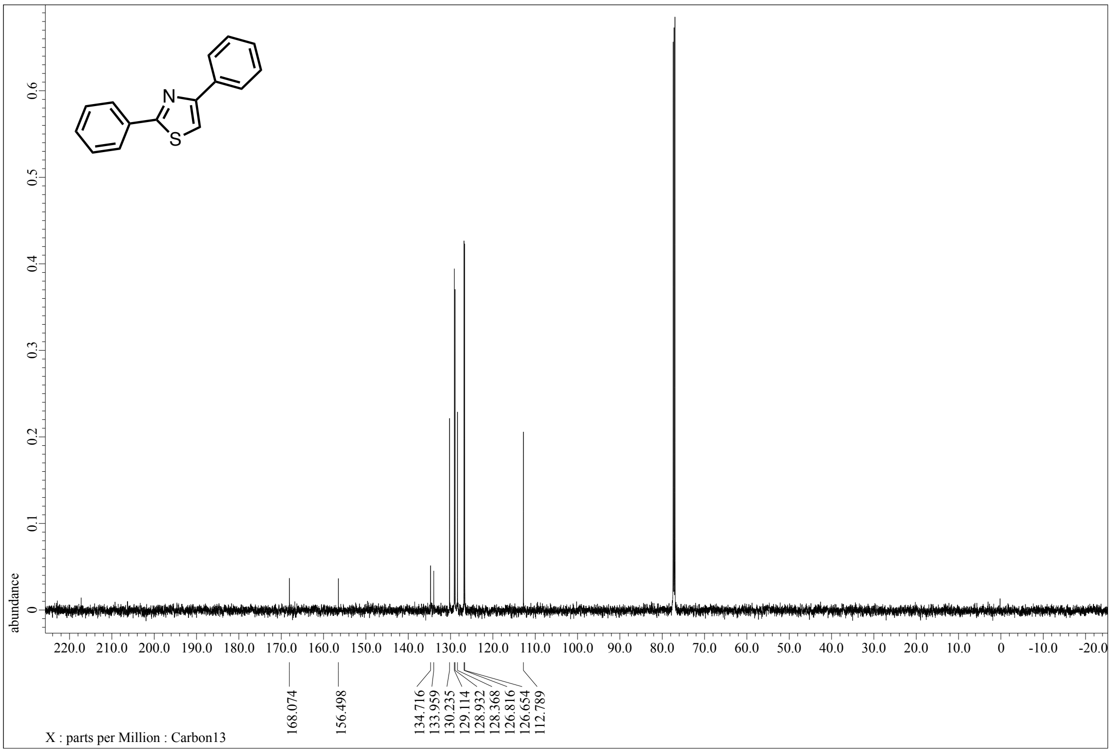

Figure S 81. ^{13}C NMR spectrum of **thia1**.

References

S1 Toh, S. *et al.* Identification and Characterization of Compounds that Affect Stomatal Movements. *Plant Cell Physiol.* **59**, 1568–1580 (2018).

S2 Takahashi, K., Hayashi, K. & Kinoshita, T. Auxin Activates the Plasma Membrane H^+ -ATPase by Phosphorylation during Hypocotyl Elongation in Arabidopsis. *Plant Physiol.* **159**, 632–641 (2012).

S3 Hayashi, M., Inoue, S.-i., Takahashi, K. & Kinoshita, T. Immunohistochemical Detection of Blue Light-Induced Phosphorylation of the Plasma Membrane H^+-ATPase in Stomatal Guard Cells. *Plant Cell Physiol.* **52**, 1238–1248 (2011).

S4 Besselièvre, F., Lebrequier, S., Mahuteau-Betzer, F. & Piguel, S. C-H Bond Activation: A Versatile Protocol for the Direct Arylation and Alkenylation of Oxazoles. *Synthesis* 3511–3518 (2009).

S5 Bailey, J. L. & Sudini, R. R. Synthesis of 2,4- and 2,4,5-Substituted Oxazoles via a Silver Triflate Mediated Cyclization. *Tetrahedron Lett.* **55**, 3674–3677 (2014).

S6 Narsaiah, A. V., Ghogare, R. S. & Biradar, D. O. Glycerin as Alternative Solvent for the Synthesis of Thiazoles. *Org. Commun.* **4**, 75–81 (2011).

S7 Kawakami, T., Murakami, K. & Itami, K. Catalytic C–H imidation of aromatic cores of functional molecules: ligand-accelerated Cu catalysis and application to materials- and biology-oriented aromatics. *J. Am. Chem. Soc.* **137**, 2460–2463 (2015).

S8 Sakakibara, Y., Ito, E., Kawakami, T., Yamada, S., Murakami, K. & Itami, K. Direct Coupling of Naphthalene and Sulfonimides Promoted by DDQ and Blue Light. *Chem. Lett.* **46**, 1014–1016 (2017).

S9 Ito, E., Fukushima, T., Kawakami, T., Murakami, K. & Itami, K. Catalytic Dehydrogenative C–H Imidation of Arenes Enabled by Photo-Generated Hole Donation to Sulfonimide. *Chem* **2**, 383–392 (2017).

S10 Haines, B. E., Kawakami, T., Kuwata, K., Murakami, K., Itami, K. & Musaev, D. G. Cu-Catalyzed Aromatic C–H Imidation with *N*-Fluorobenzenesulfonimide: Mechanistic Details and Predictive Models. *Chem. Sci.* **8**, 988–1001 (2017).

S11 Ferrer, E., Popkin, M. E. & Greaney, M. F. Suzuki Coupling of Oxazoles. *Org. Lett.* **8**, 2495–2498 (2006).

S12 Amaike, K., Itami, K. & Yamaguchi, J. Synthesis of Triarylpyridines in Thiopeptide Antibiotics by Using a C−H Arylation/Ring-Transformation Strategy. *Chem. Eur. J.* **22**, 4384–4388 (2016).

S13 Schuh, K. & Glorius, F. A Domino Copper-Catalyzed C-N and C-O Cross-Coupling for the Conversion- of Primary Amides into Oxazoles. *Synthesis* 2297–2306 (2007).

S14 Cheng, H.-T., Hou, R.-S., Wang, H.-M. & Chen, L.-C. Hypervalent Iodine(III) Sulfonate Reagent Mediated Synthesis of 4-Aryl-2-Phenyloxazoles in Ionic Liquid. *J. Chinese Chem. Soc.* 55, 919–922 (2013).

S 15 Tani, S. & Uehara, T. N., Yamaguchi, J. & Itami, K. Programmed Synthesis of Arylthiazoles through Sequential C–H Couplings. *Chem. Sci.* **5**, 123–135 (2014).
